# Combined Effects of Diet Quality Scores and Frailty on All-Cause Mortality and Life Expectancy in Middle-Aged and Older Adults

**DOI:** 10.3390/nu17193115

**Published:** 2025-09-30

**Authors:** Yang Yang, Huaicun Liu, Liangkai Chen, Filippos T. Filippidis

**Affiliations:** 1 Department of Primary Care and Public Health, School of Public Health, Imperial College London, London W12 0BZ, UK; f.filippidis@imperial.ac.uk; 2 Department of Human Anatomy, Histology and Embryology, School of Basic Medical Sciences, Peking University, Beijing 100191, China; liuhc@bjmu.edu.cn; 3 Hubei Key Laboratory of Food Nutrition and Safety, Department of Nutrition and Food Hygiene, School of Public Health, Tongji Medical College, Huazhong University of Science and Technology, Wuhan 430030, China; clk@hust.edu.cn; 4 Ministry of Education Key Lab of Environment and Health, School of Public Health, Tongji Medical College, Huazhong University of Science and Technology, Wuhan 430030, China

**Keywords:** diet quality score, frailty, mortality, life expectancy, UK Biobank

## Abstract

**Background:** Frailty is known to elevate the risk of all-cause mortality and shorten life expectancy. Although the effects of diet on health are well documented, the specific interaction between diet quality and frailty remains unexplored. This research aims to examine the combined effects of various diet quality scores and frailty on all-cause mortality and life expectancy among middle-aged and older adults. **Methods:** A total of 151,628 participants were sourced from the UK Biobank for analysis. Frailty phenotype (FP) and frailty index (FI), as two different approaches, were used to assess frailty status. Diet quality was evaluated through seven diet quality scores: the Alternate Healthy Eating Index (AHEI), Dietary Approaches to Stop Hypertension (DASH) score, Mediterranean diet (MED) score, Dietary Inflammatory Index (DII), and three plant-based diet indices (overall PDI, healthful PDI, and unhealthful PDI). Cox proportional hazards models were applied to calculate adjusted hazard ratios (HRs) for overall mortality and predict life expectancy differences. **Results:** Over a median follow-up period of 12.2 years, 8231 deaths were identified. After accounting for potential confounding factors, frail individuals in the unhealthier tertile of diet scores exhibited markedly elevated mortality risks, ranging from 1.99 to 2.07 based on the frailty index and 2.79 to 3.06 based on the frailty phenotype, compared to their robust counterparts in the healthier tertile. Regardless of frailty categories, a healthier diet was associated with longer life expectancy and with lower mortality risk in a dose–response relationship. **Conclusions:** The healthier tertile of diet scores was found to mitigate the detrimental effects of frailty, emphasizing diet quality as a modifiable factor in promoting healthier aging. Evidence suggests that it is never too late to adopt healthier dietary habits for significant health benefits.

## 1. Introduction

According to projections by the World Health Organization, the global population over 60 years of age is expected to double by 2050 compared to the year 2000, while those above 80 years are anticipated to nearly quadruple [1]. This significant demographic shift, alongside increasing life expectancy, underscores the importance of promoting healthy aging among middle-aged and older adults as critical public health priorities. Within these populations, frailty is notably prevalent and becomes more common with advancing age [2]. Frailty has been linked with numerous adverse outcomes, including elevated risks of hospitalization, falls, fractures, disabilities, morbidities, and mortality [3,4,5,6], posing a significant challenge to healthcare systems worldwide. Thus, addressing frailty is crucial for improving survival and prolonging life expectancy.

Although mortality has long been the primary endpoint in epidemiological research, life expectancy provides an even more intuitive and policy-relevant measure of population health. Estimating gains or losses in life expectancy according to modifiable risk factors allows for clearer communication of their long-term public health impact. Prior studies have shown that lifestyle factors such as smoking, physical activity, and alcohol consumption are strongly associated with variations in life expectancy [7,8]. However, evidence regarding the combined influence of diet quality and frailty on life expectancy remains limited, particularly in aging populations.

Diet plays a pivotal role in modulating the complex process of aging, driven by various processes of molecular damage [9]. Recently, research has shifted from analyzing individual nutrients and specific food groups to a more holistic examination of overall diet quality, recognizing the intricate interplay and synergism among various nutrients and foods consumed [10,11]. By calculating diet quality scores based on predefined criteria or dietary guidelines [12,13], which reward healthy food consumption and penalize unhealthy choices [14], the complexities of real-world diets and their interactions are better captured, allowing for a more accurate evaluation of diet–disease relationships [15]. Higher diet quality scores have been shown to be inversely associated with a range of chronic diseases and premature mortality [16,17].

However, important gaps remain. First, different diet quality scores capture distinct dietary dimensions, and their comparability in relation to mortality outcomes is not fully understood. Second, frailty, as a measure of biological aging and physiological reserve, may modify or interact with dietary effects, but few studies have systematically examined these joint associations. Understanding how diet quality and frailty together influence life expectancy is crucial for designing preventive strategies that not only reduce disease burden but also extend years of life in aging populations.

To fill these gaps, we calculated seven diet quality scores: the Alternate Healthy Eating Index (AHEI), Dietary Approaches to Stop Hypertension Trial (DASH) score, Mediterranean Diet (MED) score, Dietary Inflammatory Index (DII), and three plant-based diet indices (PDI), including overall PDI, healthful PDI (HPDI), and unhealthful PDI (UPDI). Our objective was to examine and compare the joint effects of these diet quality scores and frailty on all-cause mortality and life expectancy in middle-aged and older adults. This investigation could potentially guide public health interventions aimed at enhancing the well-being and longevity of the aging population.

## 2. Methods

### 2.1. Study Population

The UK Biobank recruited over 500,000 individuals aged between 40 and 69 years from the general population (5.5% response rate) across England, Wales, and Scotland during the period from 2006 to 2010 [18]. Detailed information on study design, implementation, and data acquisition has been reported elsewhere [19]. Briefly, the study design involved a large-scale prospective cohort approach, implementation was conducted through systematic recruitment via 22 assessment centers across the country, and data acquisition included participants completing a touchscreen questionnaire addressing various lifestyle and health-related factors, a face-to-face interview with a clinical nurse, a series of physical examinations, and provision of biological samples. The present study was conducted using the UK Biobank Resource, approved by the North West Multi-Center Research Ethics Committee (REC reference 21/NW/0157). All participants provided written informed consent. This research utilized the UK Biobank Resource under Application Number 63424 and 88159.

In the study involving 203,101 individuals with at least one dietary assessment, we initially excluded 33,416 participants due to incomplete data essential for creating the frailty index and frailty phenotype. Additionally, 26 participants were removed from the analysis owing to the absence of all-cause mortality data. Focusing our research on middle-aged and elderly adults, we further excluded 18,031 individuals who were under 45 years old at the time of recruitment. Consequently, 151,628 participants were included in the main analysis. The selection process is presented in the flowchart (Supplementary Figure S1).

### 2.2. Dietary Assessment

Dietary data on 206 foods and 32 beverages consumed in the past 24 h were collected using the Oxford WebQ, a validated 24 h dietary recall tool used in large-scale cohort studies [20,21]. The WebQ automatically generated estimated energy and nutrient values for foods and beverages based on participants’ reported consumption in the preceding 24 h, which has been validated with good reliability against both interviewer-administered 24 h recalls [20] and biomarkers [22]. The first instance of the WebQ was collected in the assessment centers from April 2009 to September 2010, with up to four additional occasions (cycle 1: February 2011 to April 2011; cycle 2: June 2011 to September 2011; cycle 3: October 2011 to December 2011; cycle 4: April 2012 to June 2012). People with unfeasible energy intake were excluded based on Henry’s equation (<800 or >4200 kcal/day in males and <600 or >3500 kcal/day in females) [23]. For those who completed twice and more, the intake of every food item was calculated as the mean of intake answered in all diet assessments.

We assessed diet quality using seven indices, including the Alternate Healthy Eating Index (AHEI), DASH score, Mediterranean diet score (MED), Dietary Inflammatory Index (DII), and Plant-based Diet Index (PDI, HPDI, UPDI). Higher scores on the AHEI, DASH, MED, PDI, and HPDI indicate adherence to healthier diets, while higher scores on the DII and UPDI represent adherence to unhealthier diets. Detailed descriptions of each index and their scoring criteria are provided in the Supplementary Method S2 and Supplementary Tables S3–S7.

### 2.3. Frailty

We assessed frailty using two methods: the frailty phenotype (FP) and the frailty index (FI). FP was evaluated using five criteria (weight loss, exhaustion, weakness, slow gait speed, and low physical activity), previously validated in the UK Biobank [24]. Participants were classified as frail (three or more criteria), prefrail (one or two criteria), or robust (zero criteria). The FI consisted of 49 clinical conditions and diseases, also called ‘deficits’, which has previously been reported elsewhere [24]. The FI was calculated by dividing the number of present deficits by the total possible, resulting in values from 0 to 1, with higher values indicating greater frailty. Participants were categorized as robust (FI ≤ 0.12), prefrail (FI 0.12–0.24), or frail (FI ≥ 0.24). Detailed definitions for FP and FI are in Supplementary Tables S1 and S2.

### 2.4. Covariates

Information on sociodemographic, lifestyle, and medical factors was obtained through a questionnaire at recruitment. Age at baseline (continuous) was calculated from the date of birth and the date of recruitment. Body mass index (BMI) was calculated as weight in kilograms divided by height in meters squared (<25.0, 25.0–29.9, ≥30 kg/m^2^). Data for sex (female or male), ethnicity (white or others), assessment center (England, Wales, Scotland), education level (college or university degree, secondary school, primary school, professional qualification), employment status (employed, retired, inactive), and household income (<18,000, 18,000–30,999, 31,000–51,999, 52,000–100,000, >£100,000 £/year) were collected. The Townsend deprivation index (quartile) was defined based on the postcode of residence using aggregated data on unemployment, car and home ownership, and household over-crowding, with higher scores indicating greater levels of socioeconomic deprivation [25]. Smoking status was categorized as “current”, “former”, or “never”; frequency of alcohol intake was measured as “≥3 times/week”, “<3 times/week”, or “never”; sleep duration was classified as “<7 h per day”, “7–8 h per day”, “>8 h per day”. The Metabolic Equivalent Task (MET) minutes, derived from items in the short International Physical Activity Questionnaire (IPAQ), were used to categorize physical activity as “high,” “moderate,” or “low” [26]. Family history of diabetes, cancer, and cardiovascular disease (CVD) was classified as yes or no. Total energy intake (continuous, in kcal/day) was calculated from the WebQ concurrently with diet quality scores.

### 2.5. Ascertainment of Mortality and Life Expectancy

The primary outcome of this study was all-cause mortality. Follow-up time was calculated in person-months from recruitment to death, loss to follow-up, or the end of the study, whichever came first. Mortality data were available up to 30 September 2021 for England and Wales and 31 October 2021 for Scotland, obtained through the National Health Service (NHS) Information Centre for participants in England and Wales and the NHS Central Register Scotland for those in Scotland.

Life expectancy was calculated using life tables [8,27], starting at age 50 and ending at 100, incorporating: (i) age- and sex-specific population mortality rates from the Office for National Statistics [28]; (ii) sex-specific hazard ratios (HRs) from combinations of diet quality and frailty; and (iii) sex-specific prevalence of each level of interaction. Multivariable-adjusted Cox regression models were fitted separately for each gender to calculate age-specific HRs for all-cause mortality. Life expectancy was estimated at any given age for both the reference group and each exposure group. Details of the methods used for estimating life expectancy have been described in Supplementary Method S1.

### 2.6. Statistical Analysis

Descriptive characteristics by frailty status are presented as frequencies with percentages for categorical variables and means with standard deviations (SD) for continuous variables. Missing data of covariates were inputted via R package ‘missForest’ [29].

Cox proportional hazard regression models were employed to assess the hazard ratios (HRs) and corresponding 95% confidence intervals (CIs) for the combined effects of diet quality scores and frailty status on all-cause mortality, using follow-up time as the underlying timescale. The proportional hazards assumption was examined through statistical tests based on scaled Schoenfeld residuals; age and assessment center were in violation of the assumption and corrected through stratifying. We adjusted for age at recruitment (strata), sex, assessment center (strata), body mass index, ethnicity, education, employment, household income, Townsend deprivation index, smoking status, alcohol drinking frequency, sleep duration, physical activity, energy, family history of diabetes, family history of CVD, and family history of cancer. For independent analysis, a *p*-value for trend was calculated to assess linear trends across ordered groups. Model fit was assessed using Akaike Information Criterion (AIC) and Bayesian Information Criterion (BIC).

To examine the joint effects of diet quality scores and frailty status on total mortality, three types of analyses were performed. First, we analyzed both the additive and multiplicative interactions between diet quality scores and frailty status with all-cause mortality. Second, according to different levels of interactive combinations of diet quality scores and frailty status, the overall sample was stratified into nine mutually exclusive groups. Therefore, the combined effects were investigated, adjusting all possible covariates. We evaluated interactions using likelihood ratio tests comparing models with and without a cross-product term. Third, we assessed the dose–response relationship between each diet score and all-cause mortality stratified by frailty status, as developed by either the frailty index or frailty phenotype. To analyze the shape of these associations, we applied restricted cubic splines (RCS) with four knots located at the 5th, 35th, 65th, and 95th percentiles of each exposure.

A series of sensitivity analyses were conducted to enhance the robustness of our findings. Firstly, to avoid potential overadjustment bias due to overlapping components in diet quality scores and covariates, the alcohol-related item was removed from both AHEI and MED, and items related to alcohol and energy were removed from DII, after which all analyses were rerun. Secondly, to reduce the risk of reverse causation, participants who died within the first two years of follow-up were excluded. Thirdly, to address the issue of missing data, we excluded participants with missing values for any of the covariates and also performed multiple imputation by chained equations (MICE) to replace missForest imputation. Fourthly, we additionally adjusted for medication use, including blood-pressure-lowering, cholesterol-lowering, and insulin medications. Fifthly, we additionally adjusted for overall health status. Finally, we examined the cumulative risk of all-cause mortality by diet quality scores and by frailty status.

All statistical analyses were conducted using R software version 4.3.1. We used Monte Carlo simulation (parametric bootstrapping) with 10,000 runs to calculate the CIs of the life expectancy estimation with boot R package. All statistical tests were two-sided, and we considered a *p* value of less than 0.05 to be statistically significant. As this was an exploratory analysis, *p*-values were interpreted without formal multiplicity correction.

## 3. Results

### 3.1. Baseline Characteristics

Table 1 presents the baseline characteristics of participants stratified by frailty index and frailty phenotype. Over a median follow-up period of 12.2 years, out of 151,628 individuals, 8231 individuals died. Additionally, the baseline characteristics of participants by each diet quality score are presented in Supplementary Tables S8–S14.

### 3.2. Joint Analysis Between Diet Quality Scores, Frailty, and All-Cause Mortality

**Figure 1:**
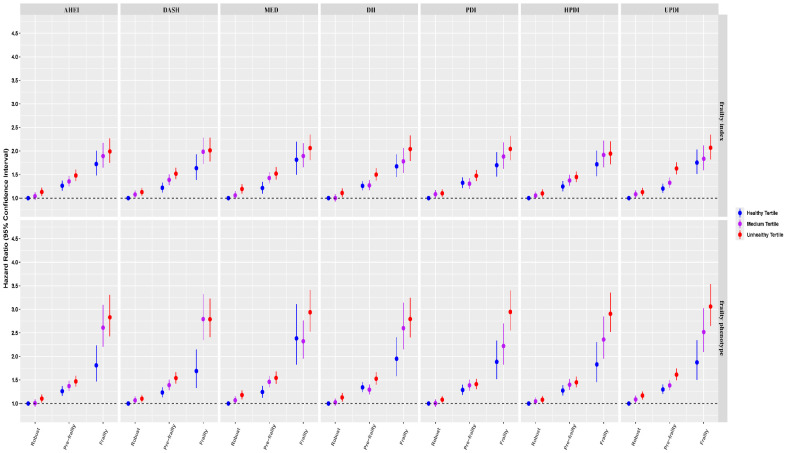
Joint associations of frailty status and diet quality scores with all-cause mortality. Diet quality scores were categorized into healthy (AHEI, DASH, MED, PDI, HPDI) and unhealthy (DII, UPDI) diets. The healthy tertile was defined as the top tertile of the healthy diet or the lowest tertile of the unhealthy diet, while the unhealthy tertile was defined as the lowest tertile of the healthy diet or the top tertile of the unhealthy diet. The model was adjusted for age at recruitment (strata), sex, assessment center (strata), body mass index, ethnicity, education, employment, household income, Townsend deprivation index, smoking status, alcohol drinking frequency, physical activity, energy, sleep duration, family history of diabetes, family history of CVD, and family history of cancer. AHEI, Alternative Healthy Eating Index; DASH, Dietary Approaches to Stop Hypertension; MED, Mediterranean Diet; DII, Dietary Inflammatory Index; PDI, Plant-Based Diet Index; HPDI, Healthy Plant-Based Diet Index; UPDI, Unhealthy Plant-Based Diet Index; CVD, cardiovascular disease.

The results of joint analyses involving diet quality scores and frailty status in relation to all-cause mortality, which revealed significant interaction effects (*p* for interaction < 0.001), are presented in Figure 1. There were risk gradients associated with increasing levels of frailty and decreasing scores for healthy diets, as well as increasing scores for unhealthy diets. After adjusting for all covariates, the highest mortality risk was observed among frail individuals in the unhealthier tertile of diet scores, compared to the non-frail reference group in the healthier tertile of diet scores. Notably, these diet–mortality associations showed consistent incremental patterns from the healthy to medium tertile and from the medium to unhealthy tertile across all diet indices, with the magnitude being particularly pronounced among frail individuals. These patterns were more evident for the frailty phenotype than the frailty index.

**Figure 2:**
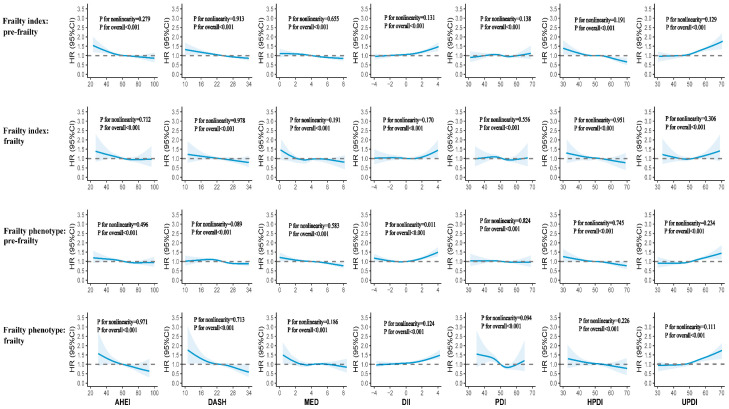
Dose–response associations (HR and 95%CI) between diet quality scores and all-cause mortality by frailty status using restricted cubic splines with four knots located at the 5th, 35th, 65th, and 95th percentiles of each exposure. The solid line represents the estimated association, and the shaded area indicates the 95% confidence interval. Diet quality scores were categorized as healthy (AHEI, DASH, MED, PDI, HPDI) and unhealthy (DII, UPDI). The models adjusted for age at recruitment (strata), sex, assessment center (strata), body mass index, and ethnicity, education, employment, household income, Townsend deprivation index, smoking status, alcohol drinking frequency, sleep duration, physical activity, energy, family history of diabetes, family history of CVD, and family history of cancer. AHEI, Alternative Healthy Eating Index; DASH, Dietary Approaches to Stop Hypertension; MED, Mediterranean Diet; DII, Dietary Inflammatory Index; PDI, Plant-Based Diet Index; HPDI, Healthy Plant-Based Diet Index; UPDI, Unhealthy Plant-Based Diet Index; CVD, cardiovascular disease; HR, hazard ratios; CI, confidence interval.

The results of stratification analysis investigating the dose–response relationship between diet quality scores and all-cause mortality by frailty status are presented in Figure 2. After adjusting for all covariates, although there were slight variations among different frailty categories by either frailty index or frailty phenotype, we observed an overall consistent shape for all-cause mortality: as levels of AHEI, DASH, MED, and HPDI increased, there was a progressive decrease in HRs for all-cause mortality, whereas an increase in DII and UPDI values was associated with a higher mortality risk. However, for PDI, the dose–response relationships were less clear.

### 3.3. Life Expectancy

**Figure 3:**
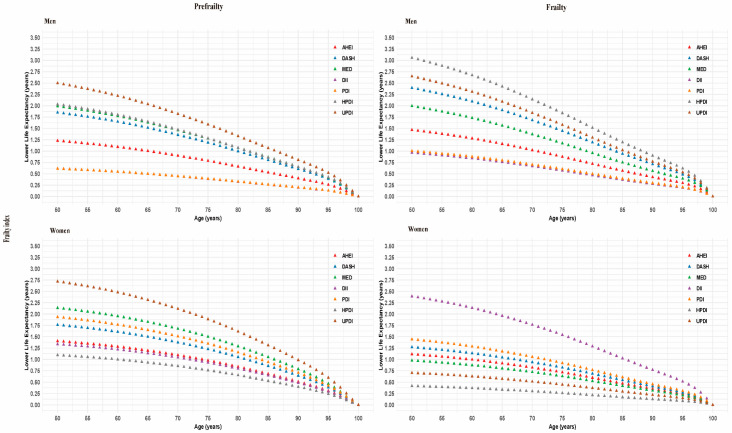
Gender-specific analysis of diet quality scores and life expectancy disparity by frailty index status. Diet quality scores were categorized as healthy (AHEI, DASH, MED, PDI, HPDI) and unhealthy (DII, UPDI). The healthy tertile was defined as the top tertile of the healthy diet or the lowest tertile of the unhealthy diet, while the unhealthy tertile was defined as the lowest tertile of the healthy diet or the top tertile of the unhealthy diet. Lower life expectancy was calculated by comparing the estimated life expectancy of individuals in the healthy tertile to those in the unhealthy tertile. The model adjusted for age at recruitment, sex, assessment center, body mass index, and ethnicity, education, employment, household income, Townsend deprivation index, smoking status, alcohol drinking frequency, sleep duration, physical activity, energy, family history of diabetes, family history of CVD, and family history of cancer. AHEI, Alternative Healthy Eating Index; DASH, Dietary Approaches to Stop Hypertension; MED, Mediterranean Diet; DII, Dietary Inflammatory Index; PDI, Plant-Based Diet Index; HPDI, Healthy Plant-Based Diet Index; UPDI, Unhealthy Plant-Based Diet Index; CVD, cardiovascular disease.

Differences in estimated life expectancy from 50 years of age onward in men and women by diet quality scores across frailty index status are presented in Figure 3. Compared with the healthier tertile of diet scores, life expectancy was significantly reduced in the unhealthier tertile, regardless of sex, a trend that was consistent in prefrailty and frailty status. At age 50, in frailty status, males in the unhealthier tertile experienced life expectancy reductions ranging from 0.9 to 3.1 years across various diet scores, while the corresponding reductions for females ranged from 0.5 to 2.4 years, all of which were statistically significant.

**Figure 4:**
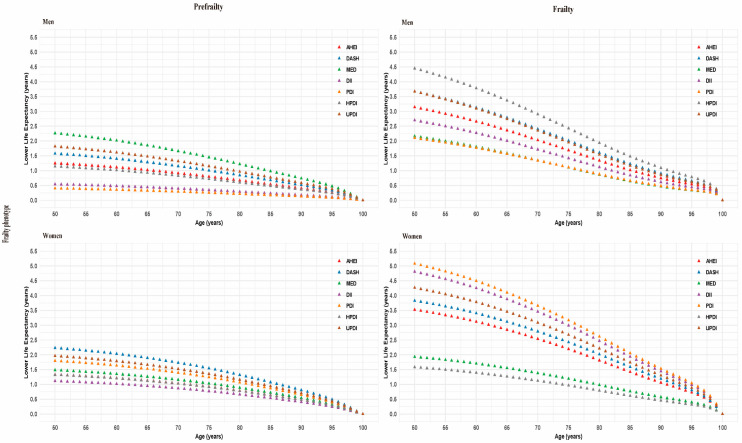
Gender-specific analysis of diet quality scores and life expectancy disparity by frailty phenotype status. Diet quality scores were categorized as healthy (AHEI, DASH, MED, PDI, HPDI) and unhealthy (DII, UPDI). The healthy tertile was defined as the top tertile of the healthy diet or the lowest tertile of the unhealthy diet, while the unhealthy tertile was defined as the lowest tertile of the healthy diet or the top tertile of the unhealthy diet. Lower life expectancy was calculated by comparing the estimated life expectancy of individuals in the healthy tertile to those in the unhealthy tertile. The model adjusted for age at recruitment, sex, assessment center, body mass index, and ethnicity, education, employment, household income, Townsend deprivation index, smoking status, alcohol drinking frequency, sleep duration, physical activity, energy, family history of diabetes, family history of CVD, and family history of cancer. AHEI, Alternative Healthy Eating Index; DASH, Dietary Approaches to Stop Hypertension; MED, Mediterranean Diet; DII, Dietary Inflammatory Index; PDI, Plant-Based Diet Index; HPDI, Healthy Plant-Based Diet Index; UPDI, Unhealthy Plant-Based Diet Index; CVD, cardiovascular disease.

Similarly, Figure 4 illustrates the life expectancy differences from 50 years of age onward in men and women by diet quality scores across frailty phenotype status. Analogous to the patterns observed in the frailty index, life expectancy decreased consistently for both males and females. At age 50, among those with frailty status, males in the unhealthier tertile experienced life expectancy reductions ranging from 2.1 to 4.5 years across various diet scores, while the corresponding reductions for females ranged from 1.6 to 5.1 years, all of which were statistically significant.

### 3.4. Sensitivity Analysis

When we removed the alcohol-related item from both AHEI and MED, and items related to alcohol and energy from DII, we found consistent results, and the magnitude of these effects showed slight variations (Supplementary Figures S2–S5). Independent analysis of diet quality scores and frailty on all-cause mortality is presented in Supplementary Tables S17 and S18. In addition, after the exclusion of events occurring within the first two years of follow-up, there were no substantial changes to the estimates (Supplementary Figure S6). Excluding all missing values for covariates did not markedly influence any estimates (Supplementary Figure S7). Results were consistent when using the MICE imputation method (Supplement Figure S8) when further adjusting for medication use (Supplementary Figure S9) and when further adjusting for overall health status (Supplementary Figure S10). Cumulative risk of all-cause mortality by diet quality scores and by frailty status is presented in Supplementary Figures S11 and S12.

## 4. Discussion

In this large cohort study, we found that diet quality significantly interacted with frailty, affecting all-cause mortality and life expectancy in middle-aged and older adults. The higher tertile of healthy diets, such as AHEI, DASH, MED, PDI and HPDI, was associated with a reduction in the adverse effects of frailty, while greater intake of unhealthy diets, like DII and UPDI, exacerbated these effects. These patterns were consistent across both genders, regardless of the method of frailty assessment.

Our study significantly contributes to this underexplored field by comparing a range of diet quality scores and their interaction with frailty on mortality, adjusting for various demographic, socioeconomic, lifestyle, and medical factors. The findings revealed that the healthier tertile of diet scores similarly mitigated frailty’s impact on mortality, while unhealthier tertiles similarly exacerbated frailty’s risks. This parallel pattern across different diet scores is noteworthy, as these indices were developed with different nutrients and may weigh various foods. Such observed consistency likely reflects the fundamental role of core dietary components shared across these indices, such as plant-based foods, antioxidants, fiber, and unsaturated fats, which are known to influence key physiological pathways including oxidative stress, inflammation, and metabolic regulation [15,16,30]. The remarkable concordance of our findings across multiple dietary indices provides compelling evidence for benefits of high quality of diet in reducing mortality risk, even among frail individuals. The consistency of our results across both frailty index and frailty phenotype models strengthens the reliability and broad applicability of our findings.

Frailty is a complex and multidimensional syndrome, involving declines across various physiological and functional domains. Different frailty measures capture distinct aspects of this syndrome, and no single operational definition fully encompasses its complexity. In our study, interactions between diet quality and frailty were generally stronger when frailty was defined using the FP compared to the FI. This may be partly because FP emphasizes physical function components such as grip strength and walking pace, which are more directly affected by nutritional status and physical resilience. In contrast, FI encompasses a broader spectrum of health deficits, including chronic diseases, symptoms, and disabilities, which may dilute the specificity of the diet–frailty interaction.

The findings of this research further highlight that the benefits of better diet quality in reducing mortality risk and extending life expectancy were evident across the entire spectrum of frailty, from robust individuals to those with prefrailty and frailty. This universality of benefit provides important insights into how dietary factors continue to influence health outcomes even as physiological reserve varies. Such findings are particularly significant given the growing recognition of frailty as a dynamic, potentially reversible condition rather than an irreversible state [31]. Importantly, the research underscores that it is never too late to adopt healthier dietary habits, as improvements in diet quality can yield meaningful health benefits regardless of one’s current frailty status or age. Meanwhile, it should be acknowledged that the relationship between diet and frailty may be bidirectional: while healthier diets can reduce the risk or progression of frailty, individuals with frailty may also modify their dietary patterns due to reduced appetite, functional limitations, or comorbid conditions [32]. This bidirectional interplay creates a complex feedback loop where frailty-related changes impact diet quality, and diet in turn influences frailty progression. This dynamic interplay underscores that effective interventions must combine nutritional improvements with strategies to address frailty-related challenges, as either alone may be insufficient. These insights justify the translation of our findings into integrated clinical and public health strategies. Routine frailty screening coupled with diet counseling could identify at-risk individuals early and provide personalized nutritional support. Multidisciplinary approaches incorporating physical rehabilitation, social support, and nutritional guidance may be more effective in breaking the frailty–diet cycle. Additionally, community-based programs aimed at improving food accessibility and education for older adults can mitigate social determinants impacting diet quality. Taken together, diet quality represents a cost-effective and broadly applicable lever to promote healthy aging and reduce mortality. Embedding nutritional strategies within frailty screening, clinical care, and community programs could yield substantial population-level benefits, particularly in the context of global population aging.

Remarkably, we found that the associations between dietary quality and mortality, as well as life expectancy, were more pronounced in frail individuals compared to robust individuals. These amplified effects might be attributed to the reduced physiological reserve and compromised multisystem function characteristic of frail individuals [33,34], which makes them especially sensitive to dietary influences. This phenomenon can be explained by several interrelated mechanisms. One major factor is the synergistic effects on inflammation and oxidative stress. Frail individuals typically exhibit elevated levels of inflammatory cytokines such as IL-6 and CRP, markers of systemic inflammation [35]. A pro-inflammatory diet further exacerbates this inflammatory state, amplifying chronic inflammation, which accelerates immunosenescence [36]. Additionally, the lack of antioxidants in unhealthy diets fails to counterbalance oxidative stress, creating a vicious cycle of inflammation and oxidative damage that worsens health outcomes and increases mortality risk [37]. In contrast, robust individuals have stronger physiological reserves to mitigate these inflammatory and oxidative responses. Additionally, frail individuals often have impaired glucose regulation and insulin resistance, which are aggravated by diets high in saturated fats and refined carbohydrates. This metabolic dysregulation is more detrimental in frail individuals due to their limited ability to adapt, accelerating cardiometabolic complications [38]. Robust individuals, however, are better equipped to maintain glucose homeostasis due to greater metabolic flexibility. Finally, compared with healthy counterparts, frail individuals are more susceptible to nutrient deficiencies due to reduced food intake and impaired absorption, weakening immune function and increasing infection risk [39], which in turn substantially elevates mortality risks.

This study has several notable strengths. It leverages a large-scale cohort with a prospective design and long follow-up, providing substantial statistical power for mortality outcomes. Seven diet quality scores were systematically evaluated within a single analytic framework, enabling direct comparisons. Frailty was operationalized using two complementary methods, enhancing construct validity. Our analyses incorporated extensive adjustment for sociodemographic, lifestyle, and clinical covariates, collected by a touchscreen questionnaire and verified in nurse-led face-to-face interviews, thereby improving covariate ascertainment and limiting potential residual confounding. Finally, we extended beyond traditional mortality risk to estimate life expectancy, yielding clinically interpretable and policy-relevant metrics.

This study has several limitations that warrant consideration. The reliance on the UK Biobank’s web-based 24 h dietary assessment tool, despite its validation against biomarkers [22], introduces potential measurement errors inherent to self-reported data. Such measurement errors are more likely to attenuate the observed associations. We used data from up to five rounds of 24 h dietary recall records in the UK Biobank to reduce the possibility of these errors. Nevertheless, it is important to recognize that dietary intake may vary over the long follow-up period, a factor not captured in this study, potentially leading to regression dilution bias and underestimation of true diet–mortality associations, which suggests that our results are likely conservative. Although implausible energy intake values were excluded using widely adopted thresholds (<800 or >4200 kcal/day for men; <600 or >3500 kcal/day for women), some degree of energy misreporting is still possible. Future studies could apply more individualized approaches, such as the Goldberg method, to further improve accuracy in identifying misreporters. While the frailty measures in this study followed established and widely adopted methodologies, they primarily relied on self-reported data (e.g., gait speed), which may introduce potential misclassification bias. Although we have controlled for the majority of confounders that were considered relevant, the possibility of residual confounding cannot be ruled out. Given the nature of this observational study, we were not able to determine causality. Reverse causation could be possible; however, results were similar after excluding deaths during the first two years of follow-up, thus reducing the opportunity for reverse causation. Finally, the UK Biobank is not representative of the UK population in terms of lifestyle and disease prevalence and primarily includes middle-aged and older adults; thus, generalizing findings should be done with caution. Its low baseline response rate (~5.5%) may introduce selection bias. Nevertheless, previous studies have shown that internal associations between exposures and health outcomes remain robust [40].

## 5. Conclusions

In conclusion, this study highlights the significant interaction between diet quality scores and frailty in determining all-cause mortality and life expectancy among middle-aged and older adults. Our findings emphasize the importance of considering diet quality in public health strategies to manage frailty, enhance longevity, and reduce mortality risks. Continued research, including mechanistic studies, randomized controlled trials, and population-based intervention studies, is vital to unravel the pathways by which dietary patterns influence frailty progression and to guide the development of effective dietary recommendations for aging populations. Additionally, large-scale cohort studies in diverse populations are encouraged to replicate and validate our findings across different demographic and cultural contexts.

## Figures and Tables

**Table 1:** Baseline characteristics of the participants by frailty status.

	Total	Frailty Index			Frailty Phenotype		
		Robust	Prefrailty	Frailty	Robust	Prefrailty	Frailty
	(N = 151,628)	(N = 93,414)	(N = 51,393)	(N = 6821)	(N = 96,697)	(N = 51,849)	(N = 3082)
Age at recruitment, years, mean (SD)	57.8 (6.7)	57.3 (6.7)	58.4 (6.6)	58.8 (6.5)	57.6 (6.7)	58.0 (6.7)	58.5 (6.5)
Sex, n (%)							
female	81,886 (54.0%)	49,032 (52.5%)	28,841 (56.1%)	4013 (58.8%)	50,401 (52.1%)	29,506 (56.9%)	1979 (64.2%)
male	69,742 (46.0%)	44,382 (47.5%)	22,552 (43.9%)	2808 (41.2%)	46,296 (47.9%)	22,343 (43.1%)	1103 (35.8%)
Body mass index (BMI), kg/m^2^, n (%)							
<25.0	56,271 (37.1%)	39,051 (41.8%)	16,003 (31.1%)	1217 (17.8%)	40,612 (42.0%)	15,183 (29.3%)	476 (15.4%)
25.0–29.9	64,154 (42.3%)	39,836 (42.6%)	21,767 (42.4%)	2551 (37.4%)	41,309 (42.7%)	21,901 (42.2%)	944 (30.6%)
≥30.0	31,203 (20.6%)	14,527 (15.6%)	13,623 (26.5%)	3053 (44.8%)	14,776 (15.3%)	14,765 (28.5%)	1662 (53.9%)
Ethnicity, n (%)							
white	146,918 (96.9%)	90,690 (97.1%)	49,688 (96.7%)	6540 (95.9%)	94,381 (97.6%)	49,660 (95.8%)	2877 (93.3%)
other	4710 (3.1%)	2724 (2.9%)	1705 (3.3%)	281 (4.1%)	2316 (2.4%)	2189 (4.2%)	205 (6.7%)
Education, n (%)							
college or university degree	65,108 (42.9%)	43,283 (46.3%)	19,891 (38.7%)	1934 (28.4%)	44,134 (45.6%)	20,205 (39.0%)	769 (25.0%)
secondary school	56,596 (37.3%)	34,051 (36.5%)	19,833 (38.6%)	2712 (39.8%)	35,202 (36.4%)	20,110 (38.8%)	1284 (41.7%)
primary school	13,327 (8.8%)	6428 (6.9%)	5643 (11.0%)	1256 (18.4%)	7065 (7.3%)	5616 (10.8%)	646 (21.0%)
professional qualification	16,597 (10.9%)	9652 (10.3%)	6026 (11.7%)	919 (13.5%)	10,296 (10.6%)	5918 (11.4%)	383 (12.4%)
Employment, n (%)							
employed	85,250 (56.2%)	55,911 (59.9%)	26,727 (52.0%)	2612 (38.3%)	56,014 (57.9%)	28,116 (54.2%)	1120 (36.3%)
other	66,378 (43.8%)	37,503 (40.1%)	24,666 (48.0%)	4209 (61.7%)	40,683 (42.1%)	23,733 (45.8%)	1962 (63.7%)
Household income, £/year, n (%)							
less than 18,000	25,890 (17.1%)	12,250 (13.1%)	10,983 (21.4%)	2657 (39.0%)	13,772 (14.2%)	10,794 (20.8%)	1324 (43.0%)
18,000 to 30,999	37,849 (25.0%)	22,145 (23.7%)	13,866 (27.0%)	1838 (26.9%)	23,369 (24.2%)	13,685 (26.4%)	795 (25.8%)
31,000 to 51,999	41,422 (27.3%)	26,365 (28.2%)	13,665 (26.6%)	1392 (20.4%)	27,044 (28.0%)	13,813 (26.6%)	565 (18.3%)
52,000 to 100,000	34,892 (23.0%)	24,027 (25.7%)	10,114 (19.7%)	751 (11.0%)	24,160 (25.0%)	10,414 (20.1%)	318 (10.3%)
greater than 100,000	11,575 (7.6%)	8627 (9.2%)	2765 (5.4%)	183 (2.7%)	8352 (8.6%)	3143 (6.1%)	80 (2.6%)
Townsend deprivation index, n (%)							
first quartile	37,934 (25.0%)	24,624 (26.4%)	12,136 (23.6%)	1174 (17.2%)	25,850 (26.7%)	11,640 (22.4%)	444 (14.4%)
second quartile	37,882 (25.0%)	23,952 (25.6%)	12,513 (24.3%)	1417 (20.8%)	24,908 (25.8%)	12,392 (23.9%)	582 (18.9%)
third quartile	37,906 (25.0%)	23,354 (25.0%)	12,938 (25.2%)	1614 (23.7%)	24,039 (24.9%)	13,129 (25.3%)	738 (23.9%)
fourth quartile	37,906 (25.0%)	21,484 (23.0%)	13,806 (26.9%)	2616 (38.4%)	21,900 (22.6%)	14,688 (28.3%)	1318 (42.8%)
Smoking status, n (%)							
never	85,091 (56.1%)	55,644 (59.6%)	26,465 (51.5%)	2982 (43.7%)	55,658 (57.6%)	28,005 (54.0%)	1428 (46.3%)
previous	55,795 (36.8%)	31,898 (34.1%)	20,856 (40.6%)	3041 (44.6%)	34,918 (36.1%)	19,614 (37.8%)	1263 (41.0%)
current	10,742 (7.1%)	5872 (6.3%)	4072 (7.9%)	798 (11.7%)	6121 (6.3%)	4230 (8.2%)	391 (12.7%)
Alcohol drinking frequency, n (%)							
≥3 times/week	76,319 (50.3%)	49,856 (53.4%)	24,177 (47.0%)	2286 (33.5%)	52,772 (54.6%)	22,774 (43.9%)	773 (25.1%)
<3 times/week	66,389 (43.8%)	39,070 (41.8%)	23,687 (46.1%)	3632 (53.2%)	39,512 (40.9%)	25,119 (48.4%)	1758 (57.0%)
never	8920 (5.9%)	4488 (4.8%)	3529 (6.9%)	903 (13.2%)	4413 (4.6%)	3956 (7.6%)	551 (17.9%)
Sleep duration, n (%)							
7–8 h/day	107,921 (71.2%)	70,894 (75.9%)	33,670 (65.5%)	3357 (49.2%)	71,742 (74.2%)	34,715 (67.0%)	1464 (47.5%)
<7h/day	33,649 (22.2%)	17,298 (18.5%)	13,766 (26.8%)	2585 (37.9%)	19,394 (20.1%)	13,138 (25.3%)	1117 (36.2%)
>8h/day	10,058 (6.6%)	5222 (5.6%)	3957 (7.7%)	879 (12.9%)	5561 (5.8%)	3996 (7.7%)	501 (16.3%)
Physical activity, n (%)							
high	59,729 (39.4%)	38,445 (41.2%)	19,025 (37.0%)	2259 (33.1%)	39,854 (41.2%)	19,267 (37.2%)	608 (19.7%)
moderate	60,258 (39.7%)	37,284 (39.9%)	20,575 (40.0%)	2399 (35.2%)	39,446 (40.8%)	19,936 (38.5%)	876 (28.4%)
low	31,641 (20.9%)	17,685 (18.9%)	11,793 (22.9%)	2163 (31.7%)	17,397 (18.0%)	12,646 (24.4%)	1598 (51.8%)
Energy, kcal, mean (SD)	2030 (524)	2030 (516)	2040 (533)	2020 (567)	2050 (516)	2000 (534)	1920 (565)
AHEI, n (%)							
unhealthy tertile	50,543 (33.3%)	30,429 (32.6%)	17,609 (34.3%)	2505 (36.7%)	31,712 (32.8%)	17,646 (34.0%)	1185 (38.4%)
medium tertile	50,542 (33.3%)	31,287 (33.5%)	17,024 (33.1%)	2231 (32.7%)	32,293 (33.4%)	17,259 (33.3%)	990 (32.1%)
healthy tertile	50,543 (33.3%)	31,698 (33.9%)	16,760 (32.6%)	2085 (30.6%)	32,692 (33.8%)	16,944 (32.7%)	907 (29.4%)
DASH, n (%)							
unhealthy tertile	54,277 (35.8%)	32,168 (34.4%)	19,237 (37.4%)	2872 (42.1%)	33,482 (34.6%)	19,395 (37.4%)	1400 (45.4%)
medium tertile	49,349 (32.5%)	30,744 (32.9%)	16,447 (32.0%)	2158 (31.6%)	31,797 (32.9%)	16,614 (32.0%)	938 (30.4%)
healthy tertile	48,002 (31.7%)	30,502 (32.7%)	15,709 (30.6%)	1791 (26.3%)	31,418 (32.5%)	15,840 (30.6%)	744 (24.1%)
MED, n (%)							
unhealthy tertile	53,299 (35.2%)	31,433 (33.6%)	18,951 (36.9%)	2915 (42.7%)	32,250 (33.4%)	19,551 (37.7%)	1498 (48.6%)
medium tertile	64,070 (42.3%)	39,768 (42.6%)	21,509 (41.9%)	2793 (40.9%)	41,306 (42.7%)	21,614 (41.7%)	1150 (37.3%)
healthy tertile	34,259 (22.6%)	22,213 (23.8%)	10,933 (21.3%)	1113 (16.3%)	23,141 (23.9%)	10,684 (20.6%)	434 (14.1%)
DII, n (%)							
healthy tertile	50,543 (33.3%)	31,239 (33.4%)	17,201 (33.5%)	2103 (30.8%)	33,553 (34.7%)	16,242 (31.3%)	748 (24.3%)
medium tertile	50,542 (33.3%)	31,511 (33.7%)	16,987 (33.1%)	2044 (30.0%)	32,791 (33.9%)	16,912 (32.6%)	839 (27.2%)
unhealthy tertile	50,543 (33.3%)	30,664 (32.8%)	17,205 (33.5%)	2674 (39.2%)	30,353 (31.4%)	18,695 (36.1%)	1495 (48.5%)
PDI, n (%)							
unhealthy tertile	59,537 (39.3%)	36,406 (39.0%)	20,409 (39.7%)	2722 (39.9%)	37,074 (38.3%)	21,078 (40.7%)	1385 (44.9%)
medium tertile	45,210 (29.8%)	28,190 (30.2%)	15,028 (29.2%)	1992 (29.2%)	29,075 (30.1%)	15,272 (29.5%)	863 (28.0%)
healthy tertile	46,881 (30.9%)	28,818 (30.8%)	15,956 (31.0%)	2107 (30.9%)	30,548 (31.6%)	15,499 (29.9%)	834 (27.1%)
HPDI, n (%)							
unhealthy tertile	55,751 (36.8%)	33,120 (35.5%)	19,710 (38.4%)	2921 (42.8%)	34,791 (36.0%)	19,572 (37.7%)	1388 (45.0%)
medium tertile	46,252 (30.5%)	28,673 (30.7%)	15,595 (30.3%)	1984 (29.1%)	29,682 (30.7%)	15,670 (30.2%)	900 (29.2%)
healthy tertile	49,625 (32.7%)	31,621 (33.9%)	16,088 (31.3%)	1916 (28.1%)	32,224 (33.3%)	16,607 (32.0%)	794 (25.8%)
UPDI, n (%)							
healthy tertile	53,591 (35.3%)	33,718 (36.1%)	17,835 (34.7%)	2038 (29.9%)	35,286 (36.5%)	17,548 (33.8%)	757 (24.6%)
medium tertile	48,237 (31.8%)	29,984 (32.1%)	16,160 (31.4%)	2093 (30.7%)	31,033 (32.1%)	16,297 (31.4%)	907 (29.4%)
unhealthy tertile	49,800 (32.8%)	29,712 (31.8%)	17,398 (33.9%)	2690 (39.4%)	30,378 (31.4%)	18,004 (34.7%)	1418 (46.0%)
Family history of CVD, n (%)							
no	36,339 (24.0%)	24,673 (26.4%)	10,605 (20.6%)	1061 (15.6%)	23,944 (24.8%)	11,786 (22.7%)	609 (19.8%)
yes	115,289 (76.0%)	68,741 (73.6%)	40,788 (79.4%)	5760 (84.4%)	72,753 (75.2%)	40,063 (77.3%)	2473 (80.2%)
Family history of cancer, n (%)							
no	95,205 (62.8%)	59,425 (63.6%)	31,645 (61.6%)	4135 (60.6%)	60,942 (63.0%)	32,344 (62.4%)	1919 (62.3%)
yes	56,423 (37.2%)	33,989 (36.4%)	19,748 (38.4%)	2686 (39.4%)	35,755 (37.0%)	19,505 (37.6%)	1163 (37.7%)
Family history of diabetes, n (%)							
no	119,765 (79.0%)	75,375 (80.7%)	39,535 (76.9%)	4855 (71.2%)	77,684 (80.3%)	39,952 (77.1%)	2129 (69.1%)
yes	31,863 (21.0%)	18,039 (19.3%)	11,858 (23.1%)	1966 (28.8%)	19,013 (19.7%)	11,897 (22.9%)	953 (30.9%)

AHEI, Alternative Healthy Eating Index; DASH, Dietary Approaches to Stop Hypertension; MED, Mediterranean Diet; DII, Dietary Inflammatory Index; PDI, Plant-Based Diet Index; HPDI, Healthy Plant-Based Diet Index; UPDI, Unhealthy Plant-Based Diet Index; CVD, cardiovascular disease; SD: standard deviation. Employment status is categorized as employed (includes paid employment or self-employed, paid, or voluntary work, or student) and other (includes retired, looking after home and/or family, unable to work, and unemployed). Education is categorized as college or university degree, secondary school (includes A levels/AS levels or equivalent, O levels/GCSEs or equivalent, CSEs or equivalent), primary school, and professional qualification (NVQ or HND or HNC or equivalent, other professional qualifications).

## Data Availability

The data of this study can be requested from the UK Biobank (https://www.ukbiobank.ac.uk/ (accessed on 24 June 2020)).

## Supplementary Materials

The following supporting information can be downloaded at https://www.mdpi.com/article/10.3390/nu17193115/s1, Supplementary Method S1: Estimating the difference in life expectancy [1,28,41,42]; Supplementary Method S2: Dietary assessment [16,20,21,22,23,30,43,44,45,46,47,48,49,50,51,52,53]; Table S1: Frailty phenotype and criteria for scoring; Table S2: Frailty index and criteria for scoring; Table S3: Alternate Healthy Eating Index (AHEI) components and criteria for scoring; Table S4: Dietary Approaches to Stop Hypertension (DASH) components and criteria for scoring; Table S5: Mediterranean Diet Score (MED) components and criteria for scoring; Table S6: Dietary inflammatory index (DII) components and criteria for scoring; Table S7: Plant-based diet index (PDI) and its subtypes components and criteria for scoring; Table S8: Baseline characteristics of the participants by AHEI; Table S9: Baseline characteristics of the participants by DASH; Table S10: Baseline characteristics of the participants by MED; Table S11: Baseline characteristics of the participants by DII; Table S12: Baseline characteristics of the participants by PDI; Table S13: Baseline characteristics of the participants by HPDI; Table S14: Baseline characteristics of the participants by UPDI; Table S15: Analyses on interaction of diet quality scores and pre-frailty with all-cause mortality; Table S16: Analyses on interaction of diet quality scores and frailty with all-cause mortality; Table S17: Association between frailty status and all-cause mortality; Table S18: Association between diet quality scores and all-cause mortality; Figure S1: Flowchart of participant enrolment; Figure S2: Joint associations of frailty status and diet quality scores (modified) with all-cause mortality; Figure S3: Dose–response associations (HR and 95%CI) between diet quality scores (modified) with all-cause mortality by frailty status using restricted cubic splines with four knots located at the 5th, 35th, 65th, and 95th percentiles of each exposure; Figure S4: Gender-specific analysis of diet quality scores (modified) and life expectancy disparity by frailty index status; Figure S5: Gender-specific analysis of diet quality scores (modified) and life expectancy disparity by frailty phenotype status; Figure S6: Joint associations of frailty status and diet quality scores with all-cause mortality (remove death in the first 2 years); Figure S7: Joint associations of frailty status and diet quality scores with all-cause mortality (remove missing values); Figure S8: Joint associations of frailty status and diet quality scores with all-cause mortality (MICE imputation); Figure S9: Joint associations of frailty status and diet quality scores with all-cause mortality (further adjusted for medications); Figure S10: Joint associations of frailty status and diet quality scores with all-cause mortality (further adjusted for overall health status); Figure S11: Cumulative risk of all-cause mortality by diet quality scores; Figure S12: Cumulative risk of all-cause mortality by frailty status.
