# Supplementary material for: Combined Effects of Diet Quality Scores and Frailty on All-Cause Mortality and Life Expectancy in Middle-Aged and Older Adults
Source: Nutrients. 2025 Sep 30;17(19):3115. doi: 10.3390/nu17193115 (PMC12525783; doi:10.3390/nu17193115)

Supplementary

Supplementary Method S1. Estimating the difference in life expectancy
Supplementary Method S2. Dietary assessment
Supplementary Table S1. Frailty phenotype and criteria for scoring
Supplementary Table S2. Frailty index and criteria for scoring
Supplementary Table S3. Alternate Healthy Eating Index (AHEI) components and criteria for scoring
Supplementary Table S4. Dietary Approaches to Stop Hypertension (DASH) components and criteria for scoring
Supplementary Table S5. Mediterranean Diet Score (MED) components and criteria for scoring
Supplementary Table S6. Dietary inflammatory index (DII) components and criteria for scoring
Supplementary Table S7. Plant-based diet index (PDI) and its subtypes components and criteria for scoring
Supplementary Table S8. Baseline characteristics of the participants by AHEI
Supplementary Table S9. Baseline characteristics of the participants by DASH
Supplementary Table S10. Baseline characteristics of the participants by MED
Supplementary Table S11. Baseline characteristics of the participants by DII
Supplementary Table S12. Baseline characteristics of the participants by PDI
Supplementary Table S13. Baseline characteristics of the participants by HPDI
Supplementary Table S14. Baseline characteristics of the participants by UPDI
Supplementary Table S15. Analyses on interaction of diet quality scores and pre-frailty with all-cause mortality
Supplementary Table S16. Analyses on interaction of diet quality scores and frailty with all-cause mortality
Supplementary Table S17. Association between frailty status and all-cause mortality
Supplementary Table S18. Association between diet quality scores and all-cause mortality
Supplementary Figure S1. Flowchart of participant enrolment
Supplementary Figure S2. Joint associations of frailty status and diet quality scores (modified) with all-cause mortality
Supplementary Figure S3. Dose–response associations (HR and 95%CI) between diet quality scores (modified) with all-cause mortality by frailty status using restricted cubic splines with four knots located at the 5th, 35th, 65th, and 95th percentiles of each exposure
Supplementary Figure S4. Gender-specific analysis of diet quality scores (modified) and life expectancy disparity by frailty index status
Supplementary Figure S5. Gender-specific analysis of diet quality scores (modified) and life expectancy disparity by frailty phenotype status
Supplementary Figure S6. Joint associations of frailty status and diet quality scores with all-cause mortality (remove death in the first 2 years)
Supplementary Figure S7. Joint associations of frailty status and diet quality scores with all-cause mortality (remove missing values)
Supplementary Figure S8. Joint associations of frailty status and diet quality scores with all-cause mortality (MICE imputation)
Supplementary Figure S9. Joint associations of frailty status and diet quality scores with all-cause mortality (further adjusted for medications)

Supplementary Figure S10. Joint associations of frailty status and diet quality scores with all-cause mortality (further adjusted for overall health status)

Supplementary Figure S11. Cumulative risk of all-cause mortality by diet quality scores

Supplementary Figure S12. Cumulative risk of all-cause mortality by frailty status

Supplementary Method S1. Estimating the differences in life expectancy

We combined information from three sources within the same population to estimate lower survival time associated with different levels of interactive combinations of diet quality scores and frailty status. Diet quality was classified into three tertiles: healthy, medium, and unhealthy; frailty status was categorized as robust, prefrail, and frail. The reference group was individuals classified as robust with a healthy diet tertile.

(1) Sex- and age- specific population mortality rate from the Office for National Statistics [1];

(2) Sex-specific HRs of all-cause mortality in each exposure group versus the reference in UK biobank;

(2) Sex-specific prevalence of each frequency of different levels in UK biobank.

The sex-specific lifetables for 8 exposure groups in their combinations were built on the above-mentioned three estimates. Population all-cause mortality rates per 100,000 per sex and per single-year age group were obtained from the Office for National Statistics. We used sex-specific Cox regression models to calculate adjusted hazard ratios for all-cause mortality by exposure groups. We made adjustments for age at recruitment, sex, assessment center, body mass index, ethnicity, education, employment, household income, Townsend deprivation index, smoking status, alcohol drinking frequency, physical activity, energy, sleep duration, family history of diabetes, family history of CVD, and family history of cancer. Then we applied the sex- specific HRs to estimate the life expectancy at different age of women and men, separately.

We built the life table starting at age 50 years and ending at 100 years by single-year age intervals. Survival probability was set of 1 at age 50 years and probability of survival between ages x and x + 1 was calculated based on probability of dying (mortality rate) between ages x and x+1 assuming that survivor function declines linearly between ages x and x + 1 [2,3]. The life expectancy at any given age was derived by dividing the total person-years that would be lived beyond age x by the number of persons who survived to that age interval [2].

We inferred the age-specific mortality rates appropriate for our reference group IR_{a0} as [4]:

$$IR_{a0} = \frac{IR_a}{(P_{a0} + \sum_{j=1}^{n} P_{aj} \times HR_{aj})}$$

Where IRa is the population mortality rate for age group a, P_{aj} is the prevalence of exposure group j, and $HRaj$ is the hazard ratio in comparison of exposure group j versus reference group (j = 0). The age-specific mortality rates in each of the non-reference exposure groups were then inferred in turn by multiplying the age-specific mortality rate for the reference group $IRa0$ by the hazard ratios $HRaj$.

Finally, life table was built for each exposure group and the reference group.

References

1. Single-year life tables, UK:1980-2020.

Available:

https://www.ons.gov.uk/peoplepopulationandcommunity/birthsdeathsandmarriages/lifeexpectancies/datasets/singleyearlifetablesuk1980to2018/singleyearlifetablesuk [Accessed 12 Mar 2024].

2.Arias E. United States life tables, 2008. Natl Vital Stat Rep. 2012 Sep 24;61(3):1-63.

3.Chiang CL, World Health Organization. Life table and mortality analysis. 1979. Publisher: Geneva : World Health Organization.

4. Woloshin S, Schwartz LM, Welch HG. The risk of death by age, sex, and smoking status in the United States: putting health risks in context. J Natl Cancer Inst 2008;100(12):845-53.

Supplement Method S2. Dietary assessment

Dietary information on 206 foods and 32 beverages consumed during the past 24 hours was collected using the Oxford WebQ based on a 24-h dietary recall questionnaire, which has already been used by several large-scale cohort studies [1-2]. The Oxford WebQ automatically generated estimated energy and nutrient values for foods and beverages reported by participants, using data on the consumption of these items in the preceding 24 hours. The Oxford WebQ has been validated with good reliability against an interviewer-administered 24-h recall [1] and biomarkers [3]. The first instance of Oxford WebQ was collected in the assessment centers from April 2009 to September 2010, and on up to four separate occasions (cycle 1: February 2011 to April 2011; cycle 2: June 2011 to September 2011; cycle 3: October 2011 to December 2011; cycle 4: April 2012 to June 2012). People with unfeasible energy intake were excluded based on Henry's equation (< 800 or > 4200 kcal/day in males and < 600 or > 3500 kcal/day in females) [4]. For those who completed twice and more, the intake of every food item was calculated as the mean of intake answered in all diet assessments.

Alternate healthy eating index

The AHEI was created as an alternative to the Healthy Eating Index (HEI), aiming to provide a comprehensive assessment of diet quality associated with a reduced risk of major chronic diseases [5,6]. The AHEI was calculated based on 11 food/nutrient groups in our analysis, including whole grains, vegetables, fruits, nuts and legumes, sugar-sweetened beverages and fruit juice, red and processed meat, Trans fatty acid, Long-chain (n-3) fats, polyunsaturated fatty acid, sodium, and alcohol [5,6]. Each component was proportionately scored from 0 to 10 and the total AHEI score ranged from 0 to 110, with a higher score representing a healthier diet (Supplement Table 3).

Dietary Approaches to Stop Hypertension

The DASH score was designed to reduce blood pressure [7]. The calculation of the DASH score depended on 8 components, and each component was scored from 0 (unhealthy) to 5 (healthy) based on sex-specific quintiles of the selected population [8,9]. Components included vegetables, fruits, nuts and legumes, whole grains, low-fat dairy, red and processed meat, sugar-sweetened beverages, and sodium and thus total score ranged from 8 to 40, with a higher score representing a healthier diet (Supplement Table 4).

Mediterranean diet score

The MED score adherence to the traditional Mediterranean diet, recognized for its capacity to alleviate adverse health outcomes in numerous epidemiological studies [10,11]. The MED was determined using 9 food/nutrient items including vegetables, fruits

and nuts, legumes, whole grains, fish, the ratio of monounsaturated fatty acid to saturated fatty acid, red and processed meat, dairy products, and alcohol [10,12]. The sex-specific median intakes of included participants were used to assign a score of 0 (unhealthy) or 1 (healthy) to each food item. Therefore, the total score ranged from 0 to 9, with a higher score representing a healthier diet (Supplement Table 5).

Dietary inflammatory index

The DII was a literature and population-based tool that can assess dietary inflammation [13]. Following the DII scoring algorithm proposed by Shivappa et al. in 2014 [13], 28 foods and nutrients available in the UK Biobank dataset were included to create the DII: Vitamin B12, Vitamin B6, β-carotene, carbohydrate, cholesterol, total fat, fiber, folic acid, Fe, Mg, monounsaturated fatty acids, Niacin, n-3 fatty acids, n-6 fatty acids, protein, Riboflavin, saturated fat, Se, Thiamin, trans fat, Vitamin A, Vitamin C, Vitamin D, Vitamin E, Zn, polyunsaturated fatty acids. Detailed calculation process has been described elsewhere [14]. The overall DII score, ranged from -5.27 to 5.77, with the higher score representing a higher pro-inflammatory diet (Supplement Table 6).

Plant-based diet index

We calculated the PDI, HPDI, and UPDI using established methods to assess the adherence to overall, healthful, and unhealthful plant-based diets, respectively [15,16]. Three indexes were computed based on 17 food or nutrition items and further classified as healthy plant-based food (vegetables, fruits, nuts, legumes, whole grains, tea and/or coffee), unhealthy plant-based food (potatoes, refined grains, sugar-sweetened beverages, fruit juice, sweets and desserts), and animal-based food (animal fat, dairy, eggs, fish/seafood, meat, miscellaneous animal-based foods) [17]. Then, a positive score or reverse score ranging from 1 to 5 would be distributed to each item based on the quintiles of intake (Supplement Table 7). The final scores ranged from 27 to 76 for PDI, from 26 to 77 for HPDI, and from 25 to 75 for UPDI, respectively.

Reference

1. Liu B, Young H, Crowe FL, Benson VS, Spencer EA, Key TJ, Appleby PN, Beral V. Development and evaluation of the Oxford WebQ, a low-cost, web-based method for assessment of previous 24 h dietary intakes in large-scale prospective studies. Public Health Nutr. 2011 Nov;14(11):1998-2005. doi: 10.1017/S1368980011000942. Epub 2011 Jun 1. PMID: 21729481.
2. Fry A, Littlejohns TJ, Sudlow C, Doherty N, Adamska L, Sprosen T, Collins R, Allen NE. Comparison of Sociodemographic and Health-Related Characteristics of UK Biobank Participants With Those of the General Population. Am J Epidemiol. 2017 Nov 1;186(9):1026-1034. doi: 10.1093/aje/kwx246. PMID: 28641372; PMCID: PMC5860371.
3. Greenwood DC, Hardie LJ, Frost GS, Alwan NA, Bradbury KE, Carter M, Elliott P, Evans CEL, Ford HE, Hancock N, Key TJ, Liu B, Morris MA, Mulla UZ, Petropoulou K, Potter GDM, Riboli E, Young H, Wark PA, Cade JE. Validation of the Oxford WebQ Online 24-Hour Dietary Questionnaire Using Biomarkers. Am J Epidemiol. 2019 Oct 1;188(10):1858-1867. doi: 10.1093/aje/kwz165. PMID: 31318012; PMCID: PMC7254925.
4. Heianza Y, Zhou T, Sun D, Hu FB, Qi L. Healthful plant-based dietary patterns, genetic risk of obesity, and cardiovascular risk in the UK biobank study. Clin Nutr. 2021 Jul;40(7):4694-4701. doi: 10.1016/j.clnu.2021.06.018. Epub 2021 Jun 24. PMID: 34237696; PMCID: PMC8338907.
5. Chiuve SE, Fung TT, Rimm EB, Hu FB, McCullough ML, Wang M, Stampfer MJ, Willett WC. Alternative dietary indices both strongly predict risk of chronic disease. J Nutr. 2012 Jun;142(6):1009-18. doi: 10.3945/jn.111.157222. Epub 2012 Apr 18. PMID: 22513989; PMCID: PMC3738221.
6. McCullough ML, Feskanich D, Stampfer MJ, Giovannucci EL, Rimm EB, Hu FB, Spiegelman D, Hunter DJ, Colditz GA, Willett WC. Diet quality and major chronic disease risk in men and women: moving toward improved dietary guidance. Am J Clin Nutr. 2002 Dec;76(6):1261-71. doi: 10.1093/ajcn/76.6.1261. PMID: 12450892.
7. Sacks FM, Svetkey LP, Vollmer WM, Appel LJ, Bray GA, Harsha D, Obarzanek E, Conlin PR, Miller ER 3rd, Simons-Morton DG, Karanja N, Lin PH; DASH-Sodium Collaborative Research Group. Effects on blood pressure of reduced dietary sodium and the Dietary Approaches to Stop Hypertension (DASH) diet. DASH-Sodium Collaborative Research Group. N Engl J Med. 2001 Jan 4;344(1):3-10. doi: 10.1056/NEJM200101043440101. PMID: 11136953.
8. Fung TT, Chiuve SE, McCullough ML, Rexrode KM, Logroscino G, Hu FB. Adherence to a DASH-style diet and risk of coronary heart disease and stroke in women. Arch Intern Med. 2008 Apr 14;168(7):713-20. doi: 10.1001/archinte.168.7.713. Erratum in: Arch Intern Med. 2008 Jun 23;168(12):1276. PMID: 18413553.
9. Sotos-Prieto M, Bhupathiraju SN, Mattei J, Fung TT, Li Y, Pan A, Willett WC, Rimm EB, Hu FB. Association of Changes in Diet Quality with Total and Cause-Specific

Mortality. N Engl J Med. 2017 Jul 13;377(2):143-153. doi: 10.1056/NEJMoa1613502. PMID: 28700845; PMCID: PMC5589446.
10. Trichopoulou A, Costacou T, Bamia C, Trichopoulos D. Adherence to a Mediterranean diet and survival in a Greek population. N Engl J Med. 2003 Jun 26;348(26):2599-608. doi: 10.1056/NEJMoa025039. PMID: 12826634.
11. Delgado-Lista J, Alcala-Diaz JF, Torres-Peña JD, Quintana-Navarro GM, Fuentes F, Garcia-Rios A, Ortiz-Morales AM, Gonzalez-Requero AI, Perez-Caballero AI, Yubero-Serrano EM, Rangel-Zuñiga OA, Camargo A, Rodriguez-Cantalejo F, Lopez-Segura F, Badimon L, Ordovas JM, Perez-Jimenez F, Perez-Martinez P, Lopez-Miranda J; CORDIOPREV Investigators. Long-term secondary prevention of cardiovascular disease with a Mediterranean diet and a low-fat diet (CORDIOPREV): a randomised controlled trial. Lancet. 2022 May 14;399(10338):1876-1885. doi: 10.1016/S0140-6736(22)00122-2. Epub 2022 May 4. PMID: 35525255.
12. Livingstone KM, Milte C, Bowe SJ, Duckham RL, Ward J, Keske MA, McEvoy M, Brayner B, Abbott G. Associations between three diet quality indices, genetic risk and body composition: A prospective cohort study. Clin Nutr. 2022 Sep;41(9):1942-1949. doi: 10.1016/j.clnu.2022.07.005. Epub 2022 Jul 10. PMID: 35947896.
13. Shivappa N, Steck SE, Hurley TG, Hussey JR, Hébert JR. Designing and developing a literature-derived, population-based dietary inflammatory index. Public Health Nutr. 2014 Aug;17(8):1689-96. doi: 10.1017/S1368980013002115. Epub 2013 Aug 14. PMID: 23941862; PMCID: PMC3925198.
14. Shi Y, Lin F, Li Y, Wang Y, Chen X, Meng F, Ye Q, Cai G. Association of pro-inflammatory diet with increased risk of all-cause dementia and Alzheimer's dementia: a prospective study of 166,377 UK Biobank participants. BMC Med. 2023 Jul 21;21(1):266. doi: 10.1186/s12916-023-02940-5. PMID: 37480061; PMCID: PMC10362711.
15. Satija A, Bhupathiraju SN, Rimm EB, Spiegelman D, Chiuve SE, Borgi L, Willett WC, Manson JE, Sun Q, Hu FB. Plant-Based Dietary Patterns and Incidence of Type 2 Diabetes in US Men and Women: Results from Three Prospective Cohort Studies. PLoS Med. 2016 Jun 14;13(6):e1002039. doi: 10.1371/journal.pmed.1002039. PMID: 27299701; PMCID: PMC4907448.
16. Satija A, Bhupathiraju SN, Spiegelman D, Chiuve SE, Manson JE, Willett W, Rexrode KM, Rimm EB, Hu FB. Healthful and Unhealthful Plant-Based Diets and the Risk of Coronary Heart Disease in U.S. Adults. J Am Coll Cardiol. 2017 Jul 25;70(4):411-422. doi: 10.1016/j.jacc.2017.05.047. PMID: 28728684; PMCID: PMC5555375.
17. Tresserra-Rimbau A, Thompson AS, Bondonno N, Jennings A, Kühn T, Cassidy A. Plant-Based Dietary Patterns and Parkinson's Disease: A Prospective Analysis of the UK Biobank. Mov Disord. 2023 Nov;38(11):1994-2004. doi: 10.1002/mds.29580. Epub 2023 Aug 21. PMID: 37602951.

Supplementary Table S1. Frailty phenotype and criteria for scoring

Individual components	Field IDs	Field description	Coding criteria
Weight Loss	2306	Compared with one year ago, has your weight changed	1="Yes - lost weight"<br>0="No - weigh about the same" or "Yes - gained weight"<br>NA="Do not know" or "Prefer not to answer"
Slow gait speed	924	How would you describe your usual walking pace	1="Slow pace"<br>0="Steady average pace" or "Brisk pace" or "None of the above"<br>NA="Do not know" or "Prefer not to answer"
Exhaustion	2080	Over the past two weeks, how often have you felt tired or had little energy	1="More than half the days" or "Nearly every day"<br>0="Not at all" or "Several days"<br>NA="Do not know" or "Prefer not to answer"
Low physical activity	6164	Types of physical activity in last 4 weeks	1=none or light activity with a frequency of once per week or less
	2624	Frequency of heavy DIY in last 4 weeks	0=medium or heavy activity, or light activity more than once per week
	1011	Frequency of light DIY in last 4 weeks	
	3637	Frequency of other exercises in last 4 weeks	
	943	Frequency of stair climbing in last 4 weeks	
	991	Frequency of strenuous sports in last 4 weeks	
	971	Frequency of walking for pleasure in last 4 weeks	
Low grip strength	31	sex	The higher of the two measured grip strength expressed in kg by sex- and BMI adjusted cut-off points.<br>Male<br>If BMI ≤24.0 kg/m2 & grip strength ≤29 kg<br>If BMI 24.1 to 26.0 kg/m2 & grip strength ≤30 kg<br>If BMI 26.1 to 28.0 kg/m2 & grip strength ≤30 kg<br>If BMI >28.0 kg/m2 & grip strength ≤32 kg<br>Female<br>If BMI ≤23.0 kg/m2 & grip strength ≤17 kg<br>If BMI 23.1 to 26.0 kg/m2 & grip strength ≤17.3 kg<br>If BMI 26.1 to 29.0 kg/m2 & grip strength ≤18 kg<br>If BMI >29.0 kg/m2 & grip strength ≤21 kg<br>If data on BMI or grip strength is not available = missing data.
	21001	Body mass index (BMI)	
	46	Hand grip strength (left)	
	47	Hand grip strength (right)	

Supplementary Table S2. Frailty index and criteria for scoring

Type of deficit	Item	Trait	Coding
Sensory	1	Glaucoma	yes=1, no=0, do not know/prefer not to answer=missing
	2	Cataracts	yes=1, no=0, do not know/prefer not to answer=missing
	3	Hearing difficulty	yes=1, completely deaf=1, no=0, do not know/prefer not to answer=missing
Cranial	4	Migraine	yes=1, no=0, do not know/prefer not to answer=missing
	5	Dental problems	yes=1, no=0, do not know/prefer not to answer=missing
Mental wellbeing	6	Self-rated health	excellent=0, good=0.25, fair=0.5, poor=1, do not know/prefer not to answer=missing
	7	Fatigue: frequency of tiredness / lethargy in last two weeks	not at all=0, several days=0.25, more than half the days=0.5, nearly every day=1, do not know/prefer not to answer=missing
	8	Sleep: experience of sleeplessness/insomnia	never/rarely=0, sometimes=0.5, usually=1, prefer not to answer=missing
	9	Depressed feelings: frequency in last two weeks	not at all=0, several days=0.5, more than half the days=0.75, nearly every day=1, do not know/prefer not to answer=missing
	10	Self-described nervous personality	yes=1, no=0, do not know/prefer not to answer=missing
	11	Severe anxiety/ panic attacks	yes=1, no=0, do not know/prefer not to answer=missing
	12	Common to feel loneliness	yes=1, no=0, do not know/prefer not to answer=missing
	13	Sense of misery (ever/never)	yes=1, no=0, do not know/prefer not to answer=missing
Infirmity	14	Infirmity: long-standing illness or disability	yes=1, no=0, do not know/prefer not to answer=missing
	15	Falls in last year	no falls=0, only one fall=0.5, more than one fall=1, prefer not to answer=missing
	16	Fractures/broken bones in last five years	yes=1, no=0, do not know/prefer not to answer=missing
Cardiometabolic	17	Diabetes	yes=1, no=0, do not know/prefer not to answer=missing
	18	Myocardial infarction	yes=1, no=0, do not know/prefer not to answer=missing
	19	Angina	yes=1, no=0, do not know/prefer not to answer=missing
	20	Stroke	yes=1, no=0, do not know/prefer not to answer=missing
	21	High blood pressure	yes=1, no=0, do not know/prefer not to answer=missing
	22	Hypothyroidism	yes=1, no=0, do not know/prefer not to answer=missing
	23	Deep-vein thrombosis	yes=1, no=0, do not know/prefer not to answer=missing
	24	High cholesterol	yes=1, no=0, do not know/prefer not to answer=missing
Respiratory	25	Breathing: wheeze in last year	yes=1, no=0, do not know/prefer not to answer=missing
	26	Pneumonia	yes=1, no=0, do not know/prefer not to answer=missing
	27	Chronic bronchitis/emphysema	yes=1, no=0, do not know/prefer not to answer=missing
	28	Asthma	yes=1, no=0, do not know/prefer not to answer=missing
Musculoskeletal	29	Rheumatoid arthritis	yes=1, no=0, do not know/prefer not to answer=missing
	30	Osteoarthritis	yes=1, no=0, do not know/prefer not to answer=missing
	31	Gout	yes=1, no=0, do not know/prefer not to answer=missing
	32	Osteoporosis	yes=1, no=0, do not know/prefer not to answer=missing
Immunological	33	Hayfever, allergic rhinitis or eczema	yes=1, no=0, do not know/prefer not to answer=missing
	34	Psoriasis	yes=1, no=0, do not know/prefer not to answer=missing
Cancer	35	Any cancer diagnosis	yes=1, no=0, do not know/prefer not to answer=missing
	36	Multiple cancers diagnosed (number reported)	no cancer=0, one cancer only=0, more than one cancer=1, prefer not to answer=missing
Pain	37	Chest pain	yes=1, no=0, do not know/prefer not to answer=missing
	38	Head and/or neck pain	yes=1, no=0, prefer not to answer=missing

	39	Back pain	yes=1, no=0, prefer not to answer=missing
	40	Stomach/abdominal pain	yes=1, no=0, prefer not to answer=missing
	41	Hip pain	yes=1, no=0, prefer not to answer=missing
	42	Knee pain	yes=1, no=0, prefer not to answer=missing
	43	Whole-body pain	yes=1, no=0, prefer not to answer=missing
	44	Facial pain	yes=1, no=0, prefer not to answer=missing
	45	Sciatica	yes=1, no=0, do not know/prefer not to answer=missing
Gastrointestinal	46	Gastric reflux	yes=1, no=0, do not know/prefer not to answer=missing
	47	Hiatus hernia	yes=1, no=0, do not know/prefer not to answer=missing
	48	Gall stones	yes=1, no=0, do not know/prefer not to answer=missing
	49	Diverticulitis	yes=1, no=0, do not know/prefer not to answer=missing

Supplementary Table S3. Alternate Healthy Eating Index (AHEI) components and criteria for scoring

	**Criteria for minimum score of 0**	**Criteria for maximum score of 10**	**Field ID**
Vegetables (excluding potatoes)	0	≥5 servings/d	104060, 104070, 104080, 104090, 104100, 104130, 104140, 104150, 104160, 104170, 104180, 104190, 104200, 104210, 104220, 104230, 104240, 104250, 104260, 104270, 104290, 104300, 104310, 104320, 104330, 104340, 104350, 104360, 104370, 104380
Fruits	0	≥4 servings/d	102490, 104410, 104420, 104430, 104440, 104450, 104460, 104470, 104480, 104490, 104500, 104510, 104520, 104530, 104540, 104550, 104560, 104570, 104580, 104590
Whole grain	0	Women: ≥5 servings/d; Men: ≥6 servings/d	100770, 100800, 100810, 100840, 100850, 100860, 100950, 101020, 101090, 101160, 101250, 101260, 101270, 102720, 102740, 102770, 102780, 20091, 20092, 20093, 20094
Nuts and legumes	0	≥1 serving/d	103260, 103270, 103280, 103290, 104000, 104010, 104110, 104120, 104280, 102410, 102420, 102430, 102440, 102450
Long-chain (n-3) fats	0	≥250 mg/d	26015
trans Fat	≥4% of energy	≤0.5% of energy	26155
Polyunsaturated fatty acids [a]	≤2% of energy	≥10% of energy	26016
Red and processed meats	≥1.5 servings/d	0	103010, 103020, 103030, 103040, 103070, 103080
Sodium	Highest decile	Lowest decile	26052
Alcohol	Women: ≥ 2.5 drinks/day<br>Men: ≥3.5 drinks/day	Women: 0.5-1.5 drinks/day<br>Men: 0.5-2.0 drinks/day	100022
Sugar-sweetened beverages and fruit juice	≥1 serving/d	0	100160, 100170, 100180, 100190, 100200, 100210, 100220

[a] Polyunsaturated fatty acids does not include long-chain n-3 fats.

Each AHEI component contributed from 0 to 10 points to the total AHEI score. For each component, a score of 10 indicates that the recommendations were fully met, whereas a score of 0 represents the least healthy dietary behavior. All component were summed to obtain the total AHEI score (range, 0-110).

Supplementary Table S4. Dietary Approaches to Stop Hypertension (DASH) components and criteria for scoring

	**Criteria for minimum score of 1**	**Criteria for maximum score of 5**	**Field ID**
Vegetables (excluding potatoes), servings/d	Lowest quintile	Highest quintile	104060, 104070, 104080, 104090, 104100, 104130, 104140, 104150, 104160, 104170, 104180, 104190, 104200, 104210, 104220, 104230, 104240, 104250, 104260, 104270, 104290, 104300, 104310, 104320, 104340, 104350, 104360, 104370, 104380, 104330
Fruits, servings/d	Lowest quintile	Highest quintile	104410, 104420, 104430, 104440, 104450, 104460, 104470, 104480, 104490, 104500, 104510, 104520, 104530, 104540, 104550, 104560, 104570, 104580, 104590, 102490
Nuts and legumes, servings/d	Lowest quintile	Highest quintile	104000, 104010, 104280, 102410, 102420, 102430, 102440, 102450, 103260, 103270, 103280, 103290, 104110, 104120
Low fat dairy, servings/d	Lowest quintile	Highest quintile	102810, 102850
Whole grain, servings/d	Lowest quintile	Highest quintile	100770, 100800, 100810, 100840, 100850, 100860, 100950, 101020, 101090, 101160, 101250, 101260, 101270, 102720, 102740, 102770, 102780， 20091,20092, 20093, 20094
Red and processed meats, servings/d	Highest quintile	Lowest quintile	103010, 103020, 103030, 103040, 103070, 103080
Sodium, mg/d	Highest quintile	Lowest quintile	26052
Sugar-sweetened beverages, servings/d	Highest quintile	Lowest quintile	100160, 100170, 100180

Each DASH component contributed from 0 to 5 points to the total DASH score. For each component, a score of 5 indicates that the recommendations were fully met, whereas a score of 1 represents the least healthy dietary behavior. All component were summed to obtain the total DASH score (range, 8-40).

Supplementary Table S5. Mediterranean Diet Score components and criteria for scoring

	1 score	Field ID
Vegetables (excluding potatoes), serving/d	≥ sex-specific median	104060, 104070, 104080, 104090, 104100, 104130, 104140, 104150, 104160, 104170, 104180, 104190, 104200, 104210, 104220, 104230, 104240, 104250, 104260, 104270, 104290, 104300, 104310, 104320, 104330, 104340, 104350, 104360, 104370, 104380
Fruits and nuts, serving/d	≥ sex-specific median	104410, 104420, 104430, 104440, 104450, 104460, 104470, 104480, 104490, 104500, 104510, 104520, 104530, 104540, 104550, 104560, 104570, 104580, 104590, 102490, 102410, 102420, 102430, 102440, 102450
Legumes, serving/d	≥ sex-specific median	103260, 103270, 103280, 103290, 104000, 104010, 104280, 104110, 104120
Whole grains, serving/d	≥ sex-specific median	100770, 100800, 100810, 100840, 100850, 100860, 100950, 101020, 101090, 101160, 101250, 101260, 101270, 102720, 102740, 102770, 102780， 20091,20092, 20093, 20094
Fish, serving/d	≥ sex-specific median	103150, 103160, 103170, 103180, 103190, 103230
MUFA:SFA ratio	≥ sex-specific median	26014, 26032
Red meats and processed meats, serving/d	≤ sex-specific median	103010, 103020, 103030, 103040, 103070, 103080
Alcohol, gram	5-15 g/d for women and 10-25 g/d for men	26038
Dairy products, serving/d	≤ sex-specific median	100230, 100520, 100530, 102810, 102820, 102830, 102840, 102850, 102860, 102870, 102880, 102890, 102900, 102910, 102090, 102120, 102140, 102150, 102220

Abbreviations: MUFA, monounsaturated fatty acid; SFA, saturated fatty acid.

The sex-specific median intakes of included participants were used to assign a score of 0 or 1 to each component. For each component, a score of 1 indicates that the recommendations were fully met, whereas a score of 0 represents the least healthy dietary behavior. All component were summed to obtain the total MED score (range, 0-9).

Supplementary Table S6. Dietary inflammatory index (DII) components and criteria for scoring

	**Overall inflammatory effect score**	**Global daily mean intake (units/day)**	**SD**	**Field ID**
Alcohol (g)	-0.278	13.98	3.72	26030
Vitamin B12 (μg)	0.106	5.15	2.7	26021
Vitamin B6 (mg)	−0.365	1.47	0.74	26020
β-Carotene (μg)	−0.584	3718	1720	26039
Carbohydrate (g)	0.097	272.2	40	26013
Cholesterol (mg)	0.11	279.4	51.2	26037
Energy (kcal)	0.180	2056	338	26002
Total fat (g)	0.298	71.4	19.4	26008
Fibre (g)	−0.663	18.8	4.9	26017
Folic acid (μg)	−0.190	273	70.7	26022
Fe (mg)	0.032	13.35	3.71	26019
Mg (mg)	−0.484	310.1	139.4	26025
MUFA (g)	−0.009	27	6.1	26032
Niacin (mg)	−0.246	25.9	11.77	26054
n-3 Fatty acids (g)	−0.436	1.06	1.06	26015
n-6 Fatty acids (g)	−0.159	10.8	7.5	26016
Protein (g)	0.021	79.4	13.9	26005
Riboflavin (mg)	−0.068	1.7	0.79	26035
Saturated fat (g)	0.373	28.6	8	26014
Se (μg)	−0.191	67	25.1	26058
Thiamin (mg)	−0.098	1.7	0.66	26034
Trans fat (g)	0.229	3.15	3.75	26155
Vitamin A (RE)	−0.401	983.9	518.6	26061
Vitamin C (mg)	−0.424	118.2	43.46	26023
Vitamin D (μg)	−0.446	6.26	2.21	26029
Vitamin E (mg)	−0.419	8.73	1.49	26028
Zn (mg)	−0.313	9.84	2.19	26033
PUFA (g)*	−0.337	13.88	3.76	26015,26016

*PUFA is calculated by the sum of n-3 Fatty acids (g) and n-6 Fatty acids (g).

The mean intake of every food variable was transformed with standardized values from a world database into a *z*-score, converted to a percentile, and centered by doubling and subtracting 1. Then, the centered percentile score for each food variable was multiplied by its associated literature-derived inflammatory effect score and these scores were summed across the dietary variables, thus providing an individual DII score. The higher the DII score, the more proinflammatory the diet; more negative values indicate more anti-inflammatory diets.

Supplementary Table S7. Plant-based diet index and its subtypes components and criteria for scoring

Food groups	Food items	Field ID	Scoring for PDI	Scoring for HPDI	Scoring for UPDI
**Plant-based food groups**					
**Healthy**					
Whole grains	Porridge, muesli, oat crunch, bran cereal, cereal bar, non-white bread (flour types, brown, whole meal, other type), seeded or other bread, crispbread, whole-wheat cereal, other cereal, whole meal pasta, brown rice, couscous, other cooked grains, oatcakes intake, other grain intake	100770, 100800, 100810, 100840, 100850, 100860, 100950, 101020, 101090, 101160, 101250, 101260, 101270, 102720, 102740, 102770, 102780, 20091, 20092, 20093, 20094	Positive	Positive	Reverse
Fruits	Stewed fruit, prune, dried fruit, mixed fruit, apple, banana, berries, cherries, grapefruit, grapes, mango, melon, orange, orange-like small fruits, peach/nectarine, pear, pineapple, plum, other fruits, olives	104410, 104420, 104430, 104440, 104450, 104460, 104470, 104480, 104490, 104500, 104510, 104520, 104530, 104540, 104550, 104560, 104570, 104580, 104590, 102490	Positive	Positive	Reverse
Vegetables	Mixed vegetables, vegetable pieces, coleslaw, side salad, avocado, beetroot, broccoli, butternut squash, cabbage/kale, carrots, cauliflower, celery, courgette, cucumber, garlic, leeks, lettuce, mushrooms, onion, parsnip, sweet peppers, spinach, sprouts, sweetcorn, sweet potato, fresh tomatoes, cooked or tinned tomatoes, turnip/swede, watercress, other vegetable intake	104060, 104070, 104080, 104090, 104100, 104130, 104140, 104150, 104160, 104170, 104180, 104190, 104200, 104210, 104220, 104230, 104240, 104250, 104260, 104270, 104290, 104300, 104310, 104320, 104330, 104340, 104350, 104360, 104370, 104380	Positive	Positive	Reverse
Nuts	Salted peanuts, unsalted peanuts, salted nuts, unsalted nuts, seeds	102410, 102420, 102430, 102440, 102450	Positive	Positive	Reverse
Legumes	Vegetarian sausages/burgers, tofu, quorn, other vegetarian alternative, baked beans, pulse, broad beans, green beans, peas	103260, 103270, 103280, 103290, 104000, 104010, 104110, 104120, 104280	Positive	Positive	Reverse
Tea and coffee	Instant coffee, filtered coffee, cappuccino, latte, espresso, other coffee drinks, standard tea, rooibos tea, green tea, herbal tea, other tea	100250, 100270, 100290, 100300, 100310, 100330, 100400, 100410, 100420, 100430, 100440	Positive	Positive	Reverse
**Less healthy**					
Refined grains	Sweetened cereal, plain cereal, white bread, naan bread, garlic bread, white pasta, white rice, pancake, snackpot, scotch pancake, croissant, scone, savoury or cheesy biscuits, other savoury snack	100820, 100830, 100950, 101020, 101090, 101160, 101230, 101240, 102710, 102730, 102760, 102010, 102020, 102050, 102070, 102470, 102480, 102500, 20091,20092, 20093, 20094	Positive	Reverse	Positive
Potatoes	Fried potatoes, boiled/baked potatoes, mashed potatoes, crisps (e.g., potato chips)	102460, 104020, 104030, 104050	Positive	Reverse	Positive
Sugary drinks	Low calorie or diet drinks (e.g. fizzy, squash), carbonated (fizzy) drinks, squash or cordial	100160, 100170, 100180	Positive	Reverse	Positive
Fruit juices	Orange juice, grapefruit juice, other fruit/vegetable juice, fruit smoothie	100190, 100200, 100210, 100220	Positive	Reverse	Positive
Sweets and desserts	Double crust pie, single crust pie/flan, crumble topping, Yorkshire pudding, Danish pastry, fruitcake, cake, doughnuts, sponge pudding, other dessert, chocolate bar, white chocolate, milk chocolate, dark chocolate, chocolate-covered raisin, chocolate sweet, diet sweets, chocolate-covered biscuits, chocolate biscuits, sweet biscuits, other sweets	101970, 101980, 101990, 102030, 102060, 102170, 102180, 102190, 102200, 102210, 102230, 102260, 102270, 102280, 102290, 102300, 102310, 102320, 102330, 102340, 102350, 102360, 102380	Positive	Reverse	Positive
**Animal-based food groups**					

Animal fat	Butter on bread/crackers (spreadable, low fat, normal fat, or unknown type), dairy spread on bread/crackers (very low fat, low fat, normal fat, unknown type)	101310, 101350, 101390, 101430, 101470, 101510, 101550	Reverse	Reverse	Reverse
Dairy	Milk, dairy smoothie, flavored milk, yogurt, ice-cream, cheesecake, milk-based pudding, other milk-based pudding, low fat hard cheese, hard cheese, soft cheese, blue cheese, low fat cheese spread, cheese spread, cottage cheese, feta cheese, mozzarella cheese, goat's cheese, other cheese	100230, 100520, 100530, 102090, 102120, 102140, 102150, 102220, 102810, 102820, 102830, 102840, 102850, 102860, 102870, 102880, 102890, 102900, 102910	Reverse	Reverse	Reverse
Eggs	Whole eggs, omelettes or scrambled egg, eggs in sandwiches, scotch egg, other egg dishes	102940, 102950, 102960, 102970, 102980	Reverse	Reverse	Reverse
Fish or seafood	Tinned tuna, oily fish, breaded fish, battered fish, white fish, prawns, lobster/crab, shellfish, other fish intake	103150, 103160, 103170, 103180, 103190, 103200, 103210, 103220, 103230	Reverse	Reverse	Reverse
Meat	Sausage, beef, pork, lamb, crumbed or deep-fried poultry, poultry, bacon, ham, liver, other meat intake	103010, 103020, 103030, 103040, 103050, 103060, 103070, 103080, 103090, 103100	Reverse	Reverse	Reverse
Miscellaneous animal-based foods	Pizza, Indian snacks	102000, 102040	Reverse	Reverse	Reverse

Abbreviations: PDI, plant-based diet index; HPDI, healthful plant-based diet index; UPDI, unhealthful plant-based diet index. The intakes of every food group were ranked into quintiles and given positive (Q1 to Q5 received 1 to 5 point) or reverse (Q1 to Q5 received 5 to 1 point) scores. To generate the overall PDI, healthful and unhealthful plant-based food groups were given positive scores, and animal food groups received reverse scores. For HPDI, positive scores were given to healthy plant-based food groups, and reverse scores were given to less healthy plant-based food groups and animal food groups. For creating UPDI, positive scores were given to less healthy plant-based food groups, and reverse scores were given to healthy plant-based food groups and animal food groups.

Supplementary Table S8. Baseline characteristics of the participants by AHEI

	**Unhealthy tertile**	**Medium Tertile**	**Healthy Tertile**	**Overall**
	**(N=50543)**	**(N=50542)**	**(N=50543)**	**(N=151628)**
Age at recruitment, years, mean (SD)	57.1 (6.8)	57.9 (6.7)	58.3 (6.5)	57.8 (6.7)
Sex, n (%)				
female	20827 (41.2%)	27440 (54.3%)	33619 (66.5%)	81886 (54.0%)
male	29716 (58.8%)	23102 (45.7%)	16924 (33.5%)	69742 (46.0%)
Body mass index (BMI), kg/m^2, n (%)				
<25.0	15304 (30.3%)	18694 (37.0%)	22273 (44.1%)	56271 (37.1%)
25.0-29.9	22709 (44.9%)	21679 (42.9%)	19766 (39.1%)	64154 (42.3%)
>=30.0	12530 (24.8%)	10169 (20.1%)	8504 (16.8%)	31203 (20.6%)
Ethnicity, n (%)				
white	49386 (97.7%)	49091 (97.1%)	48441 (95.8%)	146918 (96.9%)
other	1157 (2.3%)	1451 (2.9%)	2102 (4.2%)	4710 (3.1%)
Education, n (%)				
college or University degree	20732 (41.0%)	21762 (43.1%)	22614 (44.7%)	65108 (42.9%)
secondary school	19726 (39.0%)	18674 (36.9%)	18196 (36.0%)	56596 (37.3%)
primary school	4508 (8.9%)	4533 (9.0%)	4286 (8.5%)	13327 (8.8%)
professional qualification	5577 (11.0%)	5573 (11.0%)	5447 (10.8%)	16597 (10.9%)
Employment, n (%)				
employed	30036 (59.4%)	27989 (55.4%)	27225 (53.9%)	85250 (56.2%)
other	20507 (40.6%)	22553 (44.6%)	23318 (46.1%)	66378 (43.8%)
Household income, £/year, n (%)				
less than 18000	7703 (15.2%)	8624 (17.1%)	9563 (18.9%)	25890 (17.1%)
18000 to 30999	11839 (23.4%)	12768 (25.3%)	13242 (26.2%)	37849 (25.0%)
31000 to 51999	14026 (27.8%)	13811 (27.3%)	13585 (26.9%)	41422 (27.3%)
52000 to 100000	12576 (24.9%)	11608 (23.0%)	10708 (21.2%)	34892 (23.0%)
greater than 100000	4399 (8.7%)	3731 (7.4%)	3445 (6.8%)	11575 (7.6%)
Townsend deprivation index, n (%)				
first quartile	12588 (24.9%)	12951 (25.6%)	12395 (24.5%)	37934 (25.0%)
second quartile	12299 (24.3%)	12882 (25.5%)	12701 (25.1%)	37882 (25.0%)
third quartile	12583 (24.9%)	12672 (25.1%)	12651 (25.0%)	37906 (25.0%)
fourth quartile	13073 (25.9%)	12037 (23.8%)	12796 (25.3%)	37906 (25.0%)
Smoking status, n (%)				
never	25862 (51.2%)	28985 (57.3%)	30244 (59.8%)	85091 (56.1%)
previous	19676 (38.9%)	18288 (36.2%)	17831 (35.3%)	55795 (36.8%)
current	5005 (9.9%)	3269 (6.5%)	2468 (4.9%)	10742 (7.1%)
Alcohol drinking frequency, n (%)				
>= 3 times/week	30456 (60.3%)	24790 (49.0%)	21073 (41.7%)	76319 (50.3%)
< 3 times/week	17947 (35.5%)	22872 (45.3%)	25570 (50.6%)	66389 (43.8%)
never	2140 (4.2%)	2880 (5.7%)	3900 (7.7%)	8920 (5.9%)
Sleep duration, n (%)				
7-8 h/day	35617 (70.5%)	36168 (71.6%)	36136 (71.5%)	107921 (71.2%)
< 7h/day	11413 (22.6%)	11046 (21.9%)	11190 (22.1%)	33649 (22.2%)
> 8h/day	3513 (7.0%)	3328 (6.6%)	3217 (6.4%)	10058 (6.6%)
Physical activity, n (%)				
high	18332 (36.3%)	19775 (39.1%)	21622 (42.8%)	59729 (39.4%)
moderate	20215 (40.0%)	20249 (40.1%)	19794 (39.2%)	60258 (39.7%)

low	11996 (23.7%)	10518 (20.8%)	9127 (18.1%)	31641 (20.9%)
Frailty index, n (%)				
robust	30429 (60.2%)	31287 (61.9%)	31698 (62.7%)	93414 (61.6%)
pre-frailty	17609 (34.8%)	17024 (33.7%)	16760 (33.2%)	51393 (33.9%)
frailty	2505 (5.0%)	2231 (4.4%)	2085 (4.1%)	6821 (4.5%)
Frailty phenotype, n (%)				
robust	31712 (62.7%)	32293 (63.9%)	32692 (64.7%)	96697 (63.8%)
pre-frailty	17646 (34.9%)	17259 (34.1%)	16944 (33.5%)	51849 (34.2%)
frailty	1185 (2.3%)	990 (2.0%)	907 (1.8%)	3082 (2.0%)
Energy, kcal, mean (SD)	2170 (537)	2000 (508)	1930 (497)	2030 (524)
DASH, n (%)				
unhealthy tertile	41821 (82.7%)	12324 (24.4%)	132 (0.3%)	54277 (35.8%)
medium tertile	8251 (16.3%)	30865 (61.1%)	10233 (20.2%)	49349 (32.5%)
healthy tertile	471 (0.9%)	7353 (14.5%)	40178 (79.5%)	48002 (31.7%)
MED, n (%)				
unhealthy tertile	30656 (60.7%)	16333 (32.3%)	6310 (12.5%)	53299 (35.2%)
medium tertile	17197 (34.0%)	24608 (48.7%)	22265 (44.1%)	64070 (42.3%)
healthy tertile	2690 (5.3%)	9601 (19.0%)	21968 (43.5%)	34259 (22.6%)
DII, n (%)				
healthy tertile	12425 (24.6%)	15660 (31.0%)	22458 (44.4%)	50543 (33.3%)
medium tertile	16895 (33.4%)	17073 (33.8%)	16574 (32.8%)	50542 (33.3%)
unhealthy tertile	21223 (42.0%)	17809 (35.2%)	11511 (22.8%)	50543 (33.3%)
PDI, n (%)				
unhealthy tertile	28558 (56.5%)	19246 (38.1%)	11733 (23.2%)	59537 (39.3%)
medium tertile	14127 (28.0%)	16015 (31.7%)	15068 (29.8%)	45210 (29.8%)
healthy tertile	7858 (15.5%)	15281 (30.2%)	23742 (47.0%)	46881 (30.9%)
HPDI, n (%)				
unhealthy tertile	35132 (69.5%)	17368 (34.4%)	3251 (6.4%)	55751 (36.8%)
medium tertile	12103 (23.9%)	20970 (41.5%)	13179 (26.1%)	46252 (30.5%)
healthy tertile	3308 (6.5%)	12204 (24.1%)	34113 (67.5%)	49625 (32.7%)
UPDI, n (%)				
healthy tertile	8169 (16.2%)	15793 (31.2%)	29629 (58.6%)	53591 (35.3%)
medium tertile	15411 (30.5%)	18015 (35.6%)	14811 (29.3%)	48237 (31.8%)
unhealthy tertile	26963 (53.3%)	16734 (33.1%)	6103 (12.1%)	49800 (32.8%)
Family history of CVD, n (%)				
no	12905 (25.5%)	11936 (23.6%)	11498 (22.7%)	36339 (24.0%)
yes	37638 (74.5%)	38606 (76.4%)	39045 (77.3%)	115289 (76.0%)
Family history of cancer, n (%)				
no	31631 (62.6%)	31727 (62.8%)	31847 (63.0%)	95205 (62.8%)
yes	18912 (37.4%)	18815 (37.2%)	18696 (37.0%)	56423 (37.2%)
Family history of diabetes, n (%)				
no	40087 (79.3%)	39840 (78.8%)	39838 (78.8%)	119765 (79.0%)
yes	10456 (20.7%)	10702 (21.2%)	10705 (21.2%)	31863 (21.0%)

AHEI, Alternative Healthy Eating Index; DASH, Dietary Approaches to Stop Hypertension; MED, Mediterranean Diet; DII, Dietary Inflammatory Index; PDI, Plant-Based Diet Index; HPDI, Healthy Plant-Based Diet Index; UPDI, Unhealthy Plant-Based Diet Index; CVD, cardiovascular disease; SD: standard deviation. Employment status is categorized as employed (includes paid employment or self- employed,

paid or voluntary work or student), and other (includes retired, looking after home and/or family, unable to work and unemployed). Education is categorized as college or University degree, secondary school (includes A levels/AS levels or equivalent, O levels/GCSEs or equivalent, CSEs or equivalent), primary school, and professional qualification (NVQ or HND or HNC or equivalent, other professional qualifications).

Supplementary Table S9. Baseline characteristics of the participants by DASH

	Unhealthy tertile (N=54277)	Medium Tertile (N=49349)	Healthy Tertile (N=48002)	Overall (N=151628)
Age at recruitment, years, mean (SD)	57.0 (6.8)	58.0 (6.7)	58.4 (6.5)	57.8 (6.7)
Sex, n (%)				
female	22732 (41.9%)	27003 (54.7%)	32151 (67.0%)	81886 (54.0%)
male	31545 (58.1%)	22346 (45.3%)	15851 (33.0%)	69742 (46.0%)
Body mass index (BMI), kg/m^2, n (%)				
<25.0	16345 (30.1%)	18580 (37.7%)	21346 (44.5%)	56271 (37.1%)
25.0-29.9	24197 (44.6%)	21026 (42.6%)	18931 (39.4%)	64154 (42.3%)
>=30.0	13735 (25.3%)	9743 (19.7%)	7725 (16.1%)	31203 (20.6%)
Ethnicity, n (%)				
white	52802 (97.3%)	47837 (96.9%)	46279 (96.4%)	146918 (96.9%)
other	1475 (2.7%)	1512 (3.1%)	1723 (3.6%)	4710 (3.1%)
Education, n (%)				
college or University degree	21476 (39.6%)	21456 (43.5%)	22176 (46.2%)	65108 (42.9%)
secondary school	21464 (39.5%)	18163 (36.8%)	16969 (35.4%)	56596 (37.3%)
primary school	5177 (9.5%)	4378 (8.9%)	3772 (7.9%)	13327 (8.8%)
professional qualification	6160 (11.3%)	5352 (10.8%)	5085 (10.6%)	16597 (10.9%)
Employment, n (%)				
employed	32572 (60.0%)	27183 (55.1%)	25495 (53.1%)	85250 (56.2%)
other	21705 (40.0%)	22166 (44.9%)	22507 (46.9%)	66378 (43.8%)
Household income, £/year, n (%)				
less than 18000	8977 (16.5%)	8367 (17.0%)	8546 (17.8%)	25890 (17.1%)
18000 to 30999	12894 (23.8%)	12496 (25.3%)	12459 (26.0%)	37849 (25.0%)
31000 to 51999	15070 (27.8%)	13308 (27.0%)	13044 (27.2%)	41422 (27.3%)
52000 to 100000	13087 (24.1%)	11340 (23.0%)	10465 (21.8%)	34892 (23.0%)
greater than 100000	4249 (7.8%)	3838 (7.8%)	3488 (7.3%)	11575 (7.6%)
Townsend deprivation index, n (%)				
first quartile	13462 (24.8%)	12485 (25.3%)	11987 (25.0%)	37934 (25.0%)
second quartile	13373 (24.6%)	12428 (25.2%)	12081 (25.2%)	37882 (25.0%)
third quartile	13444 (24.8%)	12445 (25.2%)	12017 (25.0%)	37906 (25.0%)
fourth quartile	13998 (25.8%)	11991 (24.3%)	11917 (24.8%)	37906 (25.0%)
Smoking status, n (%)				
never	28939 (53.3%)	28054 (56.8%)	28098 (58.5%)	85091 (56.1%)
previous	20067 (37.0%)	18077 (36.6%)	17651 (36.8%)	55795 (36.8%)
current	5271 (9.7%)	3218 (6.5%)	2253 (4.7%)	10742 (7.1%)
Alcohol drinking frequency, n (%)				
>= 3 times/week	28746 (53.0%)	25226 (51.1%)	22347 (46.6%)	76319 (50.3%)
< 3 times/week	22668 (41.8%)	21327 (43.2%)	22394 (46.7%)	66389 (43.8%)
never	2863 (5.3%)	2796 (5.7%)	3261 (6.8%)	8920 (5.9%)
Sleep duration, n (%)				
7-8 h/day	38007 (70.0%)	35394 (71.7%)	34520 (71.9%)	107921 (71.2%)
< 7h/day	12511 (23.1%)	10626 (21.5%)	10512 (21.9%)	33649 (22.2%)
> 8h/day	3759 (6.9%)	3329 (6.7%)	2970 (6.2%)	10058 (6.6%)
Physical activity, n (%)				
high	19569 (36.1%)	19297 (39.1%)	20863 (43.5%)	59729 (39.4%)
moderate	21474 (39.6%)	19922 (40.4%)	18862 (39.3%)	60258 (39.7%)

low	13234 (24.4%)	10130 (20.5%)	8277 (17.2%)	31641 (20.9%)
Frailty index, n (%)				
robust	32168 (59.3%)	30744 (62.3%)	30502 (63.5%)	93414 (61.6%)
pre-frailty	19237 (35.4%)	16447 (33.3%)	15709 (32.7%)	51393 (33.9%)
frailty	2872 (5.3%)	2158 (4.4%)	1791 (3.7%)	6821 (4.5%)
Frailty phenotype, n (%)				
robust	33482 (61.7%)	31797 (64.4%)	31418 (65.5%)	96697 (63.8%)
pre-frailty	19395 (35.7%)	16614 (33.7%)	15840 (33.0%)	51849 (34.2%)
frailty	1400 (2.6%)	938 (1.9%)	744 (1.6%)	3082 (2.0%)
Energy, kcal, mean (SD)	2120 (545)	2010 (516)	1950 (490)	2030 (524)
AHEI, n (%)				
unhealthy tertile	41821 (77.1%)	8251 (16.7%)	471 (1.0%)	50543 (33.3%)
medium tertile	12324 (22.7%)	30865 (62.5%)	7353 (15.3%)	50542 (33.3%)
healthy tertile	132 (0.2%)	10233 (20.7%)	40178 (83.7%)	50543 (33.3%)
MED, n (%)				
unhealthy tertile	33165 (61.1%)	14941 (30.3%)	5193 (10.8%)	53299 (35.2%)
medium tertile	18340 (33.8%)	24741 (50.1%)	20989 (43.7%)	64070 (42.3%)
healthy tertile	2772 (5.1%)	9667 (19.6%)	21820 (45.5%)	34259 (22.6%)
DII, n (%)				
healthy tertile	13293 (24.5%)	15966 (32.4%)	21284 (44.3%)	50543 (33.3%)
medium tertile	17871 (32.9%)	16636 (33.7%)	16035 (33.4%)	50542 (33.3%)
unhealthy tertile	23113 (42.6%)	16747 (33.9%)	10683 (22.3%)	50543 (33.3%)
PDI, n (%)				
unhealthy tertile	31418 (57.9%)	18411 (37.3%)	9708 (20.2%)	59537 (39.3%)
medium tertile	14831 (27.3%)	15900 (32.2%)	14479 (30.2%)	45210 (29.8%)
healthy tertile	8028 (14.8%)	15038 (30.5%)	23815 (49.6%)	46881 (30.9%)
HPDI, n (%)				
unhealthy tertile	39416 (72.6%)	14192 (28.8%)	2143 (4.5%)	55751 (36.8%)
medium tertile	12484 (23.0%)	22277 (45.1%)	11491 (23.9%)	46252 (30.5%)
healthy tertile	2377 (4.4%)	12880 (26.1%)	34368 (71.6%)	49625 (32.7%)
UPDI, n (%)				
healthy tertile	7616 (14.0%)	16060 (32.5%)	29915 (62.3%)	53591 (35.3%)
medium tertile	16142 (29.7%)	18410 (37.3%)	13685 (28.5%)	48237 (31.8%)
unhealthy tertile	30519 (56.2%)	14879 (30.2%)	4402 (9.2%)	49800 (32.8%)
Family history of CVD, n (%)				
no	13724 (25.3%)	11790 (23.9%)	10825 (22.6%)	36339 (24.0%)
yes	40553 (74.7%)	37559 (76.1%)	37177 (77.4%)	115289 (76.0%)
Family history of cancer, n (%)				
no	34063 (62.8%)	31046 (62.9%)	30096 (62.7%)	95205 (62.8%)
yes	20214 (37.2%)	18303 (37.1%)	17906 (37.3%)	56423 (37.2%)
Family history of diabetes, n (%)				
no	42615 (78.5%)	39127 (79.3%)	38023 (79.2%)	119765 (79.0%)
yes	11662 (21.5%)	10222 (20.7%)	9979 (20.8%)	31863 (21.0%)

AHEI, Alternative Healthy Eating Index; DASH, Dietary Approaches to Stop Hypertension; MED, Mediterranean Diet; DII, Dietary Inflammatory Index; PDI, Plant-Based Diet Index; HPDI, Healthy Plant-Based Diet Index; UPDI, Unhealthy Plant-Based Diet Index; CVD, cardiovascular disease; SD: standard deviation. Employment status is categorized as employed (includes paid employment or self- employed,

paid or voluntary work or student), and other (includes retired, looking after home and/or family, unable to work and unemployed). Education is categorized as college or University degree, secondary school (includes A levels/AS levels or equivalent, O levels/GCSEs or equivalent, CSEs or equivalent), primary school, and professional qualification (NVQ or HND or HNC or equivalent, other professional qualifications).

Supplementary Table S10. Baseline characteristics of the participants by MED

	**Unhealthy tertile**	**Medium Tertile**	**Healthy Tertile**	**Overall**
	**(N=53299)**	**(N=64070)**	**(N=34259)**	**(N=151628)**
Age at recruitment, years, mean (SD)	57.2 (6.7)	57.9 (6.7)	58.4 (6.5)	57.8 (6.7)
Sex, n (%)				
female	30394 (57.0%)	33707 (52.6%)	17785 (51.9%)	81886 (54.0%)
male	22905 (43.0%)	30363 (47.4%)	16474 (48.1%)	69742 (46.0%)
Body mass index (BMI), kg/m^2, n (%)				
<25.0	17764 (33.3%)	23667 (36.9%)	14840 (43.3%)	56271 (37.1%)
25.0-29.9	22741 (42.7%)	27392 (42.8%)	14021 (40.9%)	64154 (42.3%)
>=30.0	12794 (24.0%)	13011 (20.3%)	5398 (15.8%)	31203 (20.6%)
Ethnicity, n (%)				
white	51732 (97.1%)	62057 (96.9%)	33129 (96.7%)	146918 (96.9%)
other	1567 (2.9%)	2013 (3.1%)	1130 (3.3%)	4710 (3.1%)
Education, n (%)				
college or University degree	20111 (37.7%)	27952 (43.6%)	17045 (49.8%)	65108 (42.9%)
secondary school	21535 (40.4%)	23718 (37.0%)	11343 (33.1%)	56596 (37.3%)
primary school	5695 (10.7%)	5424 (8.5%)	2208 (6.4%)	13327 (8.8%)
professional qualification	5958 (11.2%)	6976 (10.9%)	3663 (10.7%)	16597 (10.9%)
Employment, n (%)				
employed	31084 (58.3%)	35776 (55.8%)	18390 (53.7%)	85250 (56.2%)
other	22215 (41.7%)	28294 (44.2%)	15869 (46.3%)	66378 (43.8%)
Household income, £/year, n (%)				
less than 18000	10011 (18.8%)	10655 (16.6%)	5224 (15.2%)	25890 (17.1%)
18000 to 30999	13210 (24.8%)	16109 (25.1%)	8530 (24.9%)	37849 (25.0%)
31000 to 51999	14491 (27.2%)	17518 (27.3%)	9413 (27.5%)	41422 (27.3%)
52000 to 100000	11815 (22.2%)	14860 (23.2%)	8217 (24.0%)	34892 (23.0%)
greater than 100000	3772 (7.1%)	4928 (7.7%)	2875 (8.4%)	11575 (7.6%)
Townsend deprivation index, n (%)				
first quartile	12918 (24.2%)	16224 (25.3%)	8792 (25.7%)	37934 (25.0%)
second quartile	12990 (24.4%)	16198 (25.3%)	8694 (25.4%)	37882 (25.0%)
third quartile	13473 (25.3%)	16066 (25.1%)	8367 (24.4%)	37906 (25.0%)
fourth quartile	13918 (26.1%)	15582 (24.3%)	8406 (24.5%)	37906 (25.0%)
Smoking status, n (%)				
never	29364 (55.1%)	35971 (56.1%)	19756 (57.7%)	85091 (56.1%)
previous	18986 (35.6%)	23872 (37.3%)	12937 (37.8%)	55795 (36.8%)
current	4949 (9.3%)	4227 (6.6%)	1566 (4.6%)	10742 (7.1%)
Alcohol drinking frequency, n (%)				
>= 3 times/week	25514 (47.9%)	32604 (50.9%)	18201 (53.1%)	76319 (50.3%)
< 3 times/week	24262 (45.5%)	27760 (43.3%)	14367 (41.9%)	66389 (43.8%)
never	3523 (6.6%)	3706 (5.8%)	1691 (4.9%)	8920 (5.9%)
Sleep duration, n (%)				
7-8 h/day	36991 (69.4%)	45848 (71.6%)	25082 (73.2%)	107921 (71.2%)
< 7h/day	12452 (23.4%)	14072 (22.0%)	7125 (20.8%)	33649 (22.2%)
> 8h/day	3856 (7.2%)	4150 (6.5%)	2052 (6.0%)	10058 (6.6%)
Physical activity, n (%)				
high	19501 (36.6%)	25383 (39.6%)	14845 (43.3%)	59729 (39.4%)
moderate	21029 (39.5%)	25569 (39.9%)	13660 (39.9%)	60258 (39.7%)

low	12769 (24.0%)	13118 (20.5%)	5754 (16.8%)	31641 (20.9%)
Frailty index, n (%)				
robust	31433 (59.0%)	39768 (62.1%)	22213 (64.8%)	93414 (61.6%)
pre-frailty	18951 (35.6%)	21509 (33.6%)	10933 (31.9%)	51393 (33.9%)
frailty	2915 (5.5%)	2793 (4.4%)	1113 (3.2%)	6821 (4.5%)
Frailty phenotype, n (%)				
robust	32250 (60.5%)	41306 (64.5%)	23141 (67.5%)	96697 (63.8%)
pre-frailty	19551 (36.7%)	21614 (33.7%)	10684 (31.2%)	51849 (34.2%)
frailty	1498 (2.8%)	1150 (1.8%)	434 (1.3%)	3082 (2.0%)
Energy, kcal, mean (SD)	2010 (547)	2040 (521)	2150 (490)	2030 (524)
AHEI, n (%)				
unhealthy tertile	30656 (57.5%)	17197 (26.8%)	2690 (7.9%)	50543 (33.3%)
medium tertile	16333 (30.6%)	24608 (38.4%)	9601 (28.0%)	50542 (33.3%)
healthy tertile	6310 (11.8%)	22265 (34.8%)	21968 (64.1%)	50543 (33.3%)
DASH, n (%)				
unhealthy tertile	33165 (62.2%)	18340 (28.6%)	2772 (8.1%)	54277 (35.8%)
medium tertile	14941 (28.0%)	24741 (38.6%)	9667 (28.2%)	49349 (32.5%)
healthy tertile	5193 (9.7%)	20989 (32.8%)	21820 (63.7%)	48002 (31.7%)
DII, n (%)				
healthy tertile	8813 (16.5%)	22339 (34.9%)	19391 (56.6%)	50543 (33.3%)
medium tertile	16289 (30.6%)	23404 (36.5%)	10849 (31.7%)	50542 (33.3%)
unhealthy tertile	28197 (52.9%)	18327 (28.6%)	4019 (11.7%)	50543 (33.3%)
PDI, n (%)				
unhealthy tertile	31011 (58.2%)	22472 (35.1%)	6054 (17.7%)	59537 (39.3%)
medium tertile	14596 (27.4%)	20423 (31.9%)	10191 (29.7%)	45210 (29.8%)
healthy tertile	7692 (14.4%)	21175 (33.0%)	18014 (52.6%)	46881 (30.9%)
HPDI, n (%)				
unhealthy tertile	29729 (55.8%)	21073 (32.9%)	4949 (14.4%)	55751 (36.8%)
medium tertile	15393 (28.9%)	21286 (33.2%)	9573 (27.9%)	46252 (30.5%)
healthy tertile	8177 (15.3%)	21711 (33.9%)	19737 (57.6%)	49625 (32.7%)
UPDI, n (%)				
healthy tertile	10371 (19.5%)	23876 (37.3%)	19344 (56.5%)	53591 (35.3%)
medium tertile	16752 (31.4%)	21491 (33.5%)	9994 (29.2%)	48237 (31.8%)
unhealthy tertile	26176 (49.1%)	18703 (29.2%)	4921 (14.4%)	49800 (32.8%)
Family history of CVD, n (%)				
no	13179 (24.7%)	15400 (24.0%)	7760 (22.7%)	36339 (24.0%)
yes	40120 (75.3%)	48670 (76.0%)	26499 (77.3%)	115289 (76.0%)
Family history of cancer, n (%)				
no	33402 (62.7%)	40162 (62.7%)	21641 (63.2%)	95205 (62.8%)
yes	19897 (37.3%)	23908 (37.3%)	12618 (36.8%)	56423 (37.2%)
Family history of diabetes, n (%)				
no	42018 (78.8%)	50488 (78.8%)	27259 (79.6%)	119765 (79.0%)
yes	11281 (21.2%)	13582 (21.2%)	7000 (20.4%)	31863 (21.0%)

AHEI, Alternative Healthy Eating Index; DASH, Dietary Approaches to Stop Hypertension; MED, Mediterranean Diet; DII, Dietary Inflammatory Index; PDI, Plant-Based Diet Index; HPDI, Healthy Plant-Based Diet Index; UPDI, Unhealthy Plant-Based Diet Index; CVD, cardiovascular disease; SD: standard deviation. Employment status is categorized as employed (includes paid employment or self- employed,

paid or voluntary work or student), and other (includes retired, looking after home and/or family, unable to work and unemployed). Education is categorized as college or University degree, secondary school (includes A levels/AS levels or equivalent, O levels/GCSEs or equivalent, CSEs or equivalent), primary school, and professional qualification (NVQ or HND or HNC or equivalent, other professional qualifications).

Supplementary Table S11. Baseline characteristics of the participants by DII

	Healthy Tertile	Medium Tertile	Unhealthy Tertile	Overall
	(N=50543)	(N=50542)	(N=50543)	(N=151628)
Age at recruitment, years, mean (SD)	58.2 (6.6)	57.8 (6.6)	57.2 (6.7)	57.8 (6.7)
Sex, n (%)				
female	23491 (46.5%)	28059 (55.5%)	30336 (60.0%)	81886 (54.0%)
male	27052 (53.5%)	22483 (44.5%)	20207 (40.0%)	69742 (46.0%)
Body mass index (BMI), kg/m^2, n (%)				
<25.0	19251 (38.1%)	18949 (37.5%)	18071 (35.8%)	56271 (37.1%)
25.0-29.9	21365 (42.3%)	21592 (42.7%)	21197 (41.9%)	64154 (42.3%)
>=30.0	9927 (19.6%)	10001 (19.8%)	11275 (22.3%)	31203 (20.6%)
Ethnicity, n (%)				
white	49245 (97.4%)	49213 (97.4%)	48460 (95.9%)	146918 (96.9%)
other	1298 (2.6%)	1329 (2.6%)	2083 (4.1%)	4710 (3.1%)
Education, n (%)				
college or University degree	23134 (45.8%)	22378 (44.3%)	19596 (38.8%)	65108 (42.9%)
secondary school	17814 (35.2%)	18840 (37.3%)	19942 (39.5%)	56596 (37.3%)
primary school	3974 (7.9%)	4000 (7.9%)	5353 (10.6%)	13327 (8.8%)
professional qualification	5621 (11.1%)	5324 (10.5%)	5652 (11.2%)	16597 (10.9%)
Employment, n (%)				
employed	27103 (53.6%)	28364 (56.1%)	29783 (58.9%)	85250 (56.2%)
retired	23440 (46.4%)	22178 (43.9%)	20760 (41.1%)	66378 (43.8%)
inactive				
Household income, £/year, n (%)	8593 (17.0%)	8092 (16.0%)	9205 (18.2%)	25890 (17.1%)
less than 18000	13012 (25.7%)	12596 (24.9%)	12241 (24.2%)	37849 (25.0%)
18000 to 30999	13808 (27.3%)	13955 (27.6%)	13659 (27.0%)	41422 (27.3%)
31000 to 51999	11486 (22.7%)	11912 (23.6%)	11494 (22.7%)	34892 (23.0%)
52000 to 100000	3644 (7.2%)	3987 (7.9%)	3944 (7.8%)	11575 (7.6%)
greater than 100000				
Townsend deprivation index, n (%)	13001 (25.7%)	13021 (25.8%)	11912 (23.6%)	37934 (25.0%)
first quartile	12861 (25.4%)	12957 (25.6%)	12064 (23.9%)	37882 (25.0%)
second quartile	12650 (25.0%)	12508 (24.7%)	12748 (25.2%)	37906 (25.0%)
third quartile	12031 (23.8%)	12056 (23.9%)	13819 (27.3%)	37906 (25.0%)
fourth quartile				
Smoking status, n (%)	28485 (56.4%)	28586 (56.6%)	28020 (55.4%)	85091 (56.1%)
never	19100 (37.8%)	18721 (37.0%)	17974 (35.6%)	55795 (36.8%)
previous	2958 (5.9%)	3235 (6.4%)	4549 (9.0%)	10742 (7.1%)
current				
Alcohol drinking frequency, n (%)	26469 (52.4%)	26106 (51.7%)	23744 (47.0%)	76319 (50.3%)
>= 3 times/week	21373 (42.3%)	21736 (43.0%)	23280 (46.1%)	66389 (43.8%)
< 3 times/week	2701 (5.3%)	2700 (5.3%)	3519 (7.0%)	8920 (5.9%)
never				
Sleep duration, n (%)	36500 (72.2%)	36547 (72.3%)	34874 (69.0%)	107921 (71.2%)
7-8 h/day	10841 (21.4%)	10736 (21.2%)	12072 (23.9%)	33649 (22.2%)
< 7h/day	3202 (6.3%)	3259 (6.4%)	3597 (7.1%)	10058 (6.6%)
> 8h/day				
Physical activity, n (%)	21842 (43.2%)	19385 (38.4%)	18502 (36.6%)	59729 (39.4%)
high	19780 (39.1%)	20508 (40.6%)	19970 (39.5%)	60258 (39.7%)

moderate	8921 (17.7%)	10649 (21.1%)	12071 (23.9%)	31641 (20.9%)
low				
Frailty index, n (%)	31239 (61.8%)	31511 (62.3%)	30664 (60.7%)	93414 (61.6%)
robust	17201 (34.0%)	16987 (33.6%)	17205 (34.0%)	51393 (33.9%)
pre-frailty	2103 (4.2%)	2044 (4.0%)	2674 (5.3%)	6821 (4.5%)
frailty				
Frailty phenotype, n (%)	33553 (66.4%)	32791 (64.9%)	30353 (60.1%)	96697 (63.8%)
robust	16242 (32.1%)	16912 (33.5%)	18695 (37.0%)	51849 (34.2%)
pre-frailty	748 (1.5%)	839 (1.7%)	1495 (3.0%)	3082 (2.0%)
frailty	23491 (46.5%)	28059 (55.5%)	30336 (60.0%)	81886 (54.0%)
Energy, kcal, mean (SD)	2410 (495)	2010 (396)	1680 (390)	2030 (524)
AHEI, n (%)				
unhealthy tertile	12425 (24.6%)	16895 (33.4%)	21223 (42.0%)	50543 (33.3%)
medium tertile	15660 (31.0%)	17073 (33.8%)	17809 (35.2%)	50542 (33.3%)
healthy tertile	22458 (44.4%)	16574 (32.8%)	11511 (22.8%)	50543 (33.3%)
DASH, n (%)				
unhealthy tertile	13293 (26.3%)	17871 (35.4%)	23113 (45.7%)	54277 (35.8%)
medium tertile	15966 (31.6%)	16636 (32.9%)	16747 (33.1%)	49349 (32.5%)
healthy tertile	21284 (42.1%)	16035 (31.7%)	10683 (21.1%)	48002 (31.7%)
MED, n (%)				
unhealthy tertile	8813 (17.4%)	16289 (32.2%)	28197 (55.8%)	53299 (35.2%)
medium tertile	22339 (44.2%)	23404 (46.3%)	18327 (36.3%)	64070 (42.3%)
healthy tertile	19391 (38.4%)	10849 (21.5%)	4019 (8.0%)	34259 (22.6%)
PDI, n (%)				
unhealthy tertile	13832 (27.4%)	19332 (38.2%)	26373 (52.2%)	59537 (39.3%)
medium tertile	14998 (29.7%)	15588 (30.8%)	14624 (28.9%)	45210 (29.8%)
healthy tertile	21713 (43.0%)	15622 (30.9%)	9546 (18.9%)	46881 (30.9%)
HPDI, n (%)				
unhealthy tertile	16672 (33.0%)	18880 (37.4%)	20199 (40.0%)	55751 (36.8%)
medium tertile	14774 (29.2%)	15395 (30.5%)	16083 (31.8%)	46252 (30.5%)
healthy tertile	19097 (37.8%)	16267 (32.2%)	14261 (28.2%)	49625 (32.7%)
UPDI, n (%)				
healthy tertile	28299 (56.0%)	17206 (34.0%)	8086 (16.0%)	53591 (35.3%)
medium tertile	14577 (28.8%)	17975 (35.6%)	15685 (31.0%)	48237 (31.8%)
unhealthy tertile	7667 (15.2%)	15361 (30.4%)	26772 (53.0%)	49800 (32.8%)
Family history of CVD, n (%)				
no	11785 (23.3%)	11993 (23.7%)	12561 (24.9%)	36339 (24.0%)
yes	38758 (76.7%)	38549 (76.3%)	37982 (75.1%)	115289 (76.0%)
Family history of cancer, n (%)				
no	31615 (62.6%)	31775 (62.9%)	31815 (62.9%)	95205 (62.8%)
yes	18928 (37.4%)	18767 (37.1%)	18728 (37.1%)	56423 (37.2%)
Family history of diabetes, n (%)				
no	40075 (79.3%)	40023 (79.2%)	39667 (78.5%)	119765 (79.0%)
yes	10468 (20.7%)	10519 (20.8%)	10876 (21.5%)	31863 (21.0%)

AHEI, Alternative Healthy Eating Index; DASH, Dietary Approaches to Stop Hypertension; MED, Mediterranean Diet; DII, Dietary Inflammatory Index; PDI, Plant-Based Diet Index; HPDI, Healthy Plant-Based Diet Index; UPDI, Unhealthy Plant-Based Diet Index; CVD, cardiovascular disease; SD: standard deviation. Employment status is categorized as employed (includes paid employment or self- employed,

paid or voluntary work or student), and other (includes retired, looking after home and/or family, unable to work and unemployed). Education is categorized as college or University degree, secondary school (includes A levels/AS levels or equivalent, O levels/GCSEs or equivalent, CSEs or equivalent), primary school, and professional qualification (NVQ or HND or HNC or equivalent, other professional qualifications).

Supplementary Table S12. Baseline characteristics of the participants by PDI

	Unhealthy tertile	Medium Tertile	Healthy Tertile	Overall
	(N=59537)	(N=45210)	(N=46881)	(N=151628)
Age at recruitment, years, mean (SD)	57.6 (6.7)	57.8 (6.7)	57.9 (6.7)	57.8 (6.7)
Sex, n (%)				
female	29820 (50.1%)	24921 (55.1%)	27145 (57.9%)	81886 (54.0%)
male	29717 (49.9%)	20289 (44.9%)	19736 (42.1%)	69742 (46.0%)
Body mass index (BMI), kg/m^2, n (%)				
<25.0	20468 (34.4%)	17028 (37.7%)	18775 (40.0%)	56271 (37.1%)
25.0-29.9	25538 (42.9%)	19146 (42.3%)	19470 (41.5%)	64154 (42.3%)
>=30.0	13531 (22.7%)	9036 (20.0%)	8636 (18.4%)	31203 (20.6%)
Ethnicity, n (%)				
white	57447 (96.5%)	43960 (97.2%)	45511 (97.1%)	146918 (96.9%)
other	2090 (3.5%)	1250 (2.8%)	1370 (2.9%)	4710 (3.1%)
Education, n (%)				
college or University degree	24755 (41.6%)	19475 (43.1%)	20878 (44.5%)	65108 (42.9%)
secondary school	22539 (37.9%)	16964 (37.5%)	17093 (36.5%)	56596 (37.3%)
primary school	5707 (9.6%)	3834 (8.5%)	3786 (8.1%)	13327 (8.8%)
professional qualification	6536 (11.0%)	4937 (10.9%)	5124 (10.9%)	16597 (10.9%)
Employment, n (%)				
employed	34155 (57.4%)	25429 (56.2%)	25666 (54.7%)	85250 (56.2%)
retired	25382 (42.6%)	19781 (43.8%)	21215 (45.3%)	66378 (43.8%)
inactive				
Household income, £/year, n (%)	10321 (17.3%)	7537 (16.7%)	8032 (17.1%)	25890 (17.1%)
less than 18000	14446 (24.3%)	11380 (25.2%)	12023 (25.6%)	37849 (25.0%)
18000 to 30999	16024 (26.9%)	12440 (27.5%)	12958 (27.6%)	41422 (27.3%)
31000 to 51999	13890 (23.3%)	10375 (22.9%)	10627 (22.7%)	34892 (23.0%)
52000 to 100000	4856 (8.2%)	3478 (7.7%)	3241 (6.9%)	11575 (7.6%)
greater than 100000				
Townsend deprivation index, n (%)	14182 (23.8%)	11534 (25.5%)	12218 (26.1%)	37934 (25.0%)
first quartile	14380 (24.2%)	11434 (25.3%)	12068 (25.7%)	37882 (25.0%)
second quartile	14965 (25.1%)	11249 (24.9%)	11692 (24.9%)	37906 (25.0%)
third quartile	16010 (26.9%)	10993 (24.3%)	10903 (23.3%)	37906 (25.0%)
fourth quartile				
Smoking status, n (%)	31836 (53.5%)	25763 (57.0%)	27492 (58.6%)	85091 (56.1%)
never	22567 (37.9%)	16454 (36.4%)	16774 (35.8%)	55795 (36.8%)
previous	5134 (8.6%)	2993 (6.6%)	2615 (5.6%)	10742 (7.1%)
current				
Alcohol drinking frequency, n (%)	31930 (53.6%)	22535 (49.8%)	21854 (46.6%)	76319 (50.3%)
>= 3 times/week	24533 (41.2%)	20055 (44.4%)	21801 (46.5%)	66389 (43.8%)
< 3 times/week	3074 (5.2%)	2620 (5.8%)	3226 (6.9%)	8920 (5.9%)
never				
Sleep duration, n (%)	41880 (70.3%)	32407 (71.7%)	33634 (71.7%)	107921 (71.2%)
7-8 h/day	13508 (22.7%)	9845 (21.8%)	10296 (22.0%)	33649 (22.2%)
< 7h/day	4149 (7.0%)	2958 (6.5%)	2951 (6.3%)	10058 (6.6%)
> 8h/day				
Physical activity, n (%)	22464 (37.7%)	17579 (38.9%)	19686 (42.0%)	59729 (39.4%)
high	23627 (39.7%)	18190 (40.2%)	18441 (39.3%)	60258 (39.7%)

moderate	13446 (22.6%)	9441 (20.9%)	8754 (18.7%)	31641 (20.9%)
low				
Frailty index, n (%)	36406 (61.1%)	28190 (62.4%)	28818 (61.5%)	93414 (61.6%)
robust	20409 (34.3%)	15028 (33.2%)	15956 (34.0%)	51393 (33.9%)
pre-frailty	2722 (4.6%)	1992 (4.4%)	2107 (4.5%)	6821 (4.5%)
frailty				
Frailty phenotype, n (%)	37074 (62.3%)	29075 (64.3%)	30548 (65.2%)	96697 (63.8%)
robust	21078 (35.4%)	15272 (33.8%)	15499 (33.1%)	51849 (34.2%)
pre-frailty	1385 (2.3%)	863 (1.9%)	834 (1.8%)	3082 (2.0%)
frailty	29820 (50.1%)	24921 (55.1%)	27145 (57.9%)	81886 (54.0%)
Energy, kcal, mean (SD)	1940 (527)	2030 (509)	2150 (511)	2030 (524)
AHEI, n (%)				
unhealthy tertile	28558 (48.0%)	14127 (31.2%)	7858 (16.8%)	50543 (33.3%)
medium tertile	19246 (32.3%)	16015 (35.4%)	15281 (32.6%)	50542 (33.3%)
healthy tertile	11733 (19.7%)	15068 (33.3%)	23742 (50.6%)	50543 (33.3%)
DASH, n (%)				
unhealthy tertile	31418 (52.8%)	14831 (32.8%)	8028 (17.1%)	54277 (35.8%)
medium tertile	18411 (30.9%)	15900 (35.2%)	15038 (32.1%)	49349 (32.5%)
healthy tertile	9708 (16.3%)	14479 (32.0%)	23815 (50.8%)	48002 (31.7%)
MED, n (%)				
unhealthy tertile	31011 (52.1%)	14596 (32.3%)	7692 (16.4%)	53299 (35.2%)
medium tertile	22472 (37.7%)	20423 (45.2%)	21175 (45.2%)	64070 (42.3%)
healthy tertile	6054 (10.2%)	10191 (22.5%)	18014 (38.4%)	34259 (22.6%)
DII, n (%)				
healthy tertile	13832 (23.2%)	14998 (33.2%)	21713 (46.3%)	50543 (33.3%)
medium tertile	19332 (32.5%)	15588 (34.5%)	15622 (33.3%)	50542 (33.3%)
unhealthy tertile	26373 (44.3%)	14624 (32.3%)	9546 (20.4%)	50543 (33.3%)
HPDI, n (%)				
unhealthy tertile	29705 (49.9%)	15975 (35.3%)	10071 (21.5%)	55751 (36.8%)
medium tertile	18712 (31.4%)	14199 (31.4%)	13341 (28.5%)	46252 (30.5%)
healthy tertile	11120 (18.7%)	15036 (33.3%)	23469 (50.1%)	49625 (32.7%)
UPDI, n (%)				
healthy tertile	20985 (35.2%)	15694 (34.7%)	16912 (36.1%)	53591 (35.3%)
medium tertile	19672 (33.0%)	14025 (31.0%)	14540 (31.0%)	48237 (31.8%)
unhealthy tertile	18880 (31.7%)	15491 (34.3%)	15429 (32.9%)	49800 (32.8%)
Family history of CVD, n (%)				
no	14811 (24.9%)	10838 (24.0%)	10690 (22.8%)	36339 (24.0%)
yes	44726 (75.1%)	34372 (76.0%)	36191 (77.2%)	115289 (76.0%)
Family history of cancer, n (%)				
no	37328 (62.7%)	28295 (62.6%)	29582 (63.1%)	95205 (62.8%)
yes	22209 (37.3%)	16915 (37.4%)	17299 (36.9%)	56423 (37.2%)
Family history of diabetes, n (%)				
no	46950 (78.9%)	35735 (79.0%)	37080 (79.1%)	119765 (79.0%)
yes	12587 (21.1%)	9475 (21.0%)	9801 (20.9%)	31863 (21.0%)

AHEI, Alternative Healthy Eating Index; DASH, Dietary Approaches to Stop Hypertension; MED, Mediterranean Diet; DII, Dietary Inflammatory Index; PDI, Plant-Based Diet Index; HPDI, Healthy Plant-Based Diet Index; UPDI, Unhealthy Plant-Based Diet Index; CVD, cardiovascular disease; SD: standard deviation. Employment status is categorized as employed (includes paid employment or self- employed,

paid or voluntary work or student), and other (includes retired, looking after home and/or family, unable to work and unemployed). Education is categorized as college or University degree, secondary school (includes A levels/AS levels or equivalent, O levels/GCSEs or equivalent, CSEs or equivalent), primary school, and professional qualification (NVQ or HND or HNC or equivalent, other professional qualifications).

Supplementary Table S13. Baseline characteristics of the participants by HPDI

	**Unhealthy tertile**	**Medium Tertile**	**Healthy Tertile**	**Overall**
	**(N=55751)**	**(N=46252)**	**(N=49625)**	**(N=151628)**
Age at recruitment, years, mean (SD)	57.3 (6.8)	58.0 (6.6)	58.1 (6.5)	57.8 (6.7)
Sex, n (%)				
female	23514 (42.2%)	25503 (55.1%)	32869 (66.2%)	81886 (54.0%)
male	32237 (57.8%)	20749 (44.9%)	16756 (33.8%)	69742 (46.0%)
Body mass index (BMI), kg/m^2, n (%)				
<25.0	17327 (31.1%)	17398 (37.6%)	21546 (43.4%)	56271 (37.1%)
25.0-29.9	24530 (44.0%)	19753 (42.7%)	19871 (40.0%)	64154 (42.3%)
>=30.0	13894 (24.9%)	9101 (19.7%)	8208 (16.5%)	31203 (20.6%)
Ethnicity, n (%)				
white	54231 (97.3%)	44836 (96.9%)	47851 (96.4%)	146918 (96.9%)
other	1520 (2.7%)	1416 (3.1%)	1774 (3.6%)	4710 (3.1%)
Education, n (%)				
college or University degree	21889 (39.3%)	19907 (43.0%)	23312 (47.0%)	65108 (42.9%)
secondary school	22038 (39.5%)	17262 (37.3%)	17296 (34.9%)	56596 (37.3%)
primary school	5377 (9.6%)	4128 (8.9%)	3822 (7.7%)	13327 (8.8%)
professional qualification	6447 (11.6%)	4955 (10.7%)	5195 (10.5%)	16597 (10.9%)
Employment, n (%)				
employed	32367 (58.1%)	25549 (55.2%)	27334 (55.1%)	85250 (56.2%)
retired	23384 (41.9%)	20703 (44.8%)	22291 (44.9%)	66378 (43.8%)
inactive				
Household income, £/year, n (%)	9668 (17.3%)	7852 (17.0%)	8370 (16.9%)	25890 (17.1%)
less than 18000	13787 (24.7%)	11605 (25.1%)	12457 (25.1%)	37849 (25.0%)
18000 to 30999	15526 (27.8%)	12447 (26.9%)	13449 (27.1%)	41422 (27.3%)
31000 to 51999	12737 (22.8%)	10760 (23.3%)	11395 (23.0%)	34892 (23.0%)
52000 to 100000	4033 (7.2%)	3588 (7.8%)	3954 (8.0%)	11575 (7.6%)
greater than 100000				
Townsend deprivation index, n (%)	13841 (24.8%)	11761 (25.4%)	12332 (24.9%)	37934 (25.0%)
first quartile	13845 (24.8%)	11767 (25.4%)	12270 (24.7%)	37882 (25.0%)
second quartile	13993 (25.1%)	11520 (24.9%)	12393 (25.0%)	37906 (25.0%)
third quartile	14072 (25.2%)	11204 (24.2%)	12630 (25.5%)	37906 (25.0%)
fourth quartile				
Smoking status, n (%)	30738 (55.1%)	26058 (56.3%)	28295 (57.0%)	85091 (56.1%)
never	20292 (36.4%)	16970 (36.7%)	18533 (37.3%)	55795 (36.8%)
previous	4721 (8.5%)	3224 (7.0%)	2797 (5.6%)	10742 (7.1%)
current				
Alcohol drinking frequency, n (%)	28136 (50.5%)	23914 (51.7%)	24269 (48.9%)	76319 (50.3%)
>= 3 times/week	24461 (43.9%)	19775 (42.8%)	22153 (44.6%)	66389 (43.8%)
< 3 times/week	3154 (5.7%)	2563 (5.5%)	3203 (6.5%)	8920 (5.9%)
never				
Sleep duration, n (%)	39222 (70.4%)	33190 (71.8%)	35509 (71.6%)	107921 (71.2%)
7-8 h/day	12646 (22.7%)	10031 (21.7%)	10972 (22.1%)	33649 (22.2%)
< 7h/day	3883 (7.0%)	3031 (6.6%)	3144 (6.3%)	10058 (6.6%)
> 8h/day				
Physical activity, n (%)	20336 (36.5%)	18083 (39.1%)	21310 (42.9%)	59729 (39.4%)
high	22143 (39.7%)	18578 (40.2%)	19537 (39.4%)	60258 (39.7%)

moderate	13272 (23.8%)	9591 (20.7%)	8778 (17.7%)	31641 (20.9%)
low				
Frailty index, n (%)	33120 (59.4%)	28673 (62.0%)	31621 (63.7%)	93414 (61.6%)
robust	19710 (35.4%)	15595 (33.7%)	16088 (32.4%)	51393 (33.9%)
pre-frailty	2921 (5.2%)	1984 (4.3%)	1916 (3.9%)	6821 (4.5%)
frailty				
Frailty phenotype, n (%)	34791 (62.4%)	29682 (64.2%)	32224 (64.9%)	96697 (63.8%)
robust	19572 (35.1%)	15670 (33.9%)	16607 (33.5%)	51849 (34.2%)
pre-frailty	1388 (2.5%)	900 (1.9%)	794 (1.6%)	3082 (2.0%)
frailty	23514 (42.2%)	25503 (55.1%)	32869 (66.2%)	81886 (54.0%)
Energy, kcal, mean (SD)	2200 (520)	2010 (496)	1870 (494)	2030 (524)
AHEI, n (%)				
unhealthy tertile	35132 (63.0%)	12103 (26.2%)	3308 (6.7%)	50543 (33.3%)
medium tertile	17368 (31.2%)	20970 (45.3%)	12204 (24.6%)	50542 (33.3%)
healthy tertile	3251 (5.8%)	13179 (28.5%)	34113 (68.7%)	50543 (33.3%)
DASH, n (%)				
unhealthy tertile	39416 (70.7%)	12484 (27.0%)	2377 (4.8%)	54277 (35.8%)
medium tertile	14192 (25.5%)	22277 (48.2%)	12880 (26.0%)	49349 (32.5%)
healthy tertile	2143 (3.8%)	11491 (24.8%)	34368 (69.3%)	48002 (31.7%)
MED, n (%)				
unhealthy tertile	29729 (53.3%)	15393 (33.3%)	8177 (16.5%)	53299 (35.2%)
medium tertile	21073 (37.8%)	21286 (46.0%)	21711 (43.8%)	64070 (42.3%)
healthy tertile	4949 (8.9%)	9573 (20.7%)	19737 (39.8%)	34259 (22.6%)
DII, n (%)				
healthy tertile	16672 (29.9%)	14774 (31.9%)	19097 (38.5%)	50543 (33.3%)
medium tertile	18880 (33.9%)	15395 (33.3%)	16267 (32.8%)	50542 (33.3%)
unhealthy tertile	20199 (36.2%)	16083 (34.8%)	14261 (28.7%)	50543 (33.3%)
PDI, n (%)				
unhealthy tertile	29705 (53.3%)	18712 (40.5%)	11120 (22.4%)	59537 (39.3%)
medium tertile	15975 (28.7%)	14199 (30.7%)	15036 (30.3%)	45210 (29.8%)
healthy tertile	10071 (18.1%)	13341 (28.8%)	23469 (47.3%)	46881 (30.9%)
UPDI, n (%)				
healthy tertile	8408 (15.1%)	15420 (33.3%)	29763 (60.0%)	53591 (35.3%)
medium tertile	16847 (30.2%)	17357 (37.5%)	14033 (28.3%)	48237 (31.8%)
unhealthy tertile	30496 (54.7%)	13475 (29.1%)	5829 (11.7%)	49800 (32.8%)
Family history of CVD, n (%)				
no	13827 (24.8%)	11193 (24.2%)	11319 (22.8%)	36339 (24.0%)
yes	41924 (75.2%)	35059 (75.8%)	38306 (77.2%)	115289 (76.0%)
Family history of cancer, n (%)				
no	35078 (62.9%)	28991 (62.7%)	31136 (62.7%)	95205 (62.8%)
yes	20673 (37.1%)	17261 (37.3%)	18489 (37.3%)	56423 (37.2%)
Family history of diabetes, n (%)				
no	43744 (78.5%)	36641 (79.2%)	39380 (79.4%)	119765 (79.0%)
yes	12007 (21.5%)	9611 (20.8%)	10245 (20.6%)	31863 (21.0%)

AHEI, Alternative Healthy Eating Index; DASH, Dietary Approaches to Stop Hypertension; MED, Mediterranean Diet; DII, Dietary Inflammatory Index; PDI, Plant-Based Diet Index; HPDI, Healthy Plant-Based Diet Index; UPDI, Unhealthy Plant-Based Diet Index; CVD, cardiovascular disease; SD: standard deviation. Employment status is categorized as employed (includes paid employment or self- employed,

paid or voluntary work or student), and other (includes retired, looking after home and/or family, unable to work and unemployed). Education is categorized as college or University degree, secondary school (includes A levels/AS levels or equivalent, O levels/GCSEs or equivalent, CSEs or equivalent), primary school, and professional qualification (NVQ or HND or HNC or equivalent, other professional qualifications).

Supplementary Table S14. Baseline characteristics of the participants by UPDI

	Healthy Tertile	Medium Tertile	Unhealthy Tertile	Overall
	(N=53591)	(N=48237)	(N=49800)	(N=151628)
Age at recruitment, years, mean (SD)	58.7 (6.4)	57.9 (6.6)	56.7 (6.9)	57.8 (6.7)
Sex, n (%)				
female	32087 (59.9%)	25832 (53.6%)	23967 (48.1%)	81886 (54.0%)
male	21504 (40.1%)	22405 (46.4%)	25833 (51.9%)	69742 (46.0%)
Body mass index (BMI), kg/m^2, n (%)				
<25.0	21733 (40.6%)	18149 (37.6%)	16389 (32.9%)	56271 (37.1%)
25.0-29.9	22011 (41.1%)	20391 (42.3%)	21752 (43.7%)	64154 (42.3%)
>=30.0	9847 (18.4%)	9697 (20.1%)	11659 (23.4%)	31203 (20.6%)
Ethnicity, n (%)				
white	52365 (97.7%)	46831 (97.1%)	47722 (95.8%)	146918 (96.9%)
other	1226 (2.3%)	1406 (2.9%)	2078 (4.2%)	4710 (3.1%)
Education, n (%)				
college or University degree	25359 (47.3%)	21050 (43.6%)	18699 (37.5%)	65108 (42.9%)
secondary school	18650 (34.8%)	17658 (36.6%)	20288 (40.7%)	56596 (37.3%)
primary school	3922 (7.3%)	4294 (8.9%)	5111 (10.3%)	13327 (8.8%)
professional qualification	5660 (10.6%)	5235 (10.9%)	5702 (11.4%)	16597 (10.9%)
Employment, n (%)				
employed	27804 (51.9%)	27032 (56.0%)	30414 (61.1%)	85250 (56.2%)
retired	25787 (48.1%)	21205 (44.0%)	19386 (38.9%)	66378 (43.8%)
inactive				
Household income, £/year, n (%)	9003 (16.8%)	8170 (16.9%)	8717 (17.5%)	25890 (17.1%)
less than 18000	13832 (25.8%)	12016 (24.9%)	12001 (24.1%)	37849 (25.0%)
18000 to 30999	14684 (27.4%)	13051 (27.1%)	13687 (27.5%)	41422 (27.3%)
31000 to 51999	12015 (22.4%)	11283 (23.4%)	11594 (23.3%)	34892 (23.0%)
52000 to 100000	4057 (7.6%)	3717 (7.7%)	3801 (7.6%)	11575 (7.6%)
greater than 100000				
Townsend deprivation index, n (%)	13705 (25.6%)	12256 (25.4%)	11973 (24.0%)	37934 (25.0%)
first quartile	13683 (25.5%)	12046 (25.0%)	12153 (24.4%)	37882 (25.0%)
second quartile	13372 (25.0%)	12107 (25.1%)	12427 (25.0%)	37906 (25.0%)
third quartile	12831 (23.9%)	11828 (24.5%)	13247 (26.6%)	37906 (25.0%)
fourth quartile				
Smoking status, n (%)	30041 (56.1%)	26956 (55.9%)	28094 (56.4%)	85091 (56.1%)
never	20638 (38.5%)	17896 (37.1%)	17261 (34.7%)	55795 (36.8%)
previous	2912 (5.4%)	3385 (7.0%)	4445 (8.9%)	10742 (7.1%)
current				
Alcohol drinking frequency, n (%)	27266 (50.9%)	24844 (51.5%)	24209 (48.6%)	76319 (50.3%)
>= 3 times/week	23404 (43.7%)	20694 (42.9%)	22291 (44.8%)	66389 (43.8%)
< 3 times/week	2921 (5.5%)	2699 (5.6%)	3300 (6.6%)	8920 (5.9%)
never				
Sleep duration, n (%)	38690 (72.2%)	34648 (71.8%)	34583 (69.4%)	107921 (71.2%)
7-8 h/day	11559 (21.6%)	10368 (21.5%)	11722 (23.5%)	33649 (22.2%)
< 7h/day	3342 (6.2%)	3221 (6.7%)	3495 (7.0%)	10058 (6.6%)
> 8h/day				
Physical activity, n (%)	22851 (42.6%)	18834 (39.0%)	18044 (36.2%)	59729 (39.4%)
high	21283 (39.7%)	19446 (40.3%)	19529 (39.2%)	60258 (39.7%)

moderate	9457 (17.6%)	9957 (20.6%)	12227 (24.6%)	31641 (20.9%)
low				
Frailty index, n (%)	33718 (62.9%)	29984 (62.2%)	29712 (59.7%)	93414 (61.6%)
robust	17835 (33.3%)	16160 (33.5%)	17398 (34.9%)	51393 (33.9%)
pre-frailty	2038 (3.8%)	2093 (4.3%)	2690 (5.4%)	6821 (4.5%)
frailty				
Frailty phenotype, n (%)	35286 (65.8%)	31033 (64.3%)	30378 (61.0%)	96697 (63.8%)
robust	17548 (32.7%)	16297 (33.8%)	18004 (36.2%)	51849 (34.2%)
pre-frailty	757 (1.4%)	907 (1.9%)	1418 (2.8%)	3082 (2.0%)
frailty	32087 (59.9%)	25832 (53.6%)	23967 (48.1%)	81886 (54.0%)
Energy, kcal, mean (SD)	2090 (514)	2020 (519)	1980 (533)	2030 (524)
AHEI, n (%)				
unhealthy tertile	8169 (15.2%)	15411 (31.9%)	26963 (54.1%)	50543 (33.3%)
medium tertile	15793 (29.5%)	18015 (37.3%)	16734 (33.6%)	50542 (33.3%)
healthy tertile	29629 (55.3%)	14811 (30.7%)	6103 (12.3%)	50543 (33.3%)
DASH, n (%)				
unhealthy tertile	7616 (14.2%)	16142 (33.5%)	30519 (61.3%)	54277 (35.8%)
medium tertile	16060 (30.0%)	18410 (38.2%)	14879 (29.9%)	49349 (32.5%)
healthy tertile	29915 (55.8%)	13685 (28.4%)	4402 (8.8%)	48002 (31.7%)
MED, n (%)				
unhealthy tertile	10371 (19.4%)	16752 (34.7%)	26176 (52.6%)	53299 (35.2%)
medium tertile	23876 (44.6%)	21491 (44.6%)	18703 (37.6%)	64070 (42.3%)
healthy tertile	19344 (36.1%)	9994 (20.7%)	4921 (9.9%)	34259 (22.6%)
DII, n (%)				
healthy tertile	28299 (52.8%)	14577 (30.2%)	7667 (15.4%)	50543 (33.3%)
medium tertile	17206 (32.1%)	17975 (37.3%)	15361 (30.8%)	50542 (33.3%)
unhealthy tertile	8086 (15.1%)	15685 (32.5%)	26772 (53.8%)	50543 (33.3%)
PDI, n (%)				
unhealthy tertile	20985 (39.2%)	19672 (40.8%)	18880 (37.9%)	59537 (39.3%)
medium tertile	15694 (29.3%)	14025 (29.1%)	15491 (31.1%)	45210 (29.8%)
healthy tertile	16912 (31.6%)	14540 (30.1%)	15429 (31.0%)	46881 (30.9%)
HPDI, n (%)				
unhealthy tertile	8408 (15.7%)	16847 (34.9%)	30496 (61.2%)	55751 (36.8%)
medium tertile	15420 (28.8%)	17357 (36.0%)	13475 (27.1%)	46252 (30.5%)
healthy tertile	29763 (55.5%)	14033 (29.1%)	5829 (11.7%)	49625 (32.7%)
Family history of CVD, n (%)				
no	12269 (22.9%)	11554 (24.0%)	12516 (25.1%)	36339 (24.0%)
yes	41322 (77.1%)	36683 (76.0%)	37284 (74.9%)	115289 (76.0%)
Family history of cancer, n (%)				
no	33285 (62.1%)	30168 (62.5%)	31752 (63.8%)	95205 (62.8%)
yes	20306 (37.9%)	18069 (37.5%)	18048 (36.2%)	56423 (37.2%)
Family history of diabetes, n (%)				
no	42693 (79.7%)	38188 (79.2%)	38884 (78.1%)	119765 (79.0%)
yes	10898 (20.3%)	10049 (20.8%)	10916 (21.9%)	31863 (21.0%)

AHEI, Alternative Healthy Eating Index; DASH, Dietary Approaches to Stop Hypertension; MED, Mediterranean Diet; DII, Dietary Inflammatory Index; PDI, Plant-Based Diet Index; HPDI, Healthy Plant-Based Diet Index; UPDI, Unhealthy Plant-Based Diet Index; CVD, cardiovascular disease; SD: standard deviation. Employment status is categorized as employed (includes paid employment or self- employed,

paid or voluntary work or student), and other (includes retired, looking after home and/or family, unable to work and unemployed). Education is categorized as college or University degree, secondary school (includes A levels/AS levels or equivalent, O levels/GCSEs or equivalent, CSEs or equivalent), primary school, and professional qualification (NVQ or HND or HNC or equivalent, other professional qualifications).

Supplementary Table S15. Analyses on interaction of diet quality scores and pre-frailty with all-cause mortality

	Robust	Pre-frailty	Multiplicative interaction	Additive interaction		
	HR (95%CI)	HR (95%CI)	HR (95%CI)	RERI	AP	S
**Frailty index**						
**AHEI**						
Upper 40%	Ref.	1.28 (1.19,1.38)	1.02 (0.93,1.12)	0.05 (-0.06,0.17)	0.04 (-0.04,0.12)	1.15 (0.84,1.56)
Lower 60%	1.09 (1.02,1.16)	1.42 (1.33,1.52)				
**DASH**						
Upper 40%	Ref.	1.25 (1.16,1.35)	1.06 (0.96,1.16)	0.10 (-0.01,0.22)	0.07 (-0.01,0.15)	1.29 (0.94,1.78)
Lower 60%	1.11 (1.04,1.18)	1.46 (1.37,1.56)				
**MED**						
Upper 40%	Ref.	1.22 (1.10,1.35)	1.09 (0.97,1.22)	0.14 (0.01,0.27)	0.10 (0.01,0.18)	1.43 (0.94,2.16)
Lower 60%	1.12 (1.04,1.20)	1.47 (1.36,1.59)				
**DII**						
Lower 60%	Ref.	1.27 (1.20,1.35)	1.05 (0.95,1.15)	0.10 (-0.02,0.21)	0.07 (-0.01,0.14)	1.25 (0.94,1.66)
Upper 40%	1.11 (1.04,1.19)	1.49 (1.38,1.60)				
**PDI**						
Upper 40%	Ref.	1.32 (1.22,1.42)	0.98 (0.89,1.08)	0.00 (-0.12,0.11)	0.00 (-0.09,0.08)	0.99 (0.74,1.32)
Lower 60%	1.09 (1.02,1.16)	1.40 (1.31,1.50)				
**HPDI**						
Upper 40%	Ref.	1.27 (1.18,1.38)	1.03 (0.94,1.13)	0.06 (-0.05,0.17)	0.04 (-0.04,0.12)	1.17 (0.84,1.64)
Lower 60%	1.07 (1.00,1.14)	1.41 (1.31,1.50)				
**UPDI**						
Lower 60%	Ref.	1.22 (1.15,1.30)	1.15 (1.05,1.27)	0.23 (0.12,0.35)	0.15 (0.08,0.22)	1.71 (1.24,2.37)
Upper 40%	1.11 (1.04,1.18)	1.56 (1.46,1.66)				
**Frailty phenotype**						
**AHEI**						
Upper 40%	Ref.	1.27 (1.17,1.37)	1.06 (0.97,1.17)	0.10 (-0.01,0.21)	0.07 (-0.01,0.15)	1.30 (0.93,1.82)
Lower 60%	1.07 (1.00,1.13)	1.43 (1.34,1.53)				
**DASH**						
Upper 40%	Ref.	1.23 (1.14,1.32)	1.11 (1.01,1.22)	0.17 (0.06,0.28)	0.11 (0.04,0.19)	1.55 (1.07,2.24)
Lower 60%	1.08 (1.01,1.15)	1.47 (1.38,1.57)				
**MED**						
Upper 40%	Ref.	1.24 (1.12,1.37)	1.08 (0.96,1.21)	0.14 (0.01,0.27)	0.09 (0.00,0.18)	1.39 (0.94,2.05)
Lower 60%	1.11 (1.04,1.19)	1.49 (1.38,1.60)				
**DII**						
Lower 60%	Ref.	1.29 (1.22,1.37)	1.05 (0.95,1.15)	0.09 (-0.02,0.21)	0.06 (-0.01,0.14)	1.24 (0.94,1.64)
Upper 40%	1.10 (1.03,1.17)	1.48 (1.38,1.59)				
**PDI**						
Upper 40%	Ref.	1.27 (1.18,1.38)	1.05 (0.96,1.15)	0.08 (-0.03,0.19)	0.06 (-0.02,0.14)	1.25 (0.88,1.79)
Lower 60%	1.04 (0.98,1.10)	1.39 (1.30,1.49)				
**HPDI**						
Upper 40%	Ref.	1.28 (1.19,1.39)	1.04 (0.95,1.14)	0.07 (-0.04,0.18)	0.05 (-0.03,0.13)	1.21 (0.86,1.69)
Lower 60%	1.05 (0.99,1.12)	1.41 (1.31,1.50)				
**UPDI**						
Lower 60%	Ref.	1.27 (1.20,1.35)	1.09 (0.99,1.19)	0.16 (0.04,0.27)	0.10 (0.03,0.17)	1.40 (1.06,1.84)
Upper 40%	1.12 (1.05,1.19)	1.55 (1.45,1.65)				

Model was adjusted for age at recruitment (strata), sex, assessment center (strata), body mass index, ethnicity, education, employment, household income, Townsend deprivation index, smoking status, alcohol drinking frequency, physical activity, energy, sleep duration, family history of diabetes, family history of CVD, and family history of cancer. AHEI, Alternative Healthy Eating Index; DASH, Dietary Approaches to Stop Hypertension; MED, Mediterranean Diet; DII, Dietary Inflammatory Index; PDI, Plant-Based Diet Index; HPDI, Healthy Plant-Based Diet Index; UPDI, Unhealthy Plant-Based Diet Index; HR, hazard ratios; CI, confidence interval; RERI, relative excess risk due to interaction; AP, attributable proportion due to interaction; S, the synergy index.

Supplementary Table S16. Analyses on interaction of diet quality scores and frailty with all-cause mortality

	Robust	Frailty	Multiplicative interaction	Additive interaction		
	HR (95%CI)	HR (95%CI)	HR (95%CI)	RERI	AP	S
**Frailty index**						
**AHEI**						
Upper 40%	Ref.	1.72 (1.50,1.98)	1.06 (0.90,1.25)	0.20 (-0.09,0.48)	0.10 (-0.04,0.23)	1.24 (0.89,1.71)
Lower 60%	1.11 (1.04,1.18)	2.03 (1.82,2.26)				
**DASH**						
Upper 40%	Ref.	1.66 (1.43,1.93)	1.11 (0.94,1.32)	0.29 (0.00,0.58)	0.14 (0.01,0.27)	1.37 (0.97,1.93)
Lower 60%	1.12 (1.05,1.20)	2.08 (1.86,2.31)				
**MED**						
Upper 40%	Ref.	1.83 (1.50,2.22)	0.98 (0.79,1.21)	0.05 (-0.32,0.42)	0.03 (-0.16,0.21)	1.06 (0.71,1.56)
Lower 60%	1.11 (1.03,1.20)	1.99 (1.78,2.23)				
**DII**						
Lower 60%	Ref.	1.78 (1.59,1.99)	1.02 (0.87,1.19)	0.12 (-0.16,0.40)	0.06 (-0.08,0.20)	1.14 (0.84,1.54)
Upper 40%	1.10 (1.03,1.19)	2.00 (1.76,2.27)				
**PDI**						
Upper 40%	Ref.	1.73 (1.50,1.99)	1.07 (0.90,1.26)	0.19 (-0.10,0.47)	0.09 (-0.04,0.23)	1.23 (0.89,1.71)
Lower 60%	1.09 (1.02,1.16)	2.00 (1.80,2.24)				
**HPDI**						
Upper 40%	Ref.	1.76 (1.53,2.04)	1.02 (0.86,1.21)	0.11 (-0.18,0.40)	0.06 (-0.09,0.20)	1.13 (0.81,1.58)
Lower 60%	1.08 (1.01,1.16)	1.96 (1.76,2.19)				
**UPDI**						
Lower 60%	Ref.	1.74 (1.55,1.95)	1.06 (0.90,1.24)	0.19 (-0.09,0.47)	0.09 (-0.04,0.22)	1.23 (0.91,1.65)
Upper 40%	1.11 (1.04,1.19)	2.05 (1.82,2.30)				
**Frailty phenotype**						
**AHEI**						
Upper 40%	Ref.	2.11 (1.75,2.55)	1.25 (1.00,1.55)	0.64 (0.15,1.12)	0.23 (0.07,0.38)	1.54 (1.07,2.21)
Lower 60%	1.07 (1.01,1.14)	2.82 (2.47,3.22)				
**DASH**						
Upper 40%	Ref.	2.03 (1.66,2.49)	1.29 (1.03,1.63)	0.74 (0.24,1.24)	0.26 (0.10,0.42)	1.66 (1.12,2.46)
Lower 60%	1.09 (1.02,1.16)	2.86 (2.52,3.26)				
**MED**						
Upper 40%	Ref.	2.45 (1.88,3.21)	1.00 (0.76,1.33)	0.19 (-0.50,0.88)	0.07 (-0.18,0.31)	1.12 (0.73,1.72)
Lower 60%	1.12 (1.04,1.20)	2.76 (2.42,3.15)				
**DII**						
Lower 60%	Ref.	2.37 (2.03,2.77)	1.07 (0.88,1.30)	0.29 (-0.19,0.78)	0.11 (-0.06,0.27)	1.20 (0.89,1.63)
Upper 40%	1.09 (1.01,1.17)	2.75 (2.38,3.18)				
**PDI**						
Upper 40%	Ref.	1.99 (1.63,2.43)	1.35 (1.08,1.69)	0.76 (0.28,1.24)	0.27 (0.12,0.43)	1.74 (1.16,2.60)
Lower 60%	1.04 (0.97,1.10)	2.79 (2.45,3.17)				
**HPDI**						
Upper 40%	Ref.	1.98 (1.61,2.44)	1.34 (1.06,1.69)	0.78 (0.28,1.28)	0.28 (0.12,0.44)	1.74 (1.15,2.64)
Lower 60%	1.07 (1.00,1.14)	2.82 (2.48,3.21)				
**UPDI**						
Lower 60%	Ref.	2.19 (1.87,2.56)	1.22 (1.00,1.49)	0.71 (0.22,1.20)	0.23 (0.09,0.38)	1.54 (1.13,2.08)
Upper 40%	1.13 (1.06,1.20)	3.03 (2.63,3.48)				

Model was adjusted for age at recruitment (strata), sex, assessment center (strata), body mass index, ethnicity, education, employment, household income, Townsend deprivation index, smoking status, alcohol drinking frequency, physical activity, energy, sleep duration, family history of diabetes, family history of CVD, and family history of cancer. AHEI, Alternative Healthy Eating Index; DASH, Dietary Approaches to Stop Hypertension; MED, Mediterranean Diet; DII, Dietary Inflammatory Index; PDI, Plant-Based Diet Index; HPDI, Healthy Plant-Based Diet Index; UPDI, Unhealthy Plant-Based Diet Index; HR, hazard ratios; CI, confidence interval; RERI, relative excess risk due to interaction; AP, attributable proportion due to interaction; S, the synergy index.

Supplementary Table S17. Association between frailty status and all-cause mortality

	Frailty index					Frailty phenotype				
	Case/N	Person year	HR (95% CI)	AIC	BIC	Case/N	Person year	HR (95%CI)	AIC	BIC
**Model 1**				134395.7	134437.8				134315.3	134357.4
Robust	4046/93414	1142108	Ref.			4386/96697	1183628	Ref.		
Pre-frailty	3431/51393	621493	1.41(1.34-1.47)			3397/51849	625084	1.42(1.36-1.49)		
Frailty	754/6821	80932	2.26(2.09-2.45)			448/3082	35821	3.17(2.87-3.5)		
P for trend			<0.001					<0.001		
**Model 2**				133581.6	133785.0				133504.0	133707.5
Robust	4046/93414	1142108	Ref.			4386/96697	1183628	Ref.		
Pre-frailty	3431/51393	621493	1.30(1.24-1.36)			3397/51849	625084	1.32(1.26-1.39)		
Frailty	754/6821	80932	1.77(1.63-1.93)			448/3082	35821	2.37(2.14-2.63)		
P for trend			<0.001					<0.001		
**Model 2 + AHEI**				133561.2	133778.7				133484.5	133702.0
Robust	4046/93414	1142108	Ref.			4386/96697	1183628	Ref.		
Pre-frailty	3431/51393	621493	1.29(1.23-1.35)			3397/51849	625084	1.32(1.26-1.38)		
Frailty	754/6821	80932	1.77(1.63-1.92)			448/3082	35821	2.36(2.13-2.62)		
P for trend			<0.001					<0.001		
**Model 2 + DASH**				133550.5	133768.0				133473.9	133691.4
Robust	4046/93414	1142108	Ref.			4386/96697	1183628	Ref.		
Pre-frailty	3431/51393	621493	1.29(1.23-1.35)			3397/51849	625084	1.32(1.26-1.38)		
Frailty	754/6821	80932	1.76(1.62-1.92)			448/3082	35821	2.35(2.12-2.61)		
P for trend			<0.001					<0.001		
**Model 2 + MED**				133546.0	133763.5				133470.0	133687.4
Robust	4046/93414	1142108	Ref.			4386/96697	1183628	Ref.		
Pre-frailty	3431/51393	621493	1.29(1.23-1.35)			3397/51849	625084	1.32(1.26-1.38)		
Frailty	754/6821	80932	1.77(1.62-1.92)			448/3082	35821	2.35(2.12-2.61)		
P for trend			<0.001					<0.001		
**Model 2 + DII**				133560.4	133777.9				133485.4	133702.9
Robust	4046/93414	1142108	Ref.			4386/96697	1183628	Ref.		
Pre-frailty	3431/51393	621493	1.29(1.23-1.36)			3397/51849	625084	1.32(1.26-1.38)		

Frailty	754/6821	80932	1.77(1.63-1.92)			448/3082	35821	2.35(2.12-2.61)		
P for trend			<0.001					<0.001		
**Model 2 + PDI**				133569.3	133786.8				133493.7	133711.2
Robust	4046/93414	1142108	Ref.			4386/96697	1183628	Ref.		
Pre-frailty	3431/51393	621493	1.29(1.24-1.36)			3397/51849	625084	1.32(1.26-1.38)		
Frailty	754/6821	80932	1.78(1.63-1.93)			448/3082	35821	2.37(2.13-2.63)		
P for trend			<0.001					<0.001		
**Model 2 + HPDI**				133567.8	133785.3				133491.2	133708.6
Robust	4046/93414	1142108	Ref.			4386/96697	1183628	Ref.		
Pre-frailty	3431/51393	621493	1.29(1.23-1.36)			3397/51849	625084	1.32(1.26-1.38)		
Frailty	754/6821	80932	1.77(1.63-1.92)			448/3082	35821	2.36(2.12-2.62)		
P for trend			<0.001					<0.001		
**Model 2 + UPDI**				133531.3	133748.7				133456.0	133673.5
Robust	4046/93414	1142108	Ref.			4386/96697	1183628	Ref.		
Pre-frailty	3431/51393	621493	1.29(1.23-1.35)			3397/51849	625084	1.32(1.26-1.38)		
Frailty	754/6821	80932	1.76(1.62-1.91)			448/3082	35821	2.34(2.11-2.59)		
P for trend			<0.001					<0.001		

Model 1: adjusted for age at recruitment (strata), sex, assessment center (strata), body mass index, and ethnicity. Model 2: further adjusted for education, employment, household income, Townsend deprivation index, smoking status, alcohol drinking frequency, sleep duration, physical activity, energy, family history of diabetes, family history of CVD, and family history of cancer. HR, hazard ratios; CI, confidence interval; SD: standard deviation; AIC, Akaike information criterion; BIC, Bayesian information criterion; CVD, cardiovascular diseases

Supplementary Table S18. Association between diet quality scores and all-cause mortality

			Model 1	Model 2	Model 2+ frailty index	Model 2 + Frailty phenotype
	Events/N	Person year	HR (95% CI)	HR (95%CI)	HR (95%CI)	HR (95%CI)
**AHEI**						
Unhealthy tertile	3043/50543	613912	Ref.	Ref.	Ref.	Ref.
Medium tertile	2733/50542	615414	0.90 (0.85-0.94)	0.92 (0.87-0.97)	0.92 (0.87-0.97)	0.92 (0.87-0.97)
Healthy tertile	2455/50543	615206	0.84 (0.79-0.89)	0.86 (0.81-0.91)	0.87 (0.82-0.92)	0.87 (0.82-0.92)
P for trend			<0.001	<0.001	<0.001	<0.001
AIC			134801.9	133773.3	133561.2	133484.5
BIC			134844.0	133976.8	133778.7	133702.0
**DASH**						
Unhealthy tertile	3273/54227	659542	Ref.	Ref.	Ref.	Ref.
Medium tertile	2705/49349	599911	0.89 (0.85-0.94)	0.93 (0.88-0.98)	0.94 (0.89-0.99)	0.94 (0.89-0.99)
Healthy tertile	2253/48002	585081	0.79 (0.75-0.83)	0.84 (0.79-0.88)	0.85 (0.80-0.89)	0.85 (0.80-0.90)
P for trend			<0.001	<0.001	<0.001	<0.001
AIC			134771.1	133760.9	133550.5	133473.9
BIC			134813.2	133964.3	133768.0	133691.4
**MED**						
Unhealthy tertile	3109/53299	647121	Ref.	Ref.	Ref.	Ref.
Medium tertile	3460/64070	779297	0.86 (0.82-0.90)	0.91 (0.86-0.95)	0.91 (0.87-0.96)	0.91 (0.87-0.96)
Healthy tertile	1662/34259	418115	0.75 (0.70-0.79)	0.82 (0.77-0.87)	0.83 (0.78-0.88)	0.83 (0.78-0.88)
P for trend			<0.001	<0.001	<0.001	<0.001
AIC			134747.5	133757.6	133546.0	133470.0
BIC			134789.6	133961.1	133763.5	133687.4
**DII**						
Healthy tertile	2793/50543	615201	Ref.	Ref.	Ref.	Ref.
Medium tertile	2574/50542	616391	0.99 (0.94-1.05)	1.01 (0.95-1.07)	1.01 (0.95-1.07)	1.01 (0.95-1.07)
Unhealthy tertile	2864/50543	612940	1.19 (1.13-1.25)	1.16 (1.09-1.24)	1.15 (1.08-1.23)	1.14 (1.07-1.22)
P for trend			<0.001	<0.001	<0.001	<0.001

AIC			134787.7	133772.7	133560.4	133485.4
BIC			134829.8	133976.2	133777.9	133702.9
**PDI**						
Unhealthy tertile	3495/59537	722493	Ref.	Ref.	Ref.	Ref.
Medium tertile	2392/45210	550542	0.91 (0.87-0.96)	0.94 (0.89-0.99)	0.94 (0.89-0.99)	0.94 (0.89-0.99)
Healthy tertile	2344/46881	571498	0.87 (0.83-0.92)	0.90 (0.85-0.95)	0.90 (0.85-0.95)	0.90 (0.86-0.95)
P for trend			<0.001	<0.001	<0.001	<0.001
AIC			134814.9	133785.0	133569.3	133493.7
BIC			134857.0	133988.5	133786.8	133711.2
**HPDI**						
Unhealthy tertile	3362/55751	677816	Ref.	Ref.	Ref.	Ref.
Medium tertile	2528/46252	562282	0.92 (0.88-0.97)	0.95 (0.90-1.00)	0.96 (0.91-1.01)	0.96 (0.91-1.01)
Healthy tertile	2341/49625	604435	0.84 (0.80-0.89)	0.88 (0.83-0.93)	0.89 (0.84-0.94)	0.89 (0.84-0.94)
P for trend			<0.001	<0.001	<0.001	<0.001
AIC			134804.6	133780.3	133567.8	133491.2
BIC			134846.7	133983.7	133785.3	133708.6
**UPDI**						
Healthy tertile	2681/53591	653772	Ref.	Ref.	Ref.	Ref.
Medium tertile	2611/48237	586892	1.12 (1.06-1.18)	1.09 (1.03-1.15)	1.09 (1.03-1.15)	1.09 (1.03-1.15)
Unhealthy tertile	2939/49800	603868	1.31 (1.25-1.39)	1.24 (1.17-1.31)	1.22 (1.16-1.29)	1.22 (1.15-1.29)
P for trend			<0.001	<0.001	<0.001	<0.001
AIC			134740.0	133740.3	133531.3	133456.0
BIC			134782.1	133943.7	133748.7	133673.5

Model 1: adjusted for age at recruitment (strata), sex, assessment center (strata), body mass index, and ethnicity. Model 2: further adjusted for education, employment, household income, Townsend deprivation index, smoking status, alcohol drinking frequency, sleep duration, physical activity, energy, family history of diabetes, family history of CVD, family history of cancer. AHEI, Alternative Healthy Eating Index; DASH, Dietary Approaches to Stop Hypertension; MED, Mediterranean Diet; DII, Dietary Inflammatory Index; PDI, Plant-Based Diet Index; HPDI, Healthy Plant-Based Diet Index; UPDI, Unhealthy Plant-Based Diet Index; CVD, cardiovascular disease; HR, hazard ratios; CI, confidence interval; SD: standard deviation; AIC, Akaike information criterion; BIC, Bayesian information criterion.

Supplementary Figure S1. Flowchart of participant enrolment

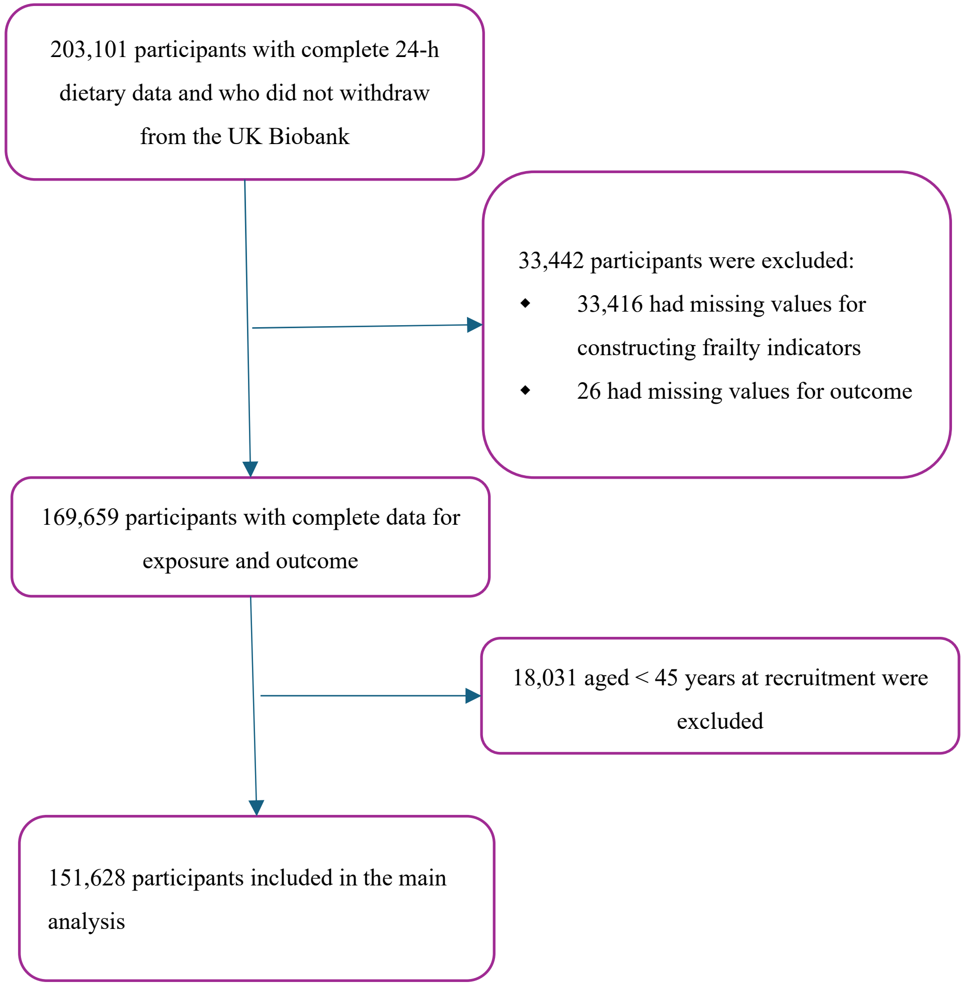

Supplementary Figure S2. Joint associations of frailty status and diet quality scores (modified) with all-cause mortality

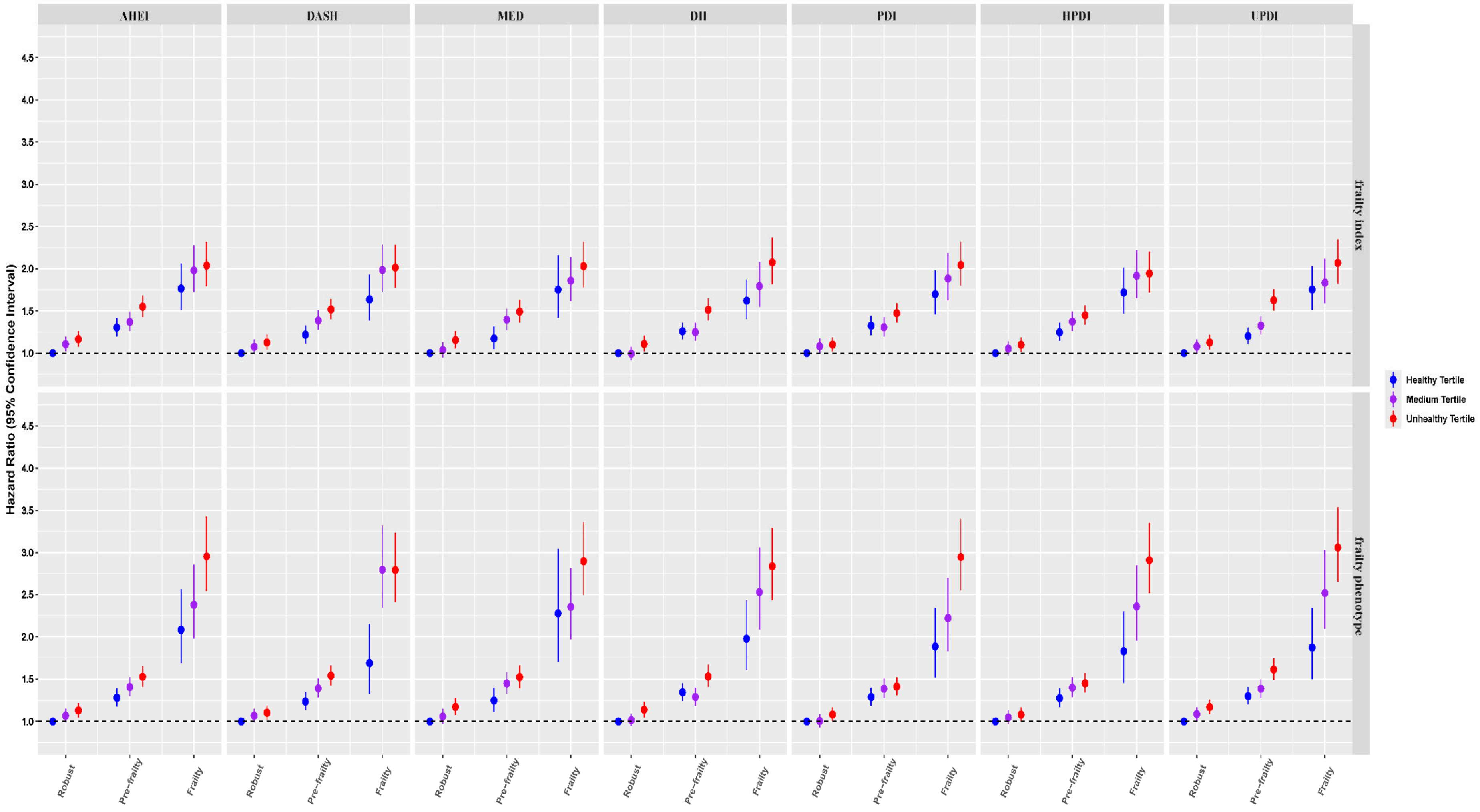

Diet quality scores were categorized into healthy (AHEI, DASH, MED, PDI, HPDI) and unhealthy (DII, UPDI) diets. The healthy tertile was defined as the top tertile of the healthy diet or the low tertile of the unhealthy diet, while the unhealthy tertile was defined as the low tertile of the healthy diet or the top tertile of the unhealthy diet.

Model was adjusted for age at recruitment (strata), sex, assessment center (strata), body mass index, ethnicity, education, employment, household income, Townsend deprivation index, smoking status, alcohol drinking frequency, physical activity, energy, sleep duration, family history of diabetes, family history of CVD, and family history of cancer. AHEI, Alternative Healthy Eating Index; DASH, Dietary Approaches to Stop Hypertension; MED, Mediterranean Diet; DII, Dietary Inflammatory Index; PDI, Plant-Based Diet Index; HPDI, Healthy Plant-Based Diet Index; UPDI, Unhealthy Plant-Based Diet Index; CVD, cardiovascular disease; HR, hazard ratios; CI, confidence interval. AHEI and MED removed item related to alcohol, and DII removed items related to alcohol and energy.

Supplementary Figure S3. Dose–response associations (HR and 95%CI) between diet quality scores (modified) with all-cause mortality by frailty status using restricted cubic splines with four knots located at the 5th, 35th, 65th, and 95th percentiles of each exposure

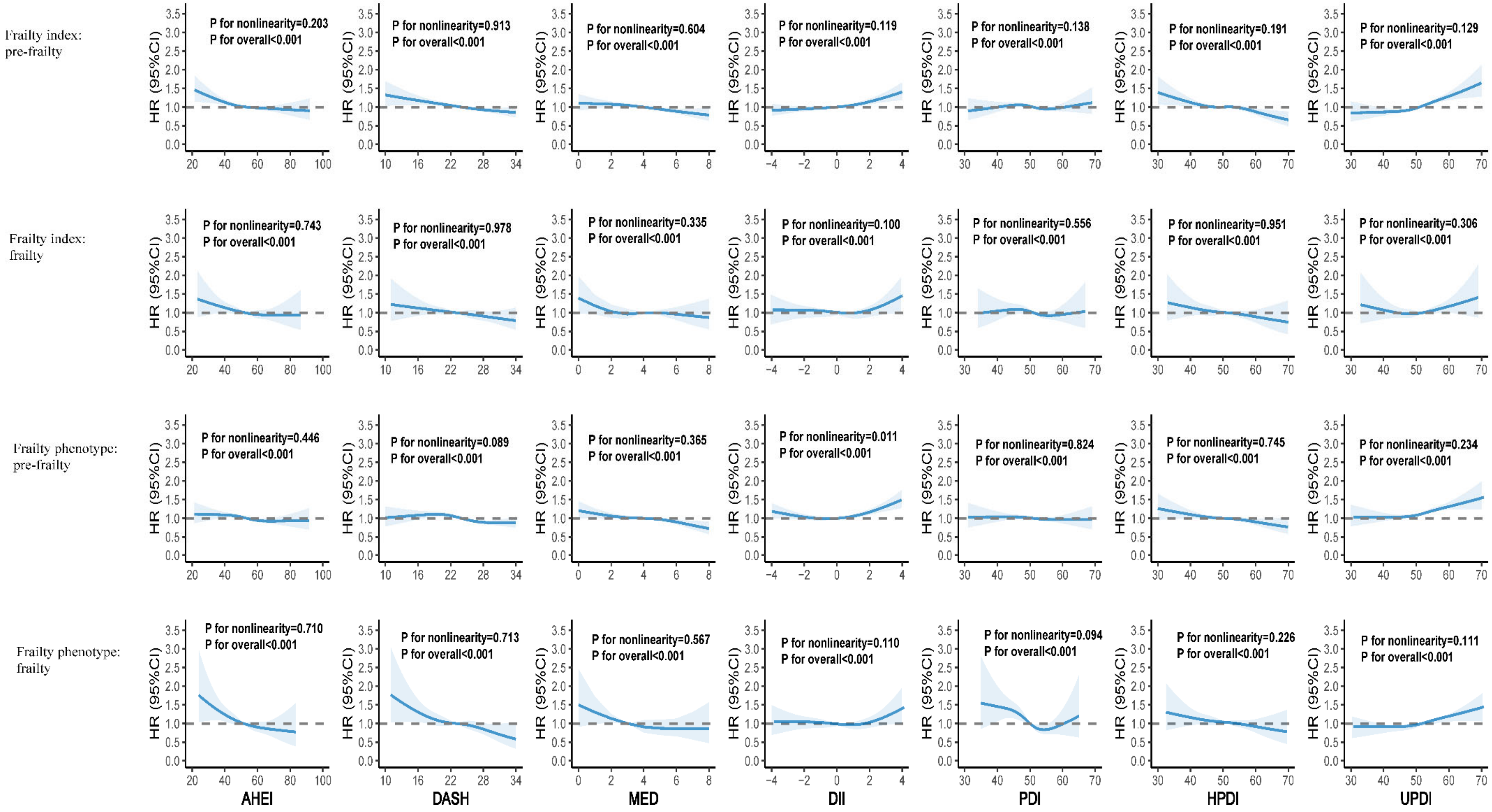

Model was adjusted for age at recruitment (strata), sex, assessment center (strata), body mass index, ethnicity, education, employment, household income, Townsend deprivation index, smoking status, alcohol drinking frequency, physical activity, energy, sleep duration, family history of diabetes, family history of CVD, and family history of cancer. AHEI, Alternative Healthy Eating Index; DASH, Dietary Approaches to Stop Hypertension; MED, Mediterranean Diet; DII, Dietary Inflammatory Index; PDI, Plant-Based Diet Index; HPDI, Healthy Plant-Based Diet Index; UPDI, Unhealthy Plant-Based Diet Index; CVD, cardiovascular disease; HR, hazard ratios; CI, confidence interval. AHEI and MED removed item related to alcohol, and DII removed items related to alcohol and energy.

Supplementary Figure S4. Gender-specific analysis of diet quality scores (modified) and life expectancy disparity by frailty index status

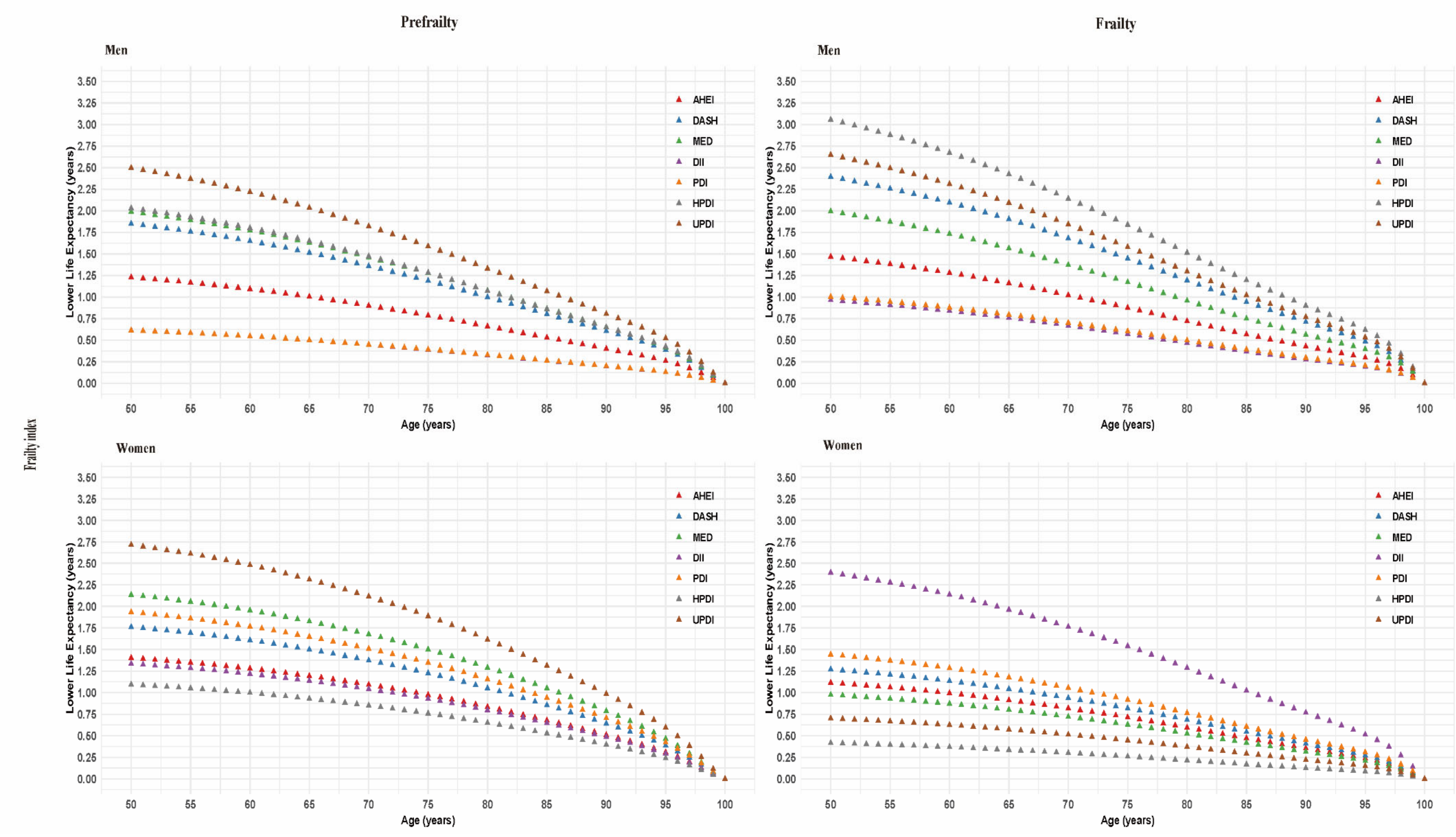

Diet quality scores were categorized as healthy (AHEI, DASH, MED, PDI, HPDI) and unhealthy (DII, UPDI). The healthy tertile was defined as the top tertile of the healthy diet or the low tertile of the unhealthy diet, while the unhealthy tertile was defined as the low tertile of the healthy diet or the top tertile of the unhealthy diet. Lower life expectancy was calculated by comparing the estimated life expectancy of individuals in healthy tertile to those in the unhealthy tertile. Model adjusted for age at recruitment, sex, assessment center, body mass index, and ethnicity, education, employment, household income, Townsend deprivation index, smoking status, alcohol drinking frequency, sleep duration, physical activity, energy, family history of diabetes, family history of CVD, family history of cancer. AHEI, Alternative Healthy Eating Index; DASH, Dietary Approaches to Stop Hypertension; MED, Mediterranean Diet; DII, Dietary Inflammatory Index; PDI, Plant-Based Diet Index; HPDI, Healthy Plant-Based Diet Index; UPDI, Unhealthy Plant-Based Diet Index; CVD, cardiovascular disease; HR, hazard ratios; CI, confidence interval. AHEI and MED removed item related to alcohol, and DII removed items related to alcohol and energy.

Supplementary Figure S5. Gender-specific analysis of diet quality scores (modified) and life expectancy disparity by frailty phenotype status

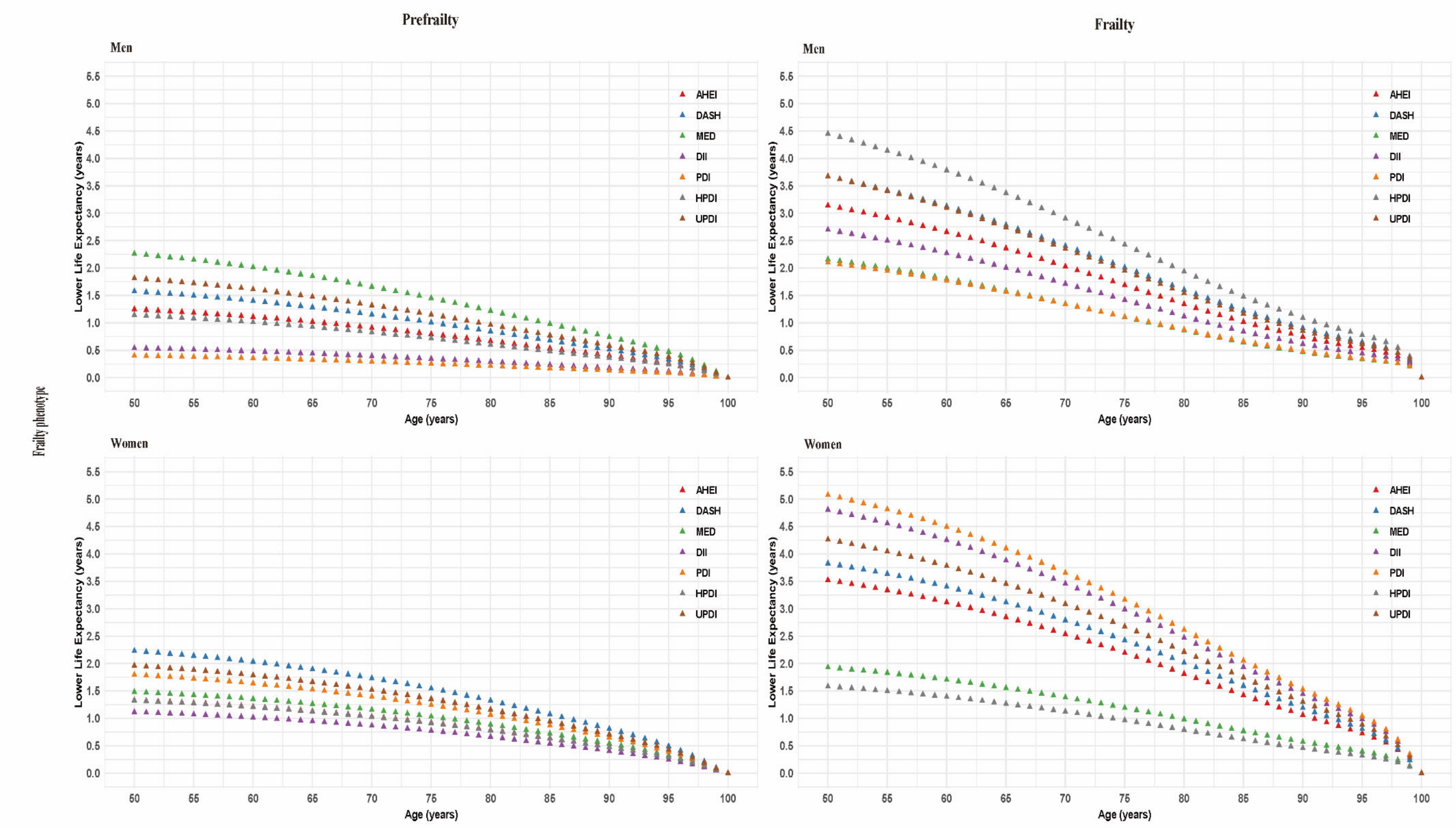

Diet quality scores were categorized as healthy (AHEI, DASH, MED, PDI, HPDI) and unhealthy (DII, UPDI). The healthy tertile was defined as the top tertile of the healthy diet or the low tertile of the unhealthy diet, while the unhealthy tertile was defined as the low tertile of the healthy diet or the top tertile of the unhealthy diet. Lower life expectancy was calculated by comparing the estimated life expectancy of individuals in healthy tertile to those in the unhealthy tertile. Model adjusted for age at recruitment, sex, assessment center, body mass index, and ethnicity, education, employment, household income, Townsend deprivation index, smoking status, alcohol drinking frequency, sleep duration, physical activity, energy, family history of diabetes, family history of CVD, family history of cancer. AHEI, Alternative Healthy Eating Index; DASH, Dietary Approaches to Stop Hypertension; MED, Mediterranean Diet; DII, Dietary Inflammatory Index; PDI, Plant-Based Diet Index; HPDI, Healthy Plant-Based Diet Index; UPDI, Unhealthy Plant-Based Diet Index; CVD, cardiovascular disease; HR, hazard ratios; CI, confidence interval. AHEI and MED removed item related to alcohol, and DII removed items related to alcohol and energy.

Supplementary Figure S6. Joint associations of frailty status and diet quality scores with all-cause mortality (remove death in the first 2 years)

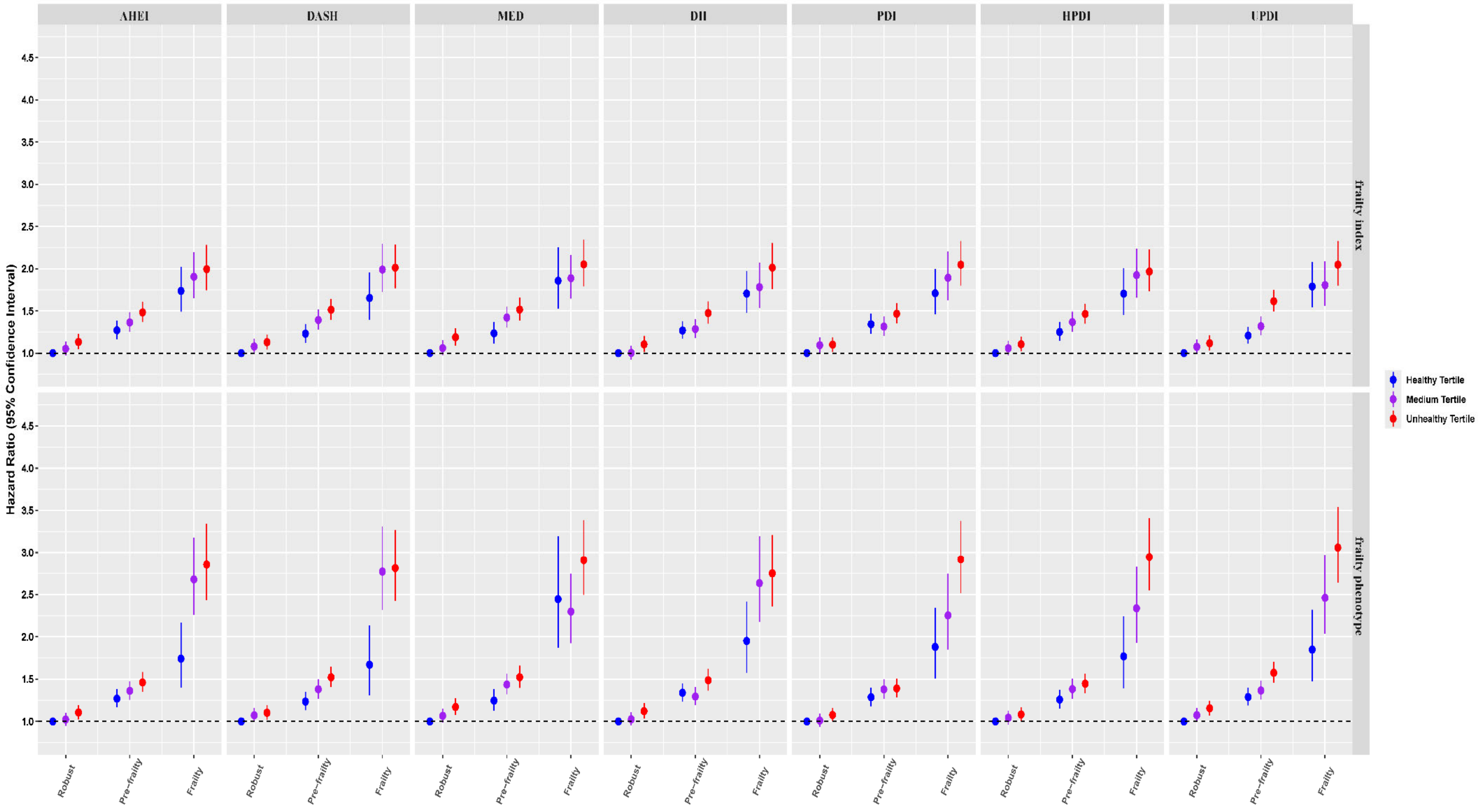

Diet quality scores were categorized into healthy (AHEI, DASH, MED, PDI, HPDI) and unhealthy (DII, UPDI) diets. The healthy tertile was defined as the top tertile of the healthy diet or the low tertile of the unhealthy diet, while the unhealthy tertile was defined as the low tertile of the healthy diet or the top tertile of the unhealthy diet.

Model was adjusted for age at recruitment (strata), sex, assessment center (strata), body mass index, ethnicity, education, employment, household income, Townsend deprivation index, smoking status, alcohol drinking frequency, physical activity, energy, sleep duration, family history of diabetes, family history of CVD, and family history of cancer. AHEI, Alternative Healthy Eating Index; DASH, Dietary Approaches to Stop Hypertension; MED, Mediterranean Diet; DII, Dietary Inflammatory Index; PDI, Plant-Based Diet Index; HPDI, Healthy Plant-Based Diet Index; UPDI, Unhealthy Plant-Based Diet Index; CVD, cardiovascular disease; HR, hazard ratios; CI, confidence interval.

Supplementary Figure S7. Joint associations of frailty status and diet quality scores with all-cause mortality (remove missing values)

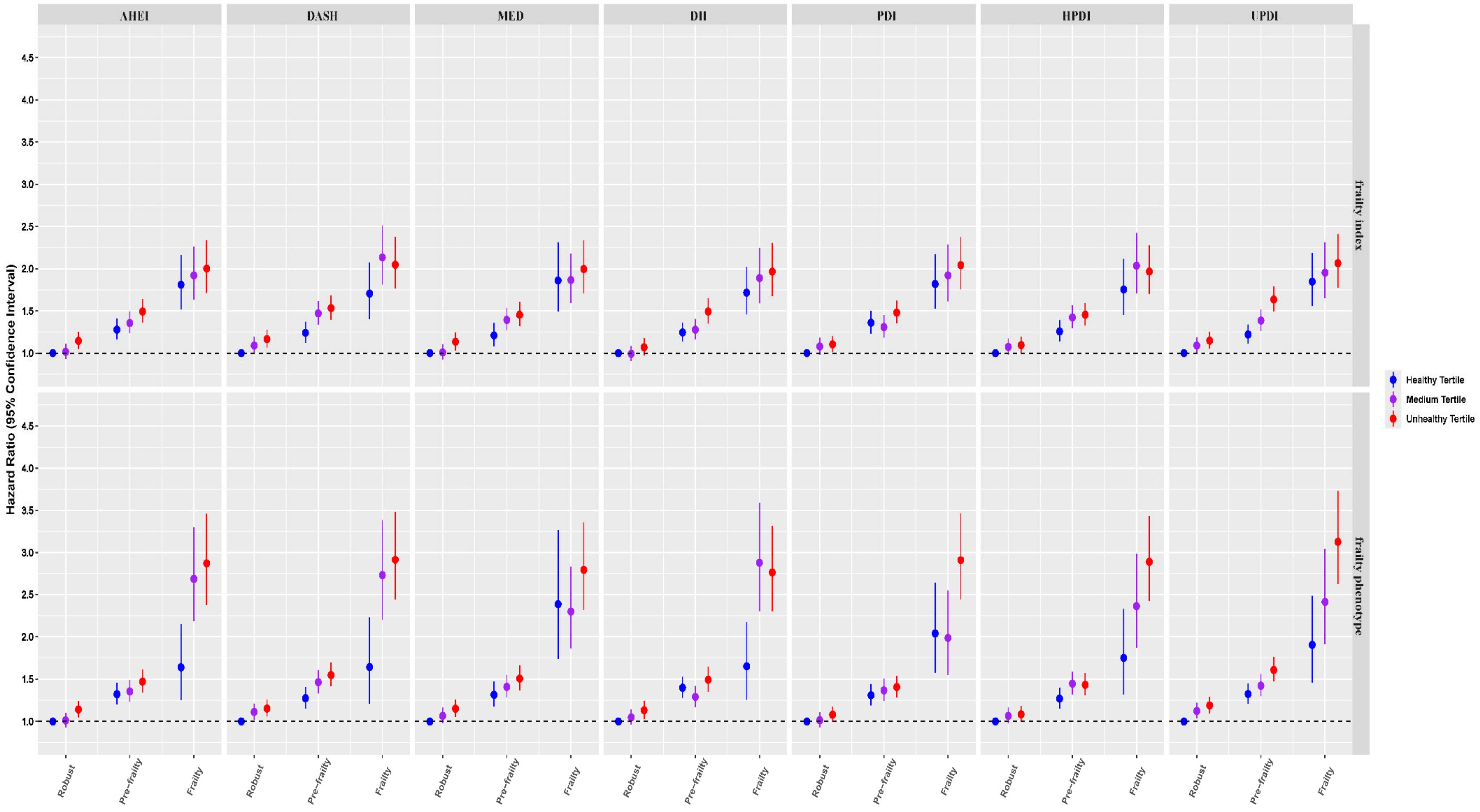

Diet quality scores were categorized into healthy (AHEI, DASH, MED, PDI, HPDI) and unhealthy (DII, UPDI) diets. The healthy tertile was defined as the top tertile of the healthy diet or the low tertile of the unhealthy diet, while the unhealthy tertile was defined as the low tertile of the healthy diet or the top tertile of the unhealthy diet.
Model was adjusted for age at recruitment (strata), sex, assessment center (strata), body mass index, ethnicity, education, employment, household income, Townsend deprivation index, smoking status, alcohol drinking frequency, physical activity, energy, sleep duration, family history of diabetes, family history of CVD, and family history of cancer. AHEI, Alternative Healthy Eating Index; DASH, Dietary Approaches to Stop Hypertension; MED, Mediterranean Diet; DII, Dietary Inflammatory Index; PDI, Plant-Based Diet Index; HPDI, Healthy Plant-Based Diet Index; UPDI, Unhealthy Plant-Based Diet Index; CVD, cardiovascular disease; HR, hazard ratios; CI, confidence interval.

Supplementary Figure S8. Joint associations of frailty status and diet quality scores with all-cause mortality (MICE imputation)

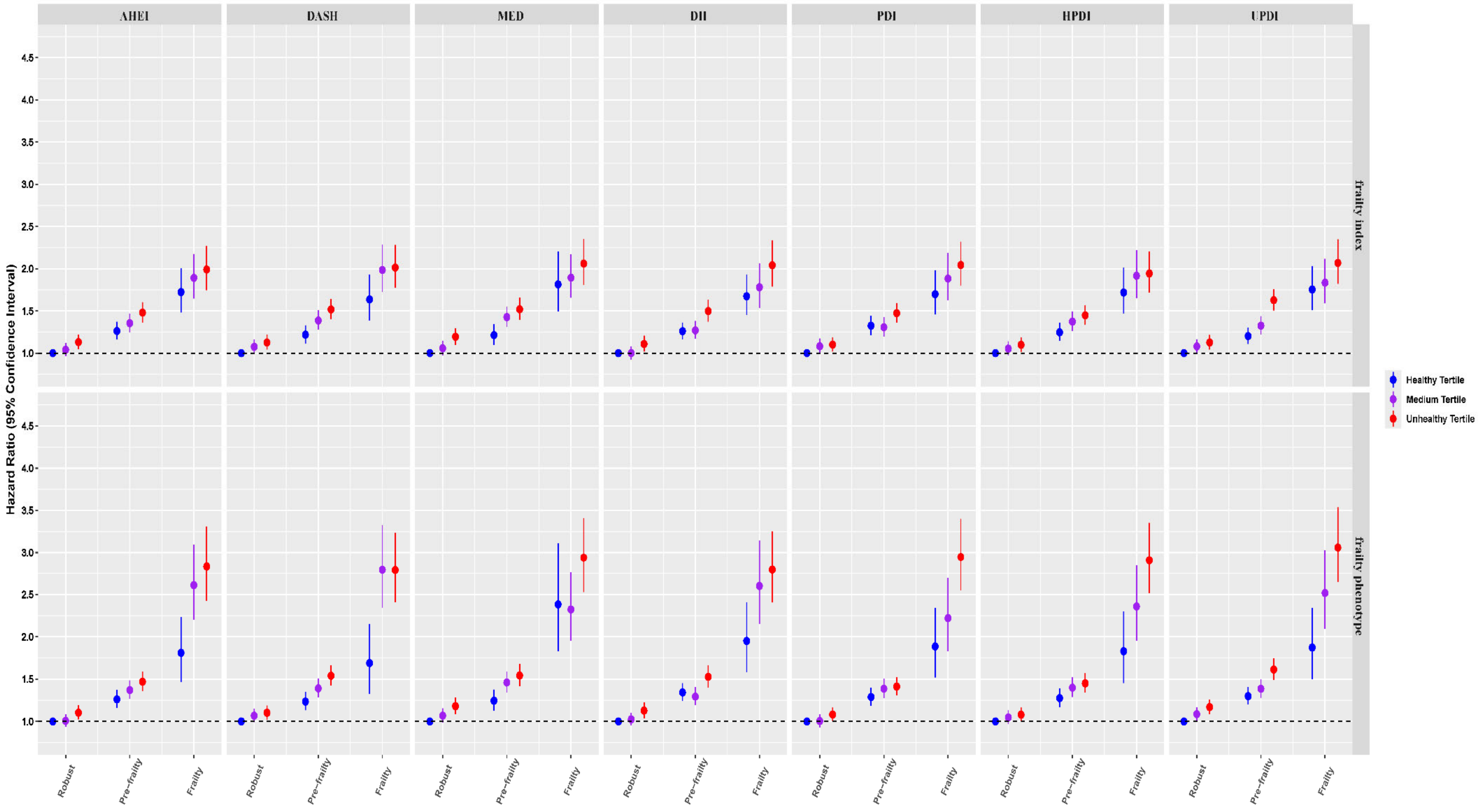

Diet quality scores were categorized into healthy (AHEI, DASH, MED, PDI, HPDI) and unhealthy (DII, UPDI) diets. The healthy tertile was defined as the top tertile of the healthy diet or the low tertile of the unhealthy diet, while the unhealthy tertile was defined as the low tertile of the healthy diet or the top tertile of the unhealthy diet.

Model was adjusted for age at recruitment (strata), sex, assessment center (strata), body mass index, ethnicity, education, employment, household income, Townsend deprivation index, smoking status, alcohol drinking frequency, physical activity, energy, sleep duration, family history of diabetes, family history of CVD, and family history of cancer. AHEI, Alternative Healthy Eating Index; DASH, Dietary Approaches to Stop Hypertension; MED, Mediterranean Diet; DII, Dietary Inflammatory Index; PDI, Plant-Based Diet Index; HPDI, Healthy Plant-Based Diet Index; UPDI, Unhealthy Plant-Based Diet Index; CVD, cardiovascular disease; HR, hazard ratios; CI, confidence interval.

Supplementary Figure S9. Joint associations of frailty status and diet quality scores with all-cause mortality (further adjusted for medications)

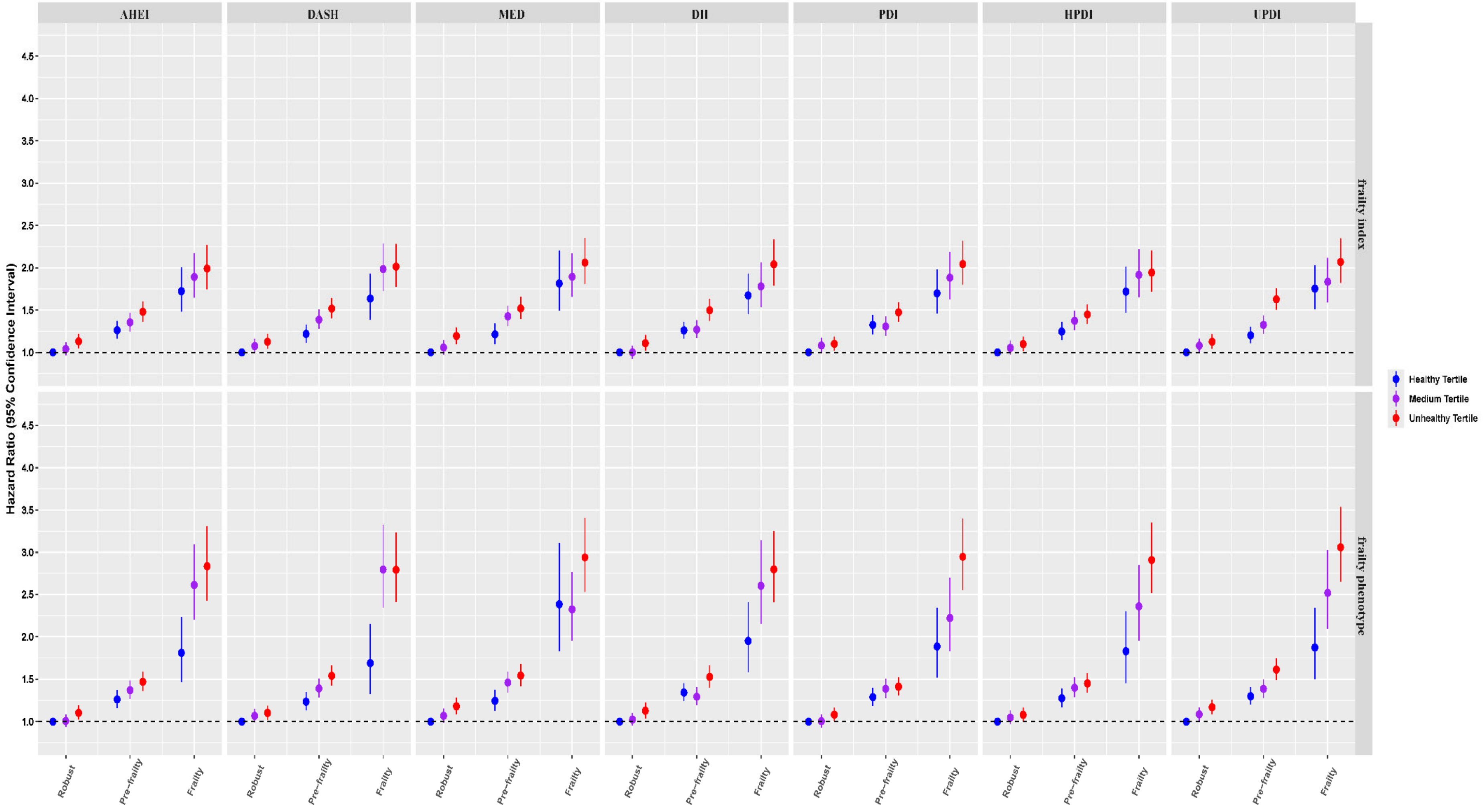

Diet quality scores were categorized into healthy (AHEI, DASH, MED, PDI, HPDI) and unhealthy (DII, UPDI) diets. The healthy tertile was defined as the top tertile of the healthy diet or the low tertile of the unhealthy diet, while the unhealthy tertile was defined as the low tertile of the healthy diet or the top tertile of the unhealthy diet.
Model was adjusted for age at recruitment (strata), sex, assessment center (strata), body mass index, ethnicity, education, employment, household income, Townsend deprivation index, smoking status, alcohol drinking frequency, physical activity, energy, sleep duration, family history of diabetes, family history of CVD, family history of cancer, blood pressure medication, cholesterol-lowering medication, and insulin. AHEI, Alternative Healthy Eating Index; DASH, Dietary Approaches to Stop Hypertension; MED, Mediterranean Diet; DII, Dietary Inflammatory Index; PDI, Plant-Based Diet Index; HPDI, Healthy Plant-Based Diet Index; UPDI, Unhealthy Plant-Based Diet Index; CVD, cardiovascular disease; HR, hazard ratios; CI, confidence interval.

Supplementary Figure S10. Joint associations of frailty status and diet quality scores with all-cause mortality (further adjusted for overall health status)

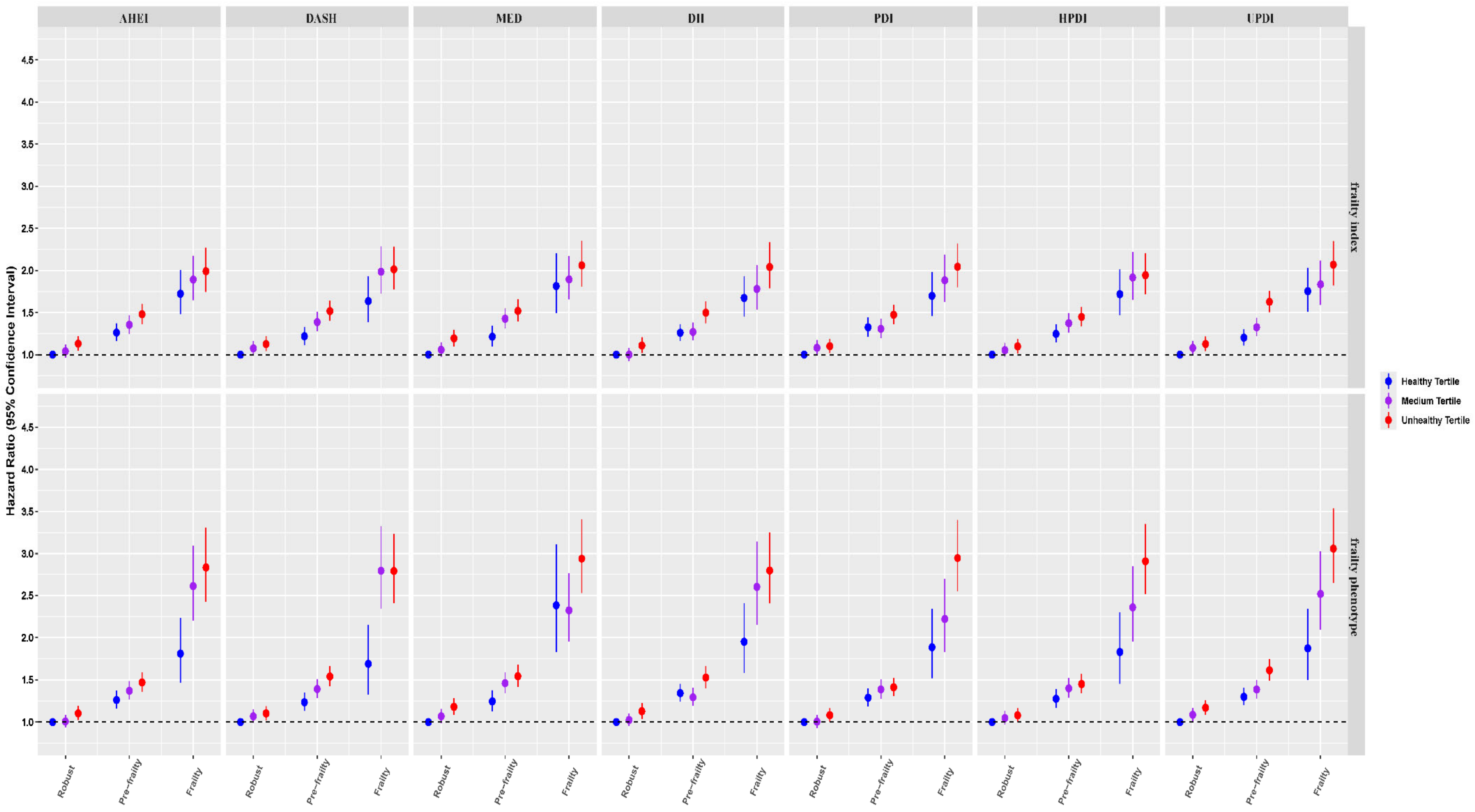

Diet quality scores were categorized into healthy (AHEI, DASH, MED, PDI, HPDI) and unhealthy (DII, UPDI) diets. The healthy tertile was defined as the top tertile of the healthy diet or the low tertile of the unhealthy diet, while the unhealthy tertile was defined as the low tertile of the healthy diet or the top tertile of the unhealthy diet.
Model was adjusted for age at recruitment (strata), sex, assessment center (strata), body mass index, ethnicity, education, employment, household income, Townsend deprivation index, smoking status, alcohol drinking frequency, physical activity, energy, sleep duration, family history of diabetes, family history of CVD, family history of cancer, and overall health status. AHEI, Alternative Healthy Eating Index; DASH, Dietary Approaches to Stop Hypertension; MED, Mediterranean Diet; DII, Dietary Inflammatory Index; PDI, Plant-Based Diet Index; HPDI, Healthy Plant-Based Diet Index; UPDI, Unhealthy Plant-Based Diet Index; CVD, cardiovascular disease; HR, hazard ratios; CI, confidence interval.

Supplementary Figure S11. Cumulative risk of all-cause mortality by diet quality scores

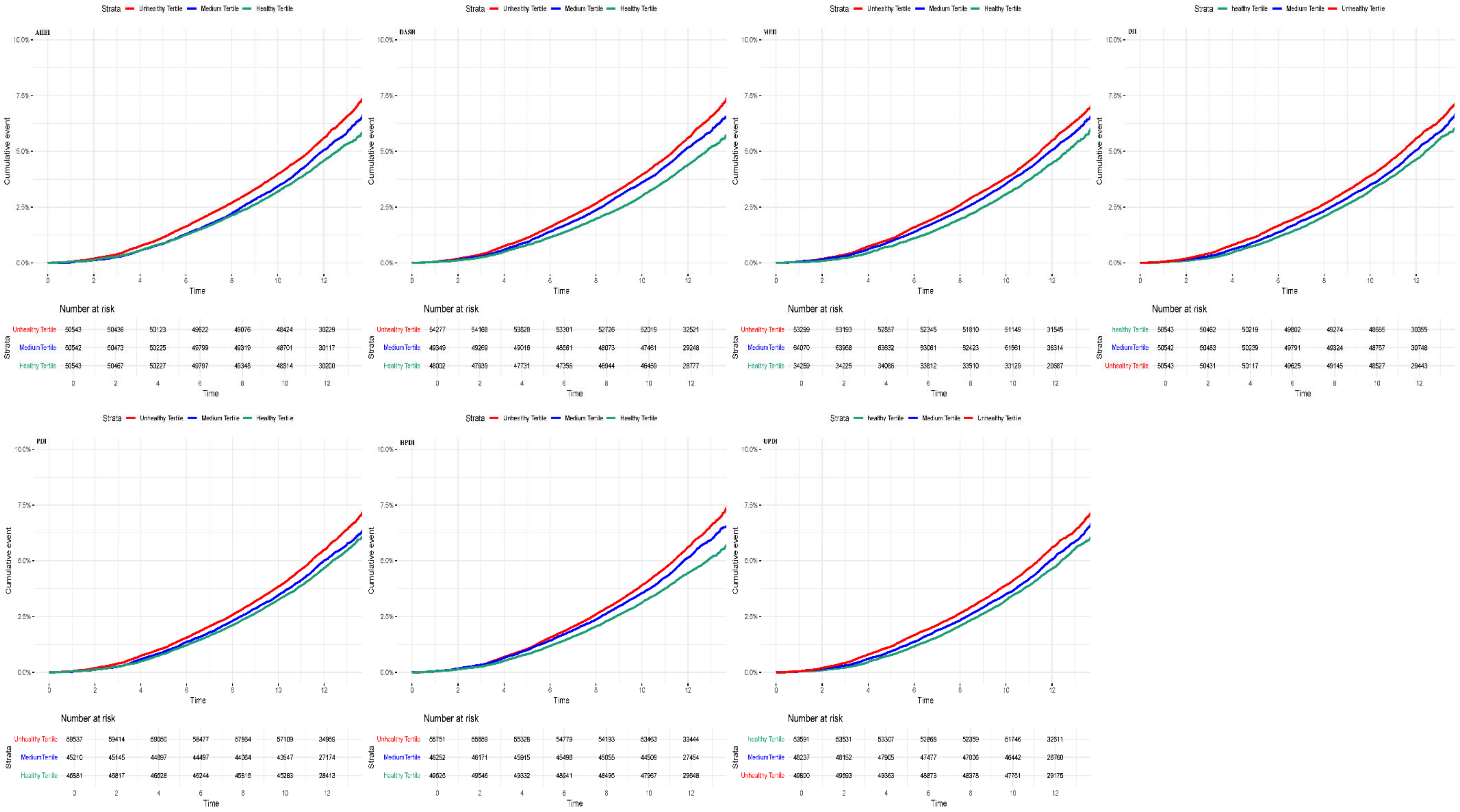

Supplementary Figure S12. Cumulative risk of all-cause mortality by frailty status

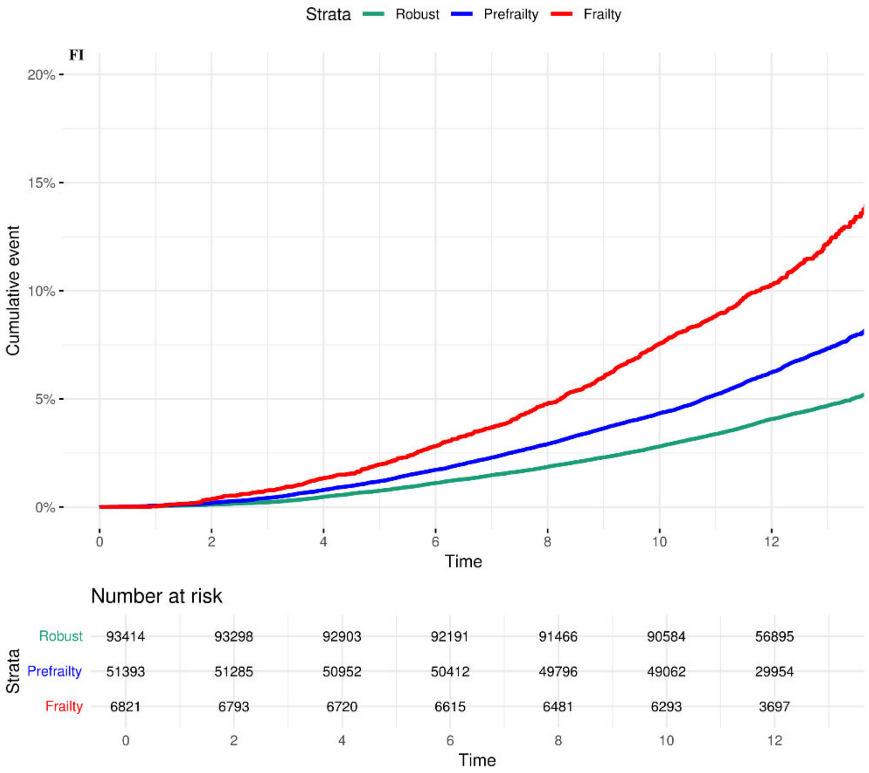

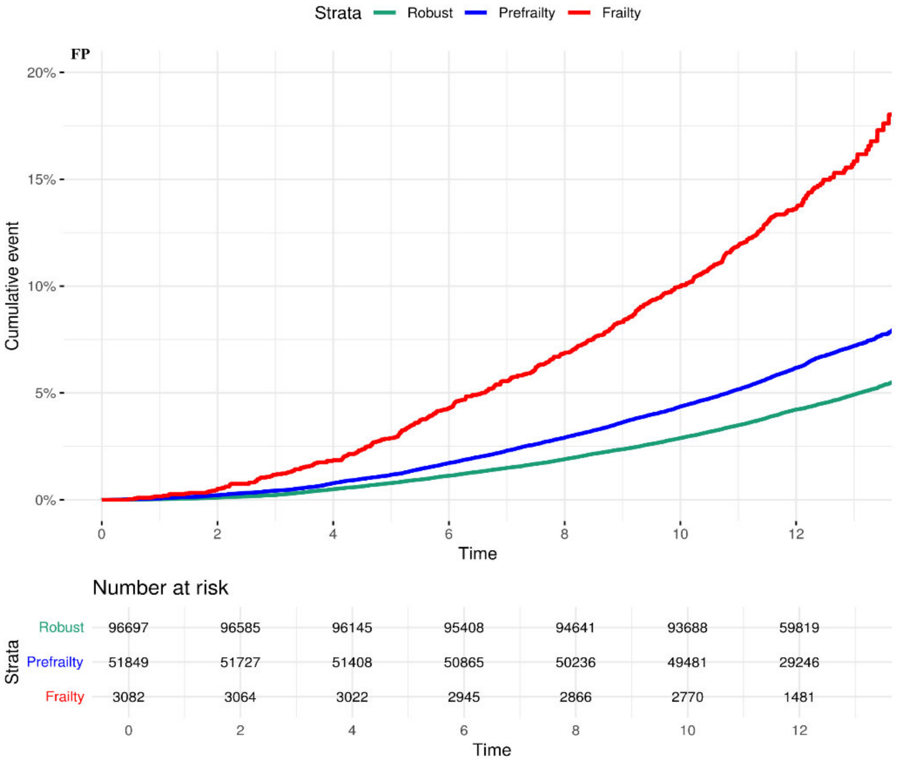

Supplement: Supplementary file 1 [file nutrients-17-03115-s001.zip › nutrients-3885554-supplementary.pdf]
